# Single-cell discovery of m^6^A RNA modifications in the hippocampus

**DOI:** 10.1101/gr.278424.123

**Published:** 2024-06

**Authors:** Shuangshuang Feng, Maitena Tellaetxe-Abete, Yujie Zhang, Yan Peng, Han Zhou, Mingjie Dong, Erika Larrea, Liang Xue, Li Zhang, Magdalena J. Koziol

**Affiliations:** 1 State Key Laboratory of Cognitive Neuroscience and Learning, Beijing Normal University, Beijing 100875, China;; 2 Chinese Institute for Brain Research, Beijing 102206, China;; 3 Research Unit of Medical Neurobiology, Chinese Academy of Medical Sciences, Beijing 102206, China;; 4 Intelligent Systems Group, Computer Science Faculty, University of the Basque Country, Donostia/San Sebastian 20018, Spain;; 5 Peking University, Beijing, 100871, China;; 6 Tsinghua University, Beijing 100084, China

## Abstract

*N*^6^-Methyladenosine (m^6^A) is a prevalent and highly regulated RNA modification essential for RNA metabolism and normal brain function. It is particularly important in the hippocampus, where m^6^A is implicated in neurogenesis and learning. Although extensively studied, its presence in specific cell types remains poorly understood. We investigated m^6^A in the hippocampus at a single-cell resolution, revealing a comprehensive landscape of m^6^A modifications within individual cells. Through our analysis, we uncovered transcripts exhibiting a dense m^6^A profile, notably linked to neurological disorders such as Alzheimer's disease. Our findings suggest a pivotal role of m^6^A-containing transcripts, particularly in the context of CAMK2A neurons. Overall, this work provides new insights into the molecular mechanisms underlying hippocampal physiology and lays the foundation for future studies investigating the dynamic nature of m^6^A RNA methylation in the healthy and diseased brain.

The hippocampus, crucial for learning and memory (Bird and Burgess 2008), is a focal point in neuroscience research. *N*^6^-Methyladenosine (m^6^A) RNA methylation, a key epitranscriptomic modification, regulates various biological processes, including neurodevelopment and learning (Li et al. 2017; Livneh et al. 2020; Jiang et al. 2021; Yu et al. 2021), with implications for neurological disorders (Lv et al. 2023).

The m^6^A modification is catalyzed by methyltransferase 3, N6-adenosine-methyltransferase complex catalytic subunit (METTL3) and is reversible by enzymes like FTO alpha-ketoglutarate dependent dioxygenase (FTO) and ALKBH5 (Li et al. 2017). m^6^A-modified transcripts are bound by reader proteins such as YTH N^6^-methyladenosine RNA binding protein F2 (YTHDF2) (Wang et al. 2014; Du et al. 2016).

Although m^6^A in various tissues is well studied, despite its presence and impact in the hippocampus, it is currently unknown which cell types and transcripts have m^6^A and with which density (Zhou et al. 2023).

Studies on *Mettl3*, *Ythdf2*, and *Fto* knockout (KO) mice link m^6^A to hippocampal function, learning, and memory (Li et al. 2017; Engel et al. 2018; Livneh et al. 2020; Zhuang et al. 2023). Also, overexpression of *Mettl3* enhances long-term memory consolidation (Zhang et al. 2018; Jiang et al. 2021). Cell-specific m^6^A levels have been noted in the cerebellum and cortex, suggesting potential cell type–specific functions (Chang et al. 2017).

To uncover the landscape of m^6^A RNA methylation in hippocampal cells, we employ single-cell sequencing. Single-cell sequencing technologies have revolutionized our ability to uncover cellular heterogeneity and identify distinct subpopulations within complex tissues (Regev et al. 2017). Applying these technologies to the study of m^6^A will provide unprecedented insights into the cell type–specific roles of m^6^A in hippocampal function, facilitating the development of future targeted therapeutics.

In this study, we aim to address the knowledge gap by performing single-cell RNA sequencing to profile m^6^A RNA methylation patterns in individual cells of the hippocampus. A myriad of different m^6^A sequencing detection methods exist, such as m^6^A RNA immunoprecipitation (m^6^A-RIP) (Dominissini et al. 2012; Meyer et al. 2012), coupled with high-throughput sequencing technologies, or m^6^A-SEAL (Wang et al. 2020) or MAZTER-seq (Garcia-Campos et al. 2019). However, all of them require high amounts of RNA input, currently making them incompatible with single-cell approaches in regular somatic cells, such as found in the brain (Ke et al. 2015; Linder et al. 2015; Garcia-Campos et al. 2019; Zhang et al. 2019; Shu et al. 2020; Wang et al. 2020; Yao et al. 2023; Li et al. 2024). Deamination adjacent to RNA modification targets sequencing (DART-seq), utilizing a APOBEC-YTH fusion construct in which the APOBEC1 protein is fused to the YTH m^6^A-binding domain of YTHDF2, enables m^6^A detection (Meyer 2019). In APOBEC-YTH, the YTH domain lures APOBEC1 to the close vicinity of m^6^A sites, where APOBEC1 deaminates cytidine into uracil (Fig. 1A; Meyer 2019). By identifying C-to-U editing events that correspond to C-to-T mutations in sequencing data, adjacent m^6^A sites can be identified. Ninety-seven percent of these sites disappeared in methylase METTL3-depleted cells, illustrating the specificity of this method (Meyer 2019). The feasibility of this single-cell DART-seq (scDART-seq) approach has recently also been demonstrated using a relatively homogenous HEK293T cell line (Tegowski et al. 2022). Here, we apply this single-cell approach to map m^6^A distribution in hippocampal cell types, enhancing our understanding of m^6^A modifications’ cell-specific characteristics.

**Figure 1.**
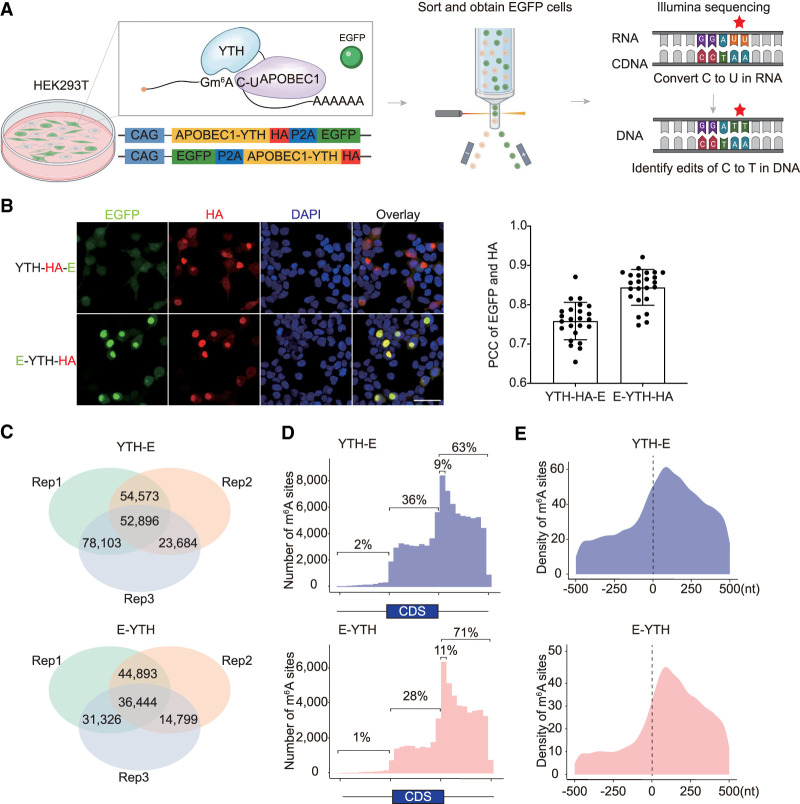
Improved m^6^A bulk RNA-seq detection in HEK293T cells. (*A*) Schematic diagram of m^6^A detection with bulk RNA-seq in cultured cells. The YTH protein domain binds to m^6^A. When bound to APOBEC1, the APOBEC1 protein converts C-to-U in the vicinity of m^6^A. This results in a C-to-T mutation in cDNA. C-to-T mutations detected by RNA sequencing are indicative of m^6^A RNA modifications. (*B*, *left*) Immunofluorescence (IF) of HEK293T cells transfected with *Apobec1-Yth-HA-Egfp* (*Yth-HA-E*) or *Egfp-Apobec1-Yth-HA (E-YTH-HA*). Scale bar, 20 μm. Representative images are shown. (*Right*) Quantification of EGFP and HA overlap. (PCC) Pearson correlation coefficient. (*C*) Number of C-to-T editing events identified in each bulk RNA-seq HEK293T cell replicate for *Yth-E* and *E-Yth* plasmids. Editing events identified in at least two replicates were considered for downstream analyses. The data were obtained following *Apobec1-Yth-Egfp* or *Egfp-Apobec1-Yth* transfection and EGFP FACS sorting. n = 3. (Rep) separately cultured replicate. (*D*) Metagene analysis showing m^6^A site counts along transcripts for *Yth-E* and *E-Yth* bulk RNA-seq results. Nine percent of all m^6^A sites occur in the first 10% of the 3′ UTR following the TTS for YTH-E and 11% for E-YTH, respectively. Shown percentage indicates number of m^6^A sites upstream of, within, and downstream from coding sequence (CDS). (*E*) Metagene analysis showing m^6^A density 500 nt 5′ and 500 nt 3′ from stop codon (0 nt) for YTH-E and E-YTH.

## Results

### Confirmation of m^6^A detection with bulk RNA-seq

To enable the application of DART-seq in mouse hippocampal cells in vivo, we subcloned *Apobec1-Yth* with an HA tag into an adeno-associated virus (AAV) plasmid under control of a chicken beta-actin (CAG) promoter. Controls included *Apobec1*-*Yth*^*mut*^, incapable of binding m^6^A, and *Apobec1* only. Through AAV transduction, these proteins can then be expressed in different cell types of the mouse brain (Negrini et al. 2020). A P2A sequence with *Egfp* was added to enable isolating successfully AAV-transduced cells without interfering with APOBEC1-YTH function. We argued that FACS selection for APOBEC1-YTH (YTH^mut^)-positive cells is crucial to avoid false negatives owing to inadequate APOBEC1-YTH exposure.

Testing the *Apobec1*-*Yth*-*HA*-*P2A*-*Egfp* (*Yth-E*) plasmid in HEK293T cells (subsequently referred to as the YTH-E sample) showed a weak enhanced green fluorescent protein (EGFP) signal (Fig. 1B), attributed to its location at the C-terminal end of a long RNA transcript (Zhu et al. 2023). Similarly, when *Yth*-*E* was transduced into the hippocampus, an EGFP signal could not be easily detected with FACS sorting (Fig. 1B; Supplemental Fig. S1A). Reordering the expression cassette to *Egfp*-*P2A*-*Apobec1*-*Yth*-*HA* (*E-Yth*) and *Egfp*-*P2A*-*Apobec1*-*Yth*^*mut*^-*HA* (*E*-*Yth*^*mut*^) resulted in enhanced EGFP signal and improved correlation with *Apobec1*-*Yth*-*HA* (Fig. 1B), suitable for single-cell sequencing. Both E-YTH and E-YTH^mut^ samples exhibited EGFP in cytoplasm and nuclei (Supplemental Fig. S1B), suggesting that both could be used for single-cell sequencing.

Further evaluation involved transfecting *E-Yth* and *Yth-E* into HEK293T cells for FACS sorting and RNA sequencing (RNA-seq) (Fig. 1A). As controls, we processed *E-Yth*^*mut*^, *Yth^mut^-E*, *E-Apobec1*, *Apobec1*-*E*, and mock-transfected cells. We only considered C-to-U editing sites that were identified in at least two replicates, such as 209,256 C-to-U editing sites in YTH-E and 127,462 in E-YTH samples (Fig. 1C; Supplemental Table S1), and did similar analysis for control samples (Supplemental Fig. S1C; Supplemental Table S1). To identify m^6^A sites in E-YTH and YTH-E, we eliminated background C-to-U editing events detected in APOBEC1-only, YTH^mut^, and mock-transfected cells (for more details, see Methods). We identified 106,827 m^6^A sites that occur in transcripts in YTH-E and 54,395 in E-YTH (Supplemental Table S2). In both cases, we found m^6^A to be enriched in the 3′ UTR region, 63% in YTH-E and 71% in E-YTH, confirming previous m^6^A findings (Fig. 1D,E; Supplemental Table S3; Dominissini et al. 2012; Meyer et al. 2012). Nine percent of all m^6^A sites in YTH-E and 11% in E-YTH occur in the first 10% of the 3′ UTR (Fig. 1D,E; Supplemental Table S3). Overall, YTH-E and E-YTH confirm previous m^6^A findings. E-YTH, with a stronger EGFP signal and clearer correlation with APOBEC1-YTH presence, was chosen for hippocampal studies owing to its defined m^6^A distribution patterns and reduced background noise (Fig. 1B,D; Supplemental Fig. S1D).

### Detection of m^6^A with bulk RNA-seq in mouse hippocampus

**Figure 2.**
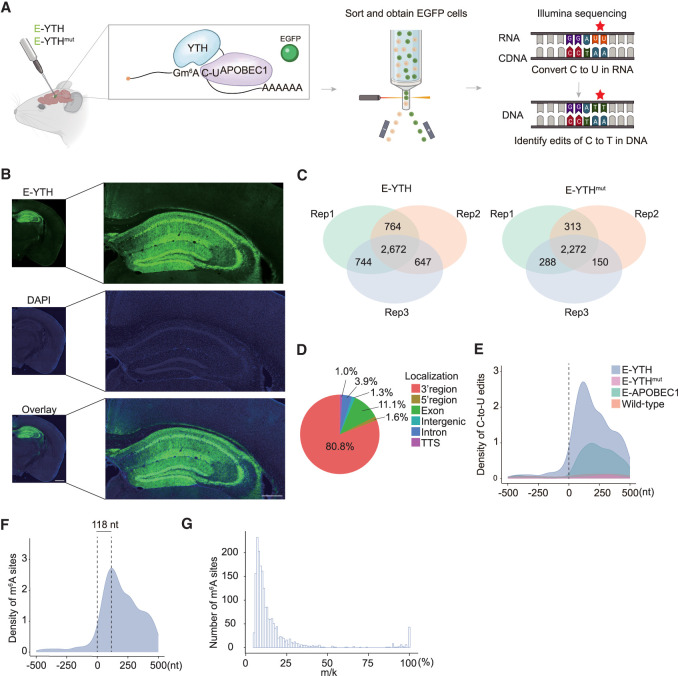
Detection of m^6^A with bulk RNA-seq in mouse hippocampus. (*A*) Schematic diagram of bulk RNA-seq in the mouse brain. The *Egfp-Apobec1-Yth* is packaged into AAV viruses to infect brain cells. EGFP-positive cells are isolated from the hippocampus and processed for C-to-U edit and m^6^A site identification. (*B*) Confocal image of mouse hippocampus after AAV infection. Representative image of E-YTH is shown. Half-brain image: Scale bar, 1 mm. Hippocampus image: Scale bar, 400 μm. (*C*) Number of overlapping C-to-U editing events identified by RNA-seq in hippocampus following *Egfp-Apobec1-Yth* and *Egfp-Apobec1-Yth*^*mut*^ AAV virus injection and EGFP FACS sorting. Editing events identified in at least two replicates were considered for downstream analyses. n = 3, (Rep) Biological replicates from different animals. (*D*) Pie chart showing m^6^A localization identified by *E-Yth* in mouse hippocampus. (TTS) Transcription termination site. (*E*) Metagene analysis showing C-to-U edit scaled density 500 nt 5′ and 500 nt 3′ from stop codon (0 nt) in E-YTH, E-YTH^mut^, E-APOBEC1, and wild-type (WT) samples. The peak value for E-YTH is 2716 editing events. (*F*) Metagene analysis showing m^6^A density 500 nt 5′ and 500 nt 3′ from stop codon (0 nt). m^6^A sites were obtain after eliminating background from E-YTH editing sites. m^6^A peak density occurs 118 nt downstream from the stop codon. (*G*) Histogram of m^6^A site counts over mutation per read (m/k) ratio. Minimum threshold: 5%.

Liquid chromatography coupled with tandem mass spectrometry (LC-MS/MS) of adult mouse brains showed higher levels of m^6^A in mRNAs in hippocampi than in mRNAs of the cortex, thalamus, or cerebellum. In hippocampal mRNAs, we found that m^6^A occurs in one per 1000 unmodified adenosines (Supplemental Fig. S2A). To identify m^6^A within hippocampal mRNA sequences, we tested if E-YTH can facilitate m^6^A detection in vivo. Thus, we packaged *E-Yth* and controls *E-Yth*^*mut*^ and *E-Apobec1* into AAV viruses for local hippocampal stereotaxic injection (Fig. 2A). Following successful AAV expression verification in the hippocampus (Fig. 2B), EGFP-positive cells underwent FACS sorting and RNA-seq (Fig. 2A). Wild-type (WT) hippocampal samples were included as additional controls; 2672 and 2272 C-to-U editing sites were identified in all three replicates of E-YTH and E-YTH^mut^, respectively. Only a few hundred C-to-U editing sites overlap between two replicates, whereas more are common between all three replicates, indicating high reproducibility (Fig. 2C; Supplemental Fig. S2B; Supplemental Table S4). To identify m^6^A sites, we focused C-to-U editing events present in at least two out of three replicates (Fig. 2C; Supplemental Table S4) and background from E-YTH, including those detected in WT, APOBEC1, and E-YTH^mut^ (see Methods) (Supplemental Fig. S2B; Supplemental Table S4). In total, we identified 1578 edits in E-YTH transcripts but only 82 edits in E-YTH^mut^ controls (Supplemental Table S5). To further verify the feasibility of m^6^A detection with E-YTH in the mouse hippocampus, we detected 8220 m^6^A regions that correspond to 5431 genes with m^6^A-RIP (Supplemental Fig. S2C; Supplemental Table S6). Thirty-two percent of the E-YTH m^6^A sites were also detected and corroborated with m^6^A-RIP (Supplemental Fig. S2D; Supplemental Table S7). In accordance to previous studies using m^6^A-RIP and DART-seq in cells (Meyer et al. 2012), our E-YTH identified m^6^A enrichment in the 3′ region (80.8%) (Fig. 2D; Supplemental Fig. S2E), particularly near the TTS (Fig. 2E). We detected that most m^6^A occur 118 nt downstream from the TTS site (Fig. 2F). To assess the frequency of m^6^A occurrence across multiple transcript copies from a single gene (referred to as RNA replicates), we calculated the mutation per read ratio (m/k) for each editing site, excluding sites with fewer than 10 reads (see Methods). A m/k ratio of one indicates that all copies of a specific transcript have m^6^A, notably at the same site, whereas a m/k ratio of 0.25 stipulates that 25% of them share m^6^A. Our bulk sequencing data have low m^6^A density at each position. Although some transcripts exhibit m^6^A on all their RNA copies (m/k = 1), the majority have m^6^A in <10% (Fig. 2G; Supplemental Fig. S1D). The bulk sequencing data suggest that m^6^A, although functionally crucial in the hippocampus (Zhang et al. 2018; Du et al. 2021; Yin et al. 2023), is predominantly heterogenous at specific sites. However, certain cells have sites where m^6^A occurs on many RNA replicates. To investigate this, we next applied our system to detect m^6^A sites on a single-cell level in the mouse hippocampus.

### Single-cell sequencing of E-YTH-transduced hippocampus

**Figure 3.**
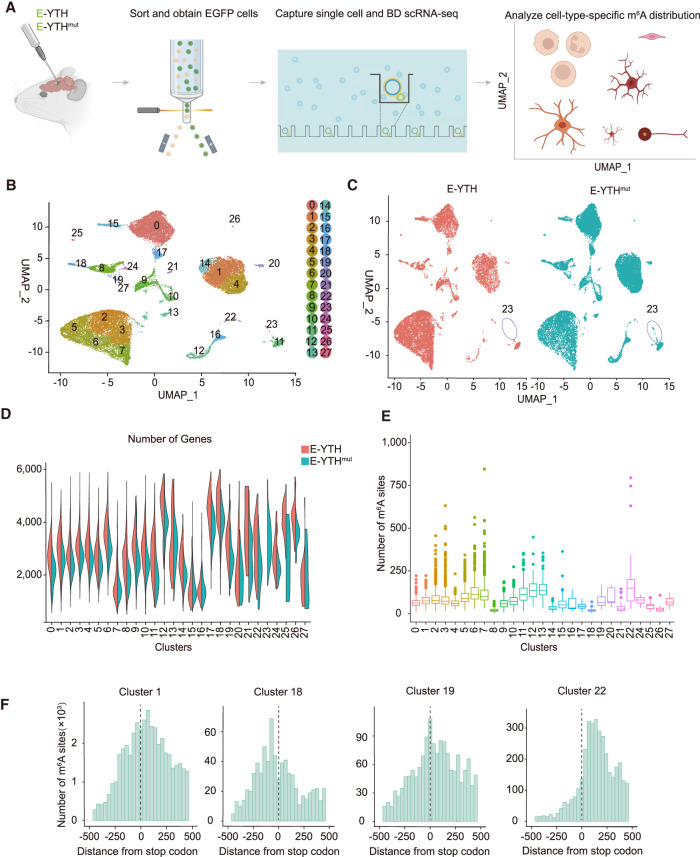
Hippocampal single-cell identification following AAV transduction. (*A*) Schematic diagram of detecting m^6^A RNA modification in the mouse hippocampus on a single-cell level through AAV transduction with *E-Yth* and *E-Yth*^*mut*^ controls. (*B*) Integration of uniform manifold approximation and projection (UMAP) of 27,804 single-cell transcriptomes. Cluster numbers from zero to 27 are indicated. (*C*) Separate UMAP for E-YTH and E-YTH^mut^: 11,561 single cells in E-YTH and 16,243 in E-YTH^mut^. (Circle) Cluster 23 is missing in E-YTH. (*D*) Violin plot visualizing the number of genes per cluster. No genes were detected in cluster 23 in E-YTH samples. (Red) E-YTH, (blue) E-YTH^mut^. (*E*) m^6^A counts per cluster. (*F*) Metagene analysis of individual cell clusters identified by single-cell sequencing. The m^6^A number surrounding the stop codon (position 0) is shown. m^6^A sites were obtained after eliminating background editing sites. Clusters 1, 18, 19, and 22 are shown, which represent different m^6^A distribution patterns.

We administered AAV containing *E-Yth* or *E-Yth*^*mut*^ into the hippocampi of 3-month-old mice (Fig. 3A) and optimized the protocol for dissociating the mouse hippocampus into single cells owing to their fragility and variability in shape and size (see Methods). We next FACS-sorted for EGFP to isolate cells successfully transduced with *E-Yth* or *E-Yth*^*mut*^. This selection process is crucial to exclude cells with insufficient E-YTH or E-YTH^mut^ expression, thereby preventing m^6^A false negatives that could lead to overestimations of m^6^A heterogeneity in RNA replicates. Following stringent EGFP selections, cells were then loaded into microwells (Rhapsody, BD Biosciences) for barcoding, leading to library generation and high-throughput sequencing (Fig. 3A; Supplemental Fig. S3A). Our evaluation confirmed the high quality and adequate read counts in the E-YTH and E-YTH^mut^ single-cell libraries (Supplemental Fig. S3B; Supplemental Table S8). We identified 11,561 cells with barcodes and UMIs in the E-YTH samples and 16,243 in the E-YTH^mut^ samples (Supplemental Table S8) and conducted clustering on the integrated E-YTH and E-YTH^mut^ single-cell data sets (Fig. 3B) using the uniform manifold approximation and projection (UMAP) dimension reduction method, revealing 28 clusters. Despite the absence of cluster 23 in our E-YTH data, all other clusters exhibited similar cell numbers between E-YTH and E-YTH^mut^ (Fig. 3C; Supplemental Fig. S3C). The absence of cluster 23 was unexpected, as the number of genes detected in all other clusters is very similar, with an average of 2800 for E-YTH and 2373 for E-YTH^mut^ (Fig. 3D). Also, few transcriptional changes were detected in E-YTH versus E-YTH^mut^ single-cell data (Supplemental Fig. S4A,B). Because clusters 21, 22, and 24–27 have fewer cell numbers than cluster 23 of E-YTH^mut^ (Supplemental Fig. S3C), the absence of cluster 23 in E-YTH cannot be attributed to not enough sequencing depth. Thus, the absence of cluster 23 seems E-YTH specific. Cluster 23, identified as neurons through automatic gene annotation (Supplemental Fig. S4C), was specifically enriched in CAMK2A (Supplemental Table S10), a gene expressed in a subgroup of excitatory neurons in the hippocampus and cortex, known for its role in long-term memory consolidation (Zhang et al. 2018; Yasuda et al. 2022). We next asked if we can validate our single-cell data and the absence of CAMK2A-expressing cells in E-YTH but not in E-YTH^mut^ hippocampi. Although our single-cell data did not reveal any CAMK2A-expressing cells following *E-Yth* injections, western blot analysis still detected a substantial, albeit reduced amount of CAMK2A proteins in E-YTH (Supplemental Fig. S4D). This discrepancy stems from our single-cell experiments, in which we FACS-sorted *E-Yth* transduced cells. In contrast, western blot analysis encompassed the entire hippocampus owing to material limitations, encompassing both transduced and nontransduced cells. To corroborate our findings, we employed pAAV-*Camk2a*-*mCherry* to visualize CAMK2A-expressing cells, identifiable by their red appearance. This virus was mixed with equal amounts of EGFP-expressing *E-Yth* or *E-Yth*^*mut*^ AAVs along with *Camk2a-mCherry* alone and subsequently injected into mouse hippocampi. When we coinjected *E-Yth*^*mut*^ and pAAV-*Camk2a*-*mCherry,* EGFP and mCherry signals overlapped, indicating the presence of CAMK2A-expressing cells. However, when coinjecting *E-Yth* and pAAV-*Camk2a*-*mCherry*, the overlap was reduced, suggesting fewer or no CAMK2A-expressing cells in our E-YTH single-cell data, and reinforces our single-cell findings, suggesting the importance of m^6^A containing transcripts in CAMK2A neurons (Supplemental Fig. S4E).

### Identification of m^6^A sites in single-cell clusters of the hippocampus

We identified C-to-U editing sites in our mouse hippocampus single-cell sequencing data and isolated m^6^A sites by filtering out bulk background C-to-U editing events detected in the WT, E-APOBEC1, and E-YTH^mut^ controls (see Methods). This process yielded 2,566,141 potential m^6^A sites. To address unequal background distributions in clusters, we also removed cluster-specific E-YTH^mut^ background for each cell in the E-YTH single-cell data, resulting in 923,249 of m^6^A sites in transcripts on a single-cell level (see Methods) (Supplemental Table S9). Cluster 22 exhibited the highest average number of m^6^A sites per cell (178), whereas cluster 18 displayed the lowest (18) (Fig. 3E; Supplemental Table S9). To validate our single-cell m^6^A data, we compared the data with m^6^A sites identified by m^6^A-RIP and our bulk E-YTH data (Supplemental Fig. S3D). Although we find m^6^A overlaps across all methods, more m^6^A sites were detected in the single-cell data owing to their enhanced sensitivity.

We then examined the m^6^A distribution near the TTS. Aggregating all clusters, we observed a m^6^A distribution near the TTS like bulk m^6^A sequencing data (Supplemental Fig. S5A). Although most m^6^A distribution patterns aligned with bulk m^6^A detection, cluster-specific variations were evident (Fig. 3F; Supplemental Fig. S5B). For instance, cluster 1 exhibited a common m^6^A distribution pattern with heightened enrichment post-TTS and a minor peak within 200 bp before the TTS, consistent with bulk m^6^A distribution (Fig. 3F; Supplemental Fig. S5A,B). In contrast, clusters like 22 and 5 lacked the pre-TTS enrichment peak, whereas cluster 19 showed the m^6^A peak at the TTS and cluster 18 upstream of the TTS (Fig. 3F; Supplemental Fig. S5B). These distinct m^6^A distribution patterns, often concealed in bulk data, may indicate regulatory or functional differences in m^6^A within specific cell types, emphasizing the benefits of single-cell resolution for modification detection.

### Identification of five major cell types in hippocampal m^6^A single-cell data

**Figure 4.**
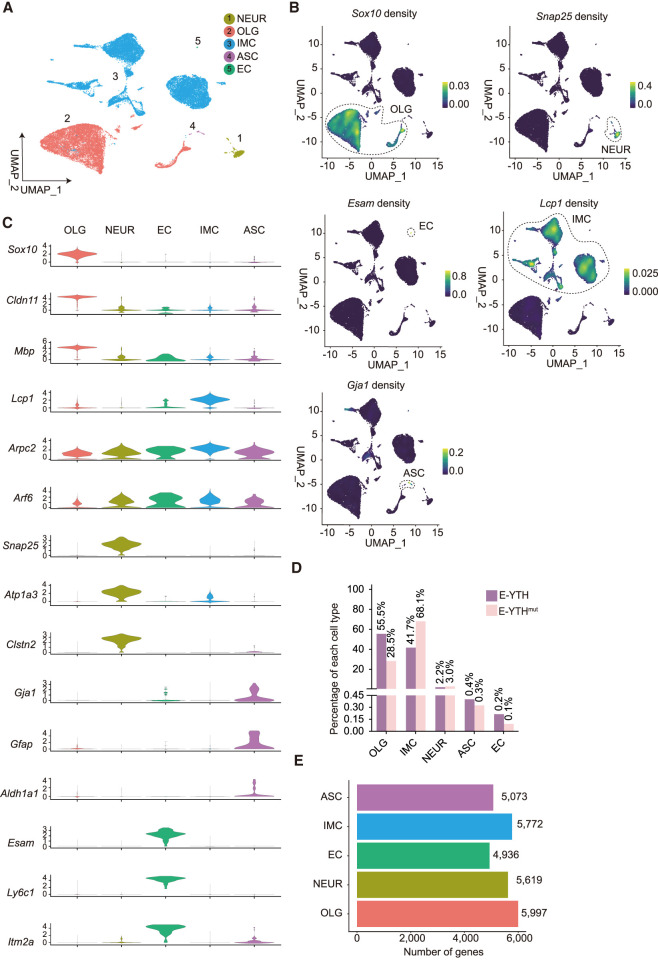
Identification of cell types at the single-cell level in the hippocampus. (*A*) UMAP with five main cell type populations were annotated and color coded based on cell type identifications. (NEUR) Neuronal cell lineage, (OLG) oligodendrocyte cell lineage, (IMC) immune cell lineage, (ASC) astrocyte cell lineage, (EC) endothelial cell lineage. (*B*) UMAP with expression levels of cell type–specific marker genes identifying all five major cell populations. Legend color represents RNA density. Circles were added to visualize grouped cell populations. OLGs have high expression of *Sox10*; NEURs have high expression of *Snap25*; ECs have high expression of *Esam*; IMCs have high expression of *Lcp1*; and ASCs have high expression of *Gja1*. (*C*) Violin plot showing the distribution of expression levels of well-known representative cell type–enriched marker genes across five cell types, 27,804 cells in total. (*D*) Percentage of each cell type. (*E*) Number of detected genes per cell type.

Using automatic annotation (see Methods) and validating with published work (Ximerakis et al. 2019), we identified cell type clusters based on specific gene markers (Fig. 4A–C; Supplemental Table S10). Clusters 2, 3, 5, 6, 7, 12, and 13 were classified as oligodendrocytes, with cluster 16 as oligodendrocyte precursor cells, confirmed by *Sox10* expression and all referred to as the oligodendrocyte cell lineage (OLG) (Ximerakis et al. 2019). Cluster 22 was designated as astrocytes (ASCs) owing to exclusive *Gja1* expression (Fig. 4B; Cid et al. 2021). Neuronal lineage cells (NEUs) were found in clusters 11 and 23 expressing *Snap25* (Ximerakis et al. 2019). Endothelial cells (ECs) were identified in cluster 26 expressing *Esam* (Ximerakis et al. 2019). Immune cell lineage (IMC) encompassed clusters 0, 1, 4, 8–10, 14–15, 17–21, 24–25, and 27 expressing *Lcp1*, subdivided into microglia (*Aif1* [also known as *Iba1*]), *Tmem119*, and *P2ry12* expression; clusters 1, 4, 14, 20) (Jurga et al. 2020), myeloid cells (*Cd74* expression; clusters 0, 9–10, 15, 17, 27) (Ximerakis et al. 2019), B cells (*Ly6d* expression; cluster 21, 25) (Blumberg et al. 1990; Ximerakis et al. 2019), and T cells (*Cd3e* expression; clusters 8, 18–19, 24) (Blumberg et al. 1990) based on marker expression (Fig. 4A–C; Ximerakis et al. 2019).

In total, we analyzed 27,804 cells, categorizing them into five main cell type lineages: NEUR, OLG, IMC, ASC, and EC (Fig. 4A). OLG cells dominated, followed by IMC, NEUR, ASC, and EC (Fig. 4D,E; Supplemental Fig. S6A; Supplemental Table S11). Notably, E-YTH^mut^ exhibited more IMC cells compared with E-YTH, without missing clusters in either group.

### Hippocampal m^6^A cell type characteristics

**Figure 5.**
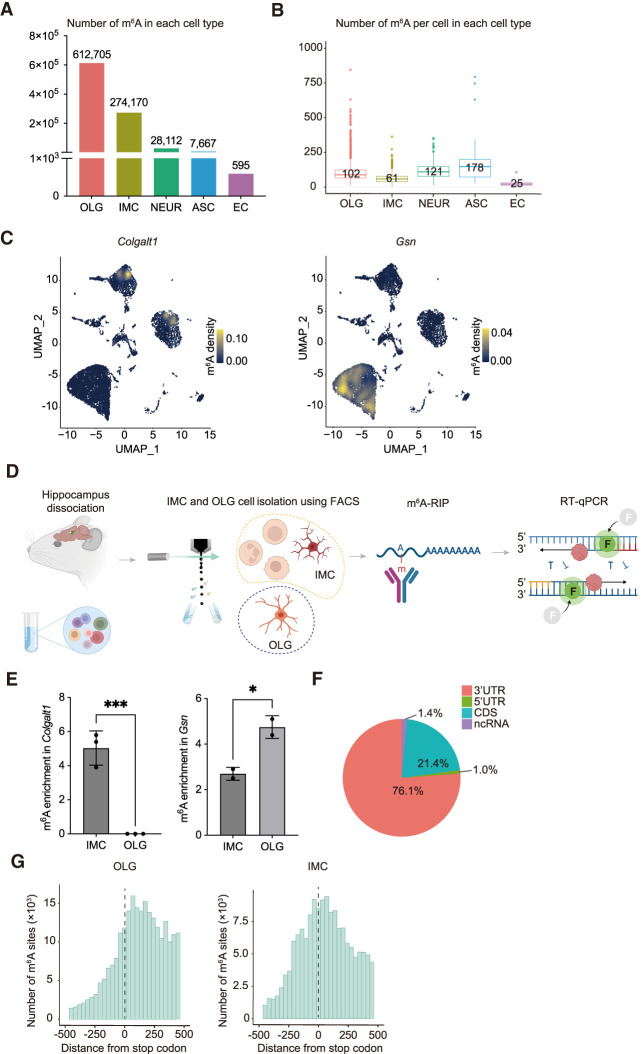
m^6^A single-cell distribution per mouse hippocampal cell type. (*A*) m^6^A counts per cell type. (OLG) oligodendrocyte cell lineage, (IMC) immune cell lineage, (NEUR) neuronal cell lineage, (ASC) astrocyte cell lineage, (EC) endothelial cell lineage. (*B*) m^6^A counts per cell for each cell type. Number reflects average. (*C*) UMAP illustrating the m^6^A density on RNA transcribed from one gene, per cell. Plots for genes *Colgalt1* and *Gsn* are shown. Legend color represents density of m^6^A on RNA transcribed. (*D*) Schematic diagram of approach confirming differential m^6^A RNA modifications in different cell populations. Relevant cell groups from hippocampi are isolated by FACS sorting, such as IMC versus OLG, followed by m^6^A RNA immunoprecipitation (m^6^A-RIP), reverse transcription, and qPCR (RT-qPCR). m^6^A-RIP enrichment represents relative m^6^A abundance in different cell populations. (*E*) m^6^A transcript enrichment quantifications in IMC and OLG populations following m^6^A-RIP and RT-qPCR versus input control samples. Transcript m^6^A enrichments represent m^6^A abundance in IMC and OLG cells. Both transcripts of *Colgalt1* and *Gsn* were detected in all input control samples. No *Colgalt1* transcripts were detected following m^6^A-RIP in OLG. Unpaired *t*-test (two-tailed) was used to test the difference between OLG compared with IMC. (***) *P* ≤ 0.001, (*) *P* ≤ 0.05. (*F*) m^6^A distribution within RNA. Data represent pooled single-cell data. (CDS) coding site, (ncRNA) noncoding RNA. (*G*) m^6^A distribution surrounding the stop codon (0 nt) identified by single-cell sequencing for OLG and IMC.

We investigated cell type–specific m^6^A characteristics, identifying the following average m^6^A site counts per cell: 612,705 m^6^A sites in OLG (5972 cells, 102 sites/cell), 274,170 m^6^A sites in IMC (4487 cells, 61 sites/cell), 28,112 m^6^A sites in NEUR (232 cells, 121 sites/cell), 7667 m^6^A sites in ASC (43 cells, 178 sites/cell), and 595 m^6^A sites in EC (23 cells, 25 sites/cell) (Fig. 5A,B; Supplemental Table S12). UMAP plots revealed differential m^6^A site expression for various genes (Fig. 5C).

To validate the differential m^6^A patterns in our single-cell data, we targeted the abundant IMC and OLG cell types. *Colgalt1* and *Gsn* were identified as genes with distinct m^6^A patterns in IMC and OLG (Fig. 5C). Isolating these cell groups from WT hippocampi, we conducted m^6^A-RIP followed by RT-qPCR to quantify *Colgalt1* and *Gsn* enrichment (Fig. 5D). *Colgalt1* displayed m^6^A sites in IMC but not OLG, whereas *Gsn* exhibited higher m^6^A enrichment in IMC than OLG (Fig. 5E). This m^6^A-RIP experiment validated our single-cell m^6^A findings, bolstering the reliability of our conclusions (Fig. 5C–E).

To determine if the m^6^A differential distribution could be explained by different levels of m^6^A regulatory enzymes, we also evaluated m^6^A regulatory enzymes at the single-cell level (Supplemental Fig. S6B). Although we observed a correlation between certain m^6^A methylases and increased m^6^A density, we also noted elevated levels of the m^6^A demethylase *Alkbh5* (Supplemental Fig. S6B). This implies that m^6^A regulation extends beyond the primary regulatory enzymes, suggesting a complexity in m^6^A regulation that surpasses current understanding, indicating tightly controlled, cell-specific mechanisms at play.

Although our pooled single-cell data confirmed previously reported m^6^A mRNA distribution patterns (Fig. 5F; Supplemental Fig. S5A; Supplemental Table S13), cell type–level analysis demonstrates specific m^6^A distribution patterns: OLG, NEUR, ASC, and EC exhibited a peak post-TTS, whereas IMC displayed an additional peak pre-TTS, indicating potential regulatory or functional differences between cell types (Fig. 5G; Supplemental Fig. S6C).

### Heterogeneous and homogeneous m^6^A sites in single cells

**Figure 6.**
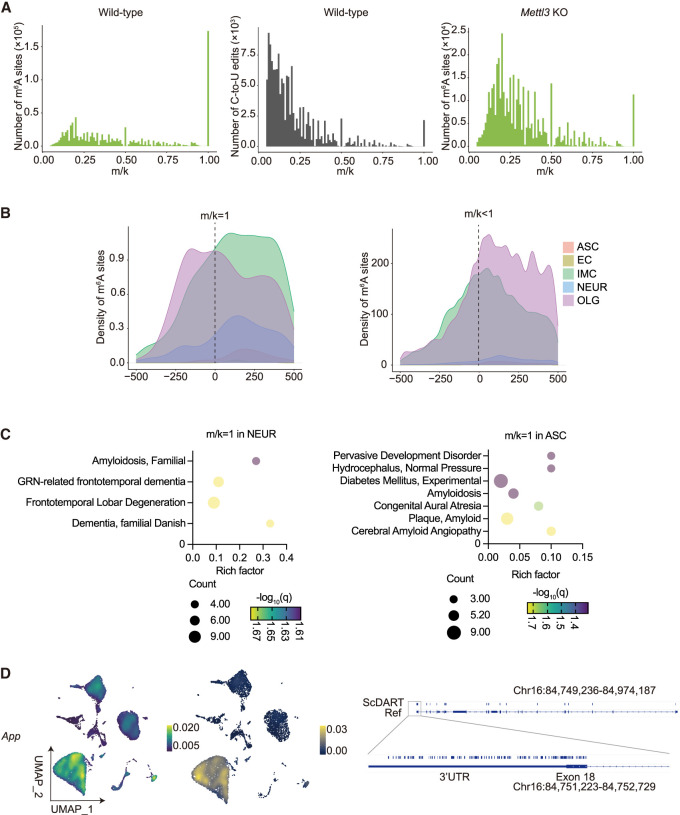
Heterogeneous and homogeneous m^6^A sites in single cells. (*A*) m^6^A site counts over mutation per read (m/k) ratio (*left*) and C-to-U edit E-YTH^mut^ background over mutation per read (m/k) ratio (*middle*) for WT mice. m^6^A site counts over m/k ratio for *Mettl3* knockout (KO) mice (*Mettl3*^−/−^*:Emx1-Cre*; *right*). Minimum threshold: 5%. (*B*) Normalized m^6^A scaled density 500 nt 5′ and 500 nt 3′ from stop codon (0 nt) for ASC, EC, IMC, NEUR, and OLG. (*Left*) m^6^A sites with m/k = 1 (homogenous m^6^A). (*Right*) m^6^A sites with m/k < 1 (heterogenous m^6^A). (ASC) astrocyte cell lineage, (EC) endothelial cell lineage, (IMC) immune cell lineage, (NEUR) neuronal cell lineage, (OLG) oligodendrocyte cell lineage. (*C*) Disease gene enrichment analyses (GO). GO terms with an adjusted *Q-*value < 0.05 and *P-*value < 0.05 are shown for NEUR and ASC. (*D*) Localization of m^6^A for gene. (*Left*) UMAP plot of single-cell expression for one gene. Legend color represents transcript density. (*Middle*) UMAP density plot of m^6^A on RNA transcribed from one gene, per cell. Legend color represents m^6^A density on RNA transcribed from one gene. (*Right*) IGV RefSeq gene annotation with editing sites representing adjacent m^6^A sites is shown. Last exon with 3′ UTR region is illustrated with higher magnitude.

We investigated m^6^A variations across clusters and cell types in our single-cell data by analyzing m/k ratios per gene per cell. Despite heterogenous m^6^A sites, many homogenous m^6^A sites (m/k = 1) were identified across all clusters and cell types (Fig. 6A; Supplemental Fig. S7). Comparing m/k distributions with E-YTH^mut^ controls supports m^6^A homogeneity (Fig. 6A; Supplemental Fig. S7). To exclude the possibility that the observed homogeneous m^6^A sites were not mouse-specific SNPs, we isolated m/k = 1 m^6^A sites per cell type, retaining only those that are present in other cells with a lower m/k ratio (m/k < 0.9) (Supplemental Table S14). To authenticate m^6^A sites and their stoichiometry, we generated *Mettl3* KO (*Mettl3*^−/−^*:Emx1-Cre)* mice to reduce m^6^A levels in hippocampi (Fig. 6A; Supplemental Fig. S8A–C, Supplemental Table S15). We next conducted m^6^A single-cell sequencing, as previously performed for WT mice (Supplemental Fig. S8D–F). In our WT m^6^A single-cell data, we could identify CAMK2A neurons in E-YTH^mut^ samples but not in E-YTH samples (Fig. 3C; Supplemental Fig. S4C–E). However, in our METTL3-depleted m^6^A single-cell data, we could identify CAMK2A neurons in both E-YTH^mut^ and in E-YTH samples (Supplemental Fig. S8G). The rescue of CAMK2A neurons in E-YTH samples following *Mettl3* depletion indicates that CAMK2A neurons are lost in E-YTH WT samples owing to the binding of the E-YTH construct to actual m^6^A sites, further corroborating our approach and CAMK2A findings. Furthermore, a considerable reduction in m^6^A sites was detected in *Mettl3* KO mice compared with those identified in WT hippocampi, thereby validating the authenticity of WT m^6^A sites (Fig. 6A; Supplemental Fig. S8H, Supplemental Table S15). This was further supported by the m/k ratio analysis, in which m^6^A sites with m/k = 1 in WT exhibited either no or reduced methylation (m/k = 0 or 0 < m/k < 1, respectively) in METTL3-depleted samples, corroborating the m^6^A homogeneity observed in single cells (Fig. 6A; Supplemental Fig. S8I).

Examining the distribution of conserved homogenous (m/k = 1) m^6^A sites, we observed distinct patterns compared with more heterogeneous (m/k < 1) sites (Fig. 6B). In OLG, homogenous m^6^A sites were enriched pre-TTS, with an additional peak at the TTS, whereas heterogeneous sites were mainly enriched shortly post-TTS. In IMC, heterogeneous m^6^A sites showed enrichment post-TTS, with a single major peak, whereas homogenous sites were distributed more evenly along the 3′ UTR, also occurring post-TTS (Fig. 6B). These differential distribution patterns suggest cell type–specific m^6^A regulation and potential functional implications.

To gain insights into the transcripts and pathways potentially regulated by homogeneous m^6^A sites, we conducted Gene Ontology (GO) analysis (Fig. 6C; Supplemental Fig. S9A). Our analysis revealed lineage-specific pathways, such as axon-related pathways for NEUR, oligodendrocyte-specific pathways for OLG, and postsynaptic pathways for ASC (Supplemental Fig. S9A). Additionally, disease pathways such as amyloidosis, dementia and degeneration for NEUR and hydrocephalus and amyloid plaque for ASC were identified (Fig. 6C; Supplemental Fig. S9B).

Focusing on genes with m^6^A sites at m/k = 1 in at least one cell, we observed m^6^A occurrences on transcripts encoding the m^6^A demethylase ALKBH5, suggesting a regulatory feedback mechanism (Supplemental Table S14). Moreover, m^6^A was found on *Fos* and *Jun* in OLG and IMC, with instances of multiple m^6^A sites on a single transcript, such as *Stat1* (Supplemental Table S14). We also detected m^6^A sites on *Smarcc2* (Supplemental Fig. S10), a regulator of chromatin structure implicated in neural stem cell proliferation and neuronal differentiation (Nguyen et al. 2018), hinting at a potential regulatory role of m^6^A via SMARCC2. Furthermore, several genes associated with Alzheimer's disease (AD) exhibited multiple cells with an m/k = 1 ratio, including *App*, *Apoe*, *Aplp1*, *Ctsb*, and *Itm2b* (Fig. 6D; Supplemental Fig. S10; Supplemental Table S14; Turner et al. 2003; Priller et al. 2006; Hook et al. 2023), suggesting potential m^6^A regulation. Additionally, other m^6^A transcripts with m/k = 1 are linked to diseases, such as *Mecp2* with Rett syndrome (Guy et al. 2007), *Syt11* with schizophrenia and Parkinson's disease (Inoue et al. 2007; Lill et al. 2012), *Lamp1* as a Lassa virus receptor (Enriquez et al. 2022), and *Brd2* with epilepsy (Supplemental Fig. S10; Supplemental Table S14; Pal et al. 2003), highlighting a broader impact of m6A modifications on disease-associated genes.

In summary, our single-cell data from the hippocampus unveiled various transcripts, cell clusters, and cell types likely regulated by m^6^A modifications. This comprehensive exploration of m^6^A dynamics within individual cells offers novel insights into the molecular underpinnings of hippocampal physiology, setting the stage for future investigations into the dynamic landscape of m^6^A RNA methylation in the brain.

## Discussion

In this study, we optimized the DART-seq system (Meyer 2019) to identify m^6^A sites in single-cell data from the mouse hippocampus. Initially, we generated *E-Yth* and *Yth-E* constructs and their controls, testing their efficacy in detecting m^6^A sites in RNA from HEK293T cells. Our findings align with previous m^6^A studies, validated through functional investigations (Meyer 2019). The *E-Yth* construct showed superior performance, improving the detection of *E-Yth*-transfected cells and correlating with APOBEC1-YTH presence. Using the *E-Yth* construct, we achieved a distinct m^6^A distribution pattern by excluding cells with low E-YTH expression, reducing background noise. Subsequently, we packaged the *E-Yth* construct and its *E-Yth*^*mut*^ control into AAVs and injected them into mouse hippocampi, isolating transduced cells for bulk and single-cell sequencing analysis to identify m^6^A sites.

We identified 923,249 m^6^A sites within transcripts at single-cell resolution in the mouse hippocampus. CAMK2A-expressing cells were absent after APOBEC1-YTH treatment, suggesting a potential interference with essential m^6^A transcripts in these neurons. This interference may be owing to C-to-U near m^6^A sites, which are mutations, introduced by APOBEC1-YTH, potentially affecting m^6^A-containing transcripts or competing with m^6^A reader proteins like YTHDF2. Although APOBEC1-YTH have been previously reported not to interfere with transcriptional changes or cell viability (Meyer 2019), occasionally, such mutations could result in changes, which might explain the E-YTH-specific absence of CAMK2A neurons. The absence of CAMK2A neurons in E-YTH data but not in E-YTH^mut^ controls underscores the importance of m^6^A in these cells, which has been observed previously (Engel et al. 2018; Zhang et al. 2018; Xu et al. 2022; Yasuda et al. 2022).

Our study also reveals homogeneity of m^6^A in individual cells, contrasting with previous reports of heterogeneity (Tegowski et al. 2022). Differential distribution patterns near TTS between homogenous and heterogeneous m^6^A sites suggest regulatory and functional differences. Transcripts with high m/k ratios, like *App* and *Smarcc2*, may be particularly sensitive to m^6^A regulation. We discovered many such m^6^A transcripts in specific cell types associated with brain diseases, indicating potential implications for conditions like AD. Because an earlier study highlighted a decline in m^6^A levels with age and in AD (Castro-Hernández et al. 2023), our findings suggest that specific m^6^A transcripts like *App*, *Apoe*, *Aplp1*, *Ctsb*, and *Itm2b*, alongside m^6^A-modulated transcripts in CAMK2A neurons of the hippocampus, not only exhibit sensitivity to m^6^A regulation but are also likely implicated in the aging process and the pathology of AD. By uncovering novel insights into the hippocampal m^6^A transcriptome at the single-cell level, our work paves the way for future therapeutic targets and studies on m^6^A dynamics in brain health and disease.

## Methods

### Cell lines

HEK293T cells were purchased from the Beijing Xiehe Cell Bank and cultured at 37°C with 5% CO_2_ in Dulbecco's Modified Eagle Medium (DMEM, Gibco C11965500BT) containing 10% FBS (VisTech SE100-B) and 1% penicillin/streptomycin. Cells were passaged for fewer than 20 times and have been regularly tested for mycoplasma.

### Mouse strains

Animals were maintained and handled following the guidelines of the Chinese Institute for Brain Research (CIBR). All experimental methods were approved and adhered to the regulations of the Welfare and Ethics Review Committee for Laboratory Animals. WT C57BL/6J mice were made available through CIBR's animal facility. *Mettl3*-floxed (*Mettl3*^*f/f*^) mice from GemPharmatech were bred with *Emx1-Cre* mice (JAX stock 005628) (Gorski et al. 2002) to produce homozygous *Mettl3* KO (*Mettl3*^−/−^*:Emx1-Cre*) mice. The genotyping primers for *Mettl3*^*f/f*^ facilitate detection of the 5′ flox region (forward primer: ATAACCCTGGCTGTCCCG; reversed primer: ATAACCCTGGCTGTCCCG) and the 3′ flox region (forward primer: CCTTTGGAATGGCTACTGC; reversed primer: ATCAGAAAGCCCATCCTCA). Adult WT and homozygous *Mettl3* KO adult mice, aged 8–12 weeks, were utilized for single-cell RNA sequencing. The mice were housed in a 12:12 light–dark cycle, maintained under controlled climate conditions, provided with enrichment environments, and had ad libitum access to sterile food and water.

### Vector cloning and AAVs

An Adeno-associated plasmid pAAV-*Cag-Egfp-Wpre-Sv40* (gift from Minmin Luo) was used as the backbone to generate the viral expression constructs pAAV-*Cag-Apobec1-Yth-Egfp* (*Yth-E*, Addgene 209322), pAAV-*Cag*-*Apobec1*-*Yth*^*mut*^-*Egfp* (*Yth^mut^-E*, Addgene 209323), pAAV-*Cag-Apobec1*-*Egfp* (Addgene 209324), pAAV-*Cag*-*Egfp*-*Apobec1*-*Yth* (*E-Yth*, Addgene 209319), pAAV-*Cag-Egfp-Apobec1*-*Yth*^*mut*^ (*E*-*Yth*^*mut*^, Addgene 209320), and pAAV*-Cag-Egfp-Apobec1* (Addgene 209321). To clone these vectors, the cassette pCMV-*Apobec1-Yth* (Addgene 131636) and pCMV-*Apobec1-Yth*^*mut*^ (Addgene 131637) were inserted into the backbone of pAAV-*Cag-gfp*-*Wpre-Sv40* using in-fusion cloning. pAAV-*Camk2a*-*mCherry* was also used (gift from Fei Zhao). The plasmid to be packaged was cotransfected into HEK293T cells with a rep/cap-containing plasmid *pUCmini-iCAP-PHP.eB* (Addgene 103005) and the helper plasmid *pAdDeltaF6* (Addgene 112867), in the presence of polyethyenimine. After 72 h, the AAV virus was harvested, purified by chloroform, titrated, and quantified by qPCR (Negrini et al. 2020). AAV stereotaxic injections were performed targeting the hippocampus of adult mice, with X/P 1.94 mm, M/L 1.5 mm from the bregma point, and 2 mm depth for C57BL/6J mice. Because of the reduced brain volume observed in *Mettl3* KO mice, a depth of 0.7 mm was applied in these mice, followed by an additional injection into the retro-bulbar sinus.

### Immunostaining and microscopy

HEK293T cells were seeded on a 35-mm-diameter dish (WPI's FluoroDish FD35-100); 5 µg plasmids were transfected; and images were taken after 24 h. Cells were fixed with 4% paraformaldehyde for 10 min and washed with PBS followed by 15 min permeabilization with 0.1% Triton X-100. Following PBS washes, samples were blocked for 1 h at RT in PBS with 1% BSA and incubated with HA antibody (Alexa Fluor 555, Invitrogen 26183-A555). After PBS washes and 0.1% DAPI staining, fluorescent images were captured using the Zeiss inverted confocal microscope (ZEN blue software; DAPI: excitation 365, BS FT 395, emission BP 445/50; GFP: excitation BP 470/40, BS FT 495, emission BP 525/50; CY3: excitation BP 545/25, BS FT 570, emission BP 605/70).

Figure 2B was imaged with the Olympus VS120 virtual slide microscope (OlyVIA software; DAPI: excitation 365/10 nm, emission 440/40 nm; GFP: excitation 472/30 nm, emission 520/35 nm). Supplemental Figure S4 was captured using the Olympus VS200 virtual slide microscope (OlyVIA software; Cy3: excitation 555/20 nm, emission 595/33 nm; GFP: excitation 480/30 nm; emission 519/26 nm; DAPI: excitation 395/25 nm, emission 434/32 nm).

### Bulk m^6^A sequencing in HEK293T cells

HEK293T cells were cultured and processed independently of each other to generate independent biological replicates. Three independent replicates were processed for *Yth-E-* and *Yth^mut^-E*-expressing cells. Twenty-four hours after plasmid transfections, cells were rinsed with DPBS and digested using 0.5% trypsin-EDTA. Cells were pelleted, washed with DPBS, and filtered through a 40 µm cell filter prior to FACS analysis. Cells were loaded onto a custom FACS ARIA III flow sorter (BD Biosciences) equipped with a 100 µm nozzle. Particles smaller than cells (dots) were eliminated using the forward-scatter (FSC-PMT-A) versus side-scatter (SSC-A). Cell-sized particles were gated (box). Plots of height versus width in the forward-scatter and side-scatter channels were used to exclude aggregates of two or more cells. Live cells were selected by gating the non-DAPI signal (405 nm laser, violet DAPI). GFP cells from C57BL/6J mice were isolated by green excitation light (488 nm laser, green FITC). Given the reduced hippocampal volume in *Mettl3* KO mice, no gating was applied to obtain enough *Mettl3* KO hippocampal cells. Total RNA was isolated with the micro total RNA isolation kit (Invitrogen AM1931) according to the manufacturer's instructions. After treatment with DNase I (Tiangen RT411) for 15 min at room temperature, sequencing libraries were generated from 1–10 ng of total RNA from each replicate using the single-cell full-length mRNA kit (Vazyme N712) and TruePrep DNA library prep kit V2 (Vazyme TD502), according to the manufacturer's instructions. Quality control was performed using the fragment analyzer systems capillary arrays (AATI, FA12) and quantified with a Qubit 1 × dsDNA HS kit (Invitrogen Q33231); 150 bp paired-end sequencing was performed on a NovaSeq 6000 (Illlumina) using a S4 flow cell.

### Identification of m^6^A sites in bulk RNA sequencing data

Low-quality bases (<Q20) and adaptor sequences were trimmed using Trimmomatic (0.39) (Bolger et al. 2014), and reads with fewer than 36 nucleotides were subsequently discarded. The cleaned reads were aligned to the mm39 reference genome using BWA-MEM (0.7.17) (Li and Durbin 2009). Duplicate reads were identified using the MarkDuplicates tool from Picard (https://broadinstitute.github.io/picard/). Subsequently, the CLIP tool kit (CTK) was employed to collapse PCR duplicates and to identify C-to-U editing events, following default parameters (Shah et al. 2017).

To identify C-to-U and m^6^A sites, we used an approach as described by Meyer (2019), using the following filters: (1) Only C-to-T mutations with a false-discovery rate (FDR) of less than 0.01 were kept for any downstream analyses; (2) only C-to-T mutations with two or more editing events (m ≥ 2) and a coverage of at least 10 (k ≥ 10) were considered for downstream analyses; (3) C-to-T mutations with a ratio of m/k (number of reads with a C-to-T mutation/total reads per site) >5% were kept for downstream analyses; (4) C-to-T mutations sites that were found in the single-nucleotide polymorphism (SNP) databases Mouse Genome Project (mgp_REL2021_snps) and Genome Reference Consortium Mouse Build 39 (GCA_000001635.9) were discarded; and (5) only C-to-T mutation sites that were found in at least two out of three replicates were considered for downstream analyses.

To identify m^6^A sites in the C-to-T-converted APOBEC1-YTH sequencing data, C-to-T conversion sites that were also present in the WT and APOBEC1 overexpression background control were removed. Only C-to-T conversion sites in the APOBEC1-YTH sequencing data that occurred at least 1.5 times more frequently than in the APOBEC1-YTH^mut^ negative control sequencing data were retained. By implementing these criteria, false positives were eliminated, resulting in a set of stringent C-to-T conversion sites in the APOBEC1-YTH data sets. These remaining C-to-T conversion sites in the APOBEC1-YTH data were identified as m^6^A sites.

### Analyses and plotting

Biorender (https://www.biorender.com/) was used for some illustrations, as well as the Integrative Genomics Viewer (IGV) (Robinson et al. 2011) and GraphPad Prism (version 9.5.1; RRID:SCR_002798). All experiments were carried out with three technical and biological replicates, indicated by n. Statistical analyses and plots were performed using R (version 3.6.2) (R Core Team 2024). The VennDiagram package was used for Venn diagrams, Seurat (Stuart et al. 2019) for UMAP plots, and ggplot2(version 3.3.5) (Wickham 2016) for the rest of the plots. metaPlotR (Olarerin-George and Jaffrey 2017) was used to generate m^6^A metagene plots, such as histograms along simplified transcript models, of the C-to-U conversion (Olarerin-George and Jaffrey 2017). When multiple transcript isoforms could potentially contain the C-to-U site, the longest isoform was chosen.

### Mass spectrometry

Analysis of global levels of A and m^6^A was performed on a TSQ Altis triple quadrupole mass spectrometer (Thermo Fisher Scientific) coupled to a Vanquish flex UHPLC system (Thermo Fisher Scientific) fitted with an acquity UPLC HSS T3 column (2.1 × 100 mm, 1.8 μm particle size, Waters). The mobile phase consisted of 0.5% aqueous formic acid (solvent A) and 0.5% formic acid in acetonitrile (solvent B) at a flow rate of 300 µL/min. Calibration curves were generated using serial dilutions of synthetic standards for adenosine (A; Sigma-Aldrich) and *N*^6^-methyl-2′-adenosine (m^6^A; Sellechchem). The mass spectrometer was set in a positive ion mode and operated in selective reaction monitoring. The precursor ions of A (m/z 268.1) and m^6^A (m/z 282.1) were fragmented, and the product ions of A (m/z 136.1) and m^6^A (m/z 150.1) were monitored. The EIC of the base fragment was used for quantification. Accurate mass of the corresponding base fragment was extracted using the XCalibur qual browser and XCalibur quan browser software (Thermo Fisher Scientific) and used for quantification. m^6^A presentage was calculated according to the following equation: m^6^A(%) = 100 × m^6^A/[A]. Differences in m^6^A percentage abundance were considered significant when *P* ≤ 0.05.

### m^6^A RNA immunoprecipitation

Total RNA was extracted from the hippocampus of adult mice (C57BL/6J) using TRIzol (Thermo Fisher Scientific 15596018) reagent. After removing genomic DNA, a RNeasy mini kit (Qiagen 74106) was used for RNA purification, resulting in ∼20 μg of total RNA per mouse. The integrity of RNA was assessed using the fragment analyzer system capillary arrays (AATI F12), whereas the concentration and purity were determined using a spectrophotometer (Thermo NanoDrop one). For RNA fragmentation, the samples were incubated for 4 min at 94°C in a fragmentation buffer (containing 10 mM ZnCl_2_, 10 mM Tris-HCl at pH 7), followed by standard isopropanol precipitation. For m^6^A-RIP, an existing protocol was adjusted (Dominissini et al. 2012). Protein A Dynabeads (Invitrogen 10001D) were washed three times with IP buffer (containing 150 mM NaCl, 0.1% NP-40, 10 mM Tris-HCl at pH7.4 and 6 µg/µL BSA) and incubated with rotation in IP buffer for 2 h at 4°C. Each RNA sample was divided to obtain an input control sample (10%), and the 90% was incubated with an anti-m^6^A antibody (Synaptic Systems 202011, 2.5 μg). The RNA–antibody mixture was incubated for 2 h at 4°C with rotation. The magnetic beads were then conjugated with the antibody–RNA solution, which selectively captures RNA fragments containing m^6^A modifications. After three washes with IP buffer, the antibody-captured RNA was eluted for 1 h at 4°C with shaking rotation using elution buffer (containing IP buffer and 6.6 mM m^6^A) and was then concentrated by isopropanol precipitation. To generate RNA-seq libraries for input control and m^6^A-RIP pulldown samples, the RNA was processed using the SMARTer stranded total RNA-seq kit v2 (Takara 634411). The fragment length of the libraries was verified using the fragment analyzer 12 (AATI). Paired-end 150 bp reads were sequenced with the NovaSeq 6000 platform (Illumina).

### m^6^A-RIP sequencing data analysis

rRNAs were removed using the mouse rRNA reference (GCF_000001635.27_GRCm39_rna_from_genomic.fna) from NCBI. Adapters were eliminated with cutadapt (version 2.8) (Martin 2011). The first 10 5′ nucleotides were eliminated from the ends owing to the presence of potentially low-quality nucleotides. PCR duplicates were removed from the aligned data sets. Mapping and alignment was done by HISAT2 (version 2.2.1) (Kim et al. 2019), followed by peak calling using MACS2 (version 2.2.6) (Gaspar 2018).

### Hippocampus dissociation

Two weeks after AAV brain stereotaxic injections, mice were anesthetized and then perfused with Dulbecco's phosphate buffer saline (MacGene CC010). Hippocampi were extracted in cold DPBS solution containing calcium, magnesium, and glucose and were dissociated into single cells using the adult brain dissociation kit (Miltenyi Biotex 130-107-677), under the following conditions: (1) ∼25 mg of adult hippocampi was used as starting material per sample; (2) the MACS program >100 mg:37°C_ABDK_01 was chosen; (3) debris was removed following the manufacturer's manual; (4) 10 mL PB buffer was used to suspend cells with cold 1× red blood cell removal solution; and (5) PB buffer was used for FACS sorting.

### Single-cell m^6^A sequencing

Eight hippocampi from adult mice were pooled for each sample to ensure an adequate number of cells. E-YTH and E-YTH^mut^ EGFP-positive cells were isolated through FACS sorting and converted into cDNA libraries. Thirty thousand cells per sample were loaded into a Rhapsody cartridge (BD Biosciences 633733) and processed following the manufacturer's instructions. Single-cell RNA sequencing libraries were generated using the Rhapsody WTA kit (BD Biosciences 633801). The Rhapsody platform by BD allows cells to settle naturally on a chip for gentle capture without disruption. After pooling the libraries, sequencing was performed using NovaSeq 6000 (Illumina).

### Single-cell sequencing gene expression analysis

The Rhapsody docker image (BD Biosciences) was used to perform barcode processing and single-cell gene-UMI counting, following manufacturer's instructions (BD Rhapsody sequence analysis setup). A digital expression matrix was obtained for each experiment with default parameters and was mapped to the mouse reference genome mm39. Matrices containing RSEC-corrected molecules per bioproduct per cell numbers were loaded into Seurat (version 3.1.5) (Butler et al. 2018). Low-quality cells with fewer than 500 and more than 6000 detected genes and cells with a high mitochondrial content (>10%), indicative of poor cell quality, were excluded. In *Mettl3* KO mice, given that *Emx1-Cre* expression is not ubiquitous (Gorski et al. 2002), it is possible that METTL3 may persist in a subset of *Mettl3* (*Mettl3*^−/−^*:Emx1-Cre*) KO cells. To ensure the removal of data from cells retaining METTL3 expression, any cells with *Mettl3* transcript counts of one or more were filtered out from all subsequent analyses in the *Mettl3* KO samples. The data from each individual single-cell sample were log-normalized. The top 2000 more variable genes within each sample were identified using the FindVariableFeatures function in Seurat and were used as integration anchors for the integration of E-YTH and E-YTH^mut^ samples. This was done using the FindIntegrationAnchors and IntegrateData functions in Seurat. Integrated data were subsequently scaled, and principal component analysis (PCA) was performed to reduce the dimensionality of the data. The top 30 and 21 principal components were used to identify cell populations for WT and *Mettl3* KO mice, respectively, using the FindNeighbors and FindClusters functions. The resolution parameter in FindClusters function was set to 0.8. UMAP was subsequently applied to visualize the clustered cells in a two-dimensional space.

To determine cluster-specific marker genes, the FindConservedMarkers function in Seurat was employed, comparing each cluster against all other clusters within the integrated data set. For each run of this function, only genes detected in at least 10% of the cells in either of the two populations were considered for analysis. Genes with a log-fold change greater than 0.3 were considered as cluster-specific markers. To help annotate the identified clusters, we followed two automatic cell type annotation approaches. First, we used the scMCA function in the scMCA package (0.2.0) (Sun et al. 2019). This function assigns mouse cell types to cell clusters based on expression profiles. Second, we used SciBet (1.0) cell type classifier with the *Tabula Muris* brain nonmyeloid model (Li et al. 2020).

Seurat was used for UMAP plots (Hao et al. 2021) and ggplot2 for the rest of the plots (Wickham 2016). UMAP plots were generated with Nebulosa, using the plot_density function (Alquicira-Hernandez and Powell 2021).

### Differential gene expression analysis

Gene-level read counts were obtained from the aligned files using featureCounts (2.0.0) (Liao et al. 2014). Differential expression analysis between E-YTH and E-YTH^mut^ cells was performed using DESeq2 (Love et al. 2014). After filtering out genes with low expression levels (number of reads <10) and adjusted *P-*value > 0.1, volcano plots were plotted. Adjusted *P-*value < 0.05 and a Log_2_FC > 1 and Log_2_FC < –1 are considered significant. Genes were identified as differentially expressed between the two conditions with a fold change of Log_2_FC > 1 and Log_2_FC < –1 (adjusted *P-*value < 0.05).

### Western blot

Hippocampi were homogenized in 500 µL of RIPA medium lysis buffer (Beyotime P0013E-2) in the presence of protease inhibitor cocktail (Roche 11836170001). Samples were run on 10% PAGE gels (Vazyme E303-01) and transferred onto activated PVDF transfer membranes (Immobilon IPVH00010). Membranes were washed with TBST (10 mM Tris-HCl at pH 8.0, 150 mM NaCl, 0.05% Tween 20), followed by blocking in 5% nonfat dried milk and incubation with antibodies such as CAMK2A (Thermo Fisher Scientific MA1-048) and GAPDH (Abcam ab9485). After TBST washes, membranes were incubated with HRP-conjugated secondary antibodies (Abcam Ab205718 & Ab205719) and washed, and proteins were visualized with a HRP substrate peroxide solution and luminol reagent (Immobilon WBKL5S0500). iBright 1500 (Invitrogen) was used to quantify protein presence (background corrected volume [Local Bg. Corr. Vol.]) versus GAPDH.

### Identification of m^6^A sites in single-cell sequencing data

The software Bullseye was used to identify m^6^A sites in single-cell sequencing data (Tegowski et al. 2022). We applied the same conditions for single-cell analysis as we did for bulk data, except for the following.

We excluded any C-to-U editing sites identified in the APOBEC1 and WT bulk sequencing analysis from the E-YTH single-cell data. Only sites showing a 1.5-fold increase over bulk E-YTH^mut^ controls were retained. Given that C-to-U background edits are not evenly distributed across all clusters, it was crucial to eliminate the E-YTH^mut^ cluster-specific background. We computed the average C-to-U editing events in E-YTH^mut^ per cluster. Although the ideal scenario would involve removing background at the single-cell level, this is unfeasible owing to the uniqueness of each cell. Consequently, we calculated the average C-to-U editing events in E-YTH^mut^ per cluster. Subsequently, for each cell in our E-YTH single-cell data, we subtracted the corresponding cluster-specific background average. This approach was vital for accurately capturing cluster-specific m^6^A characteristics.

### Comparison of bulk, single-cell, and m^6^A-RIP data sets

The m^6^A overlap among single-cell RNA-seq, bulk RNA-seq, and m^6^A-RIP was determined by identifying the intersection of genes in which a m^6^A position (or region for m^6^A-RIP) was identified.

### Confirmation of differential m^6^A methylation

Four WT mouse hippocampi were isolated and dissociated into single cells. OLG and IMC cells were isolated by FACS sorting using the oligodendrocyte marker O1 monoclonal antibody (O1), eFluor 660 (eBioscience 50-6506-80), the CX3CR1 monoclonal antibody (2A9-1), and Alexa Fluor 488 (eBioscience 53-6099-42), respectively. Following RNA isolation, m^6^A-RIP, and reverse transcription, qPCR was performed with the following primers: *Colgalt1* (F:AAGAACTCAGATGTGCTCCAG; R:CTATAGTCCCAGGCAAGCAC) and *Gsn* (F:CATCACAGTCGTTAGGCAGG; R:TGATGGCTTTGGTCCTTACTC). m^6^A-RIP versus matching input control samples were calculated.

### Identification of homogenous m^6^A sites in single-cell sequencing data

To identify homogenous m^6^A sites, the m/k ratio was first calculated, as described above for bulk m^6^A sites. To identify truly homogenous m^6^A sites (m/k = 1) in the single-cell data, we excluded the possibility that any homogenous (m/k = 1) sites might still represent mouse-specific SNPs. Thus, we identified all m/k = 1 m^6^A sites per cell for all cells and then only kept those m/k = 1 m^6^A sites that occurred in at least five other cells with a lower m/k ratio (m/k < 0.9) and a minimum read overage of 10 per cell.

### GO analysis

Prior to GO analysis, conserved homogenous m^6^A sites were identified. Such sites for each cell types consist of m/k = 1 sites identified in our merged single-cell RNA sequencing data that occur with an m/k < 0.9 ratio in at least five cells of other cell types. For each cell type, GO biological process, molecular function, and cellular component enrichment analyses were carried out on the set of genes in which these conserved homogenous sites occur, using cluster Profiler (version 3.14.3) (Yu et al. 2012). Terms with an adjusted *Q*-value < 0.05 and *P-*value < 0.05 were considered statistically significant. The top five GO terms were plotted, in addition to five selected terms that are statistically significant. Additionally, disease enrichment analysis was conducted on the human orthologue genes corresponding to the genes with conserved homogenous sites, using DOSE (version 3.12.0) and DisGeNET (Yu et al. 2015; Piñero et al. 2017). Terms with an adjusted *Q-*value < 0.05 and *P-*value < 0.05 were considered statistically significant and plotted. Prism (V9.5.1) was used to plot any GO terms. The Rich factor is the ratio of gene numbers with m/k = 1 m^6^A in a pathway term to all gene numbers annotated in this pathway term.

## Data access

All raw and processed sequencing data generated in this study have been submitted to the NCBI Gene Expression Omnibus (GEO; https://www.ncbi.nlm.nih.gov/geo/) under accession number GSE240863. All plasmids generated for this study can be obtained through Addgene (Addgene 209319 to 209324; https://www.addgene.org/Magdalena_Koziol). Codes used for this work are available through GitHub (https://github.com/KoziolLaboratory/sc-m6a-hippocampus) and as Supplemental Code. Single-cell RNA and m^6^A density UMAP visualizations can be accessed via our interactive website (https://scm6a.cibr.ac.cn/). This website is accompanied by a navigation guide and summary. Within the scm^6^A-seq menu, all Gene_Density and m6A_Density UMAP plots generated can be accessed. Gene names can be searched (the first letter needs to be capitalized, followed by enter key) within the Gene_Density and m6A_Density sections. The corresponding Gene_Density or m6A_Density UMAP plots will be displayed for the gene of interest and can be interpreted through legends provided.

## Supplementary Material

Supplement 1

Supplement 2

Supplement 3

Supplement 4

Supplement 5

Supplement 6

Supplement 7

Supplement 8

Supplement 9

Supplement 10

Supplement 11

Supplement 12

Supplement 13

Supplement 14

Supplement 15

Supplement 16

Supplement 17

Supplement 18

Supplement 19

Supplement 20

Supplement 21

Supplement 22

Supplement 23

Supplement 24

Supplement 25

Supplement 26
