# Supplementary material for: Single-cell discovery of m6A RNA modifications in the hippocampus
Source: Genome Res. 2024 Jun;34(6):822–36. doi: 10.1101/gr.278424.123 (PMC11293556; doi:10.1101/gr.278424.123)
Supplement: Supplement 1 [file Supplemental_Fig_S1.docx]

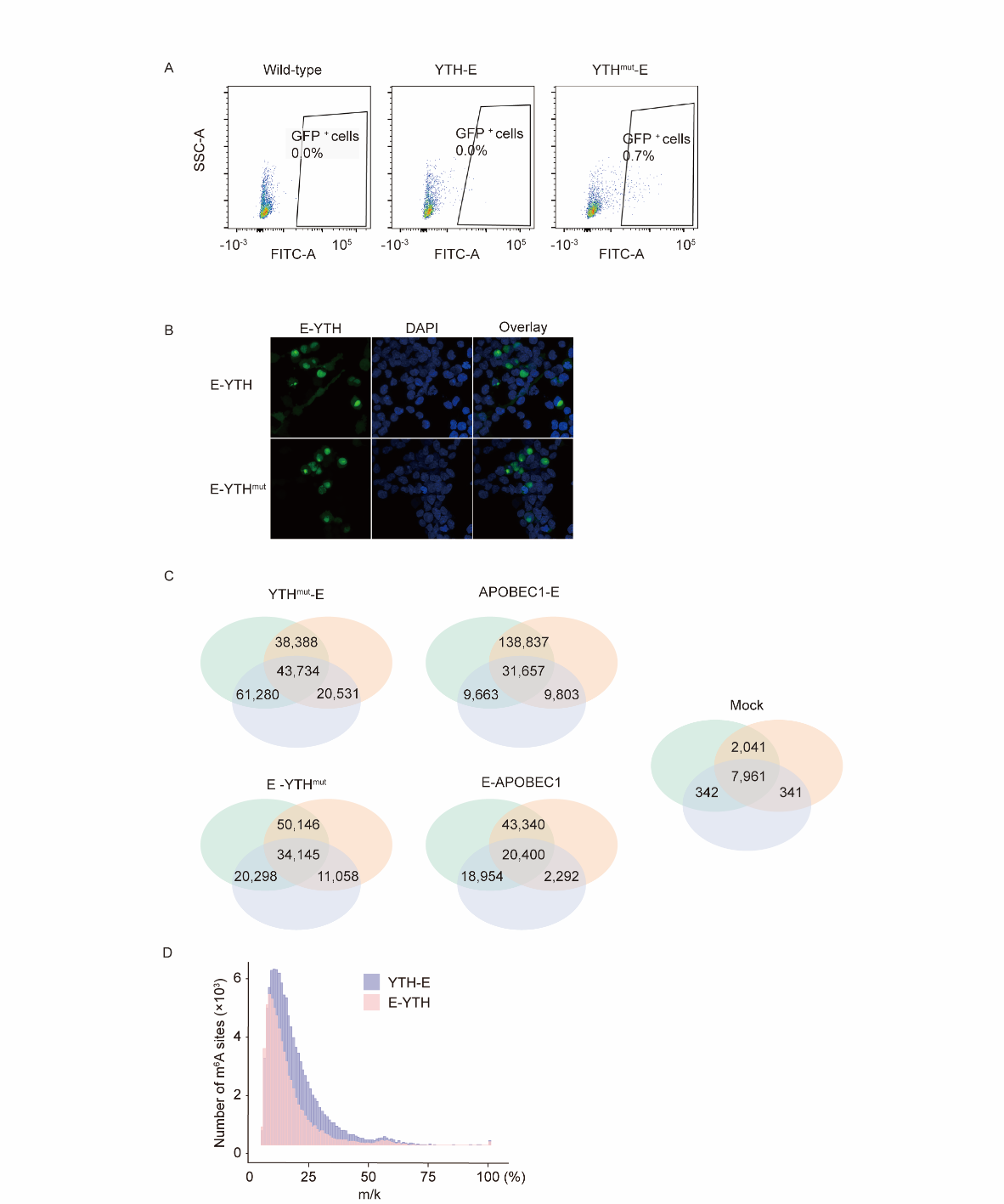


**Supplemental Fig S1. Improved detection of m^6^A in HEK293T cells with bulk RNA-seq.**

(A) Detection of EGFP positive cells in YTH-E, YTH^mut^-E and wild type cells from mouse hippocampi. FACS gating is shown. The value indicates percentage of EGFP positive cells divided by total cells, excluding debris (P1 gate).

(B) Confocal images of HEK293T cells transfected with E-YTH and E-YTH^mut^. Representative images are shown, 24 hrs after transfection. Scale bar, 20 μm.

(C) Number of C-to-U editing events identified in each bulk HEK293T cell replicate for YTH^mut^-E, E-YTH^mut^, APOBEC1-E, E-APOBEC1 and mock. Except for mock, the data was obtained following transfection and EGFP FACS sorting. n=3, Rep: separately cultured replicate.

(D) Histogram for YTH-E and E-YTH showing m^6^A counts over mutation per read (m/k) ratio. For both samples, background C-to-U editing sites identified in the corresponding control groups were removed. A minimum threshold of 5% was applied.
