# Supplementary material for: Single-cell discovery of m6A RNA modifications in the hippocampus
Source: Genome Res. 2024 Jun;34(6):822–36. doi: 10.1101/gr.278424.123 (PMC11293556; doi:10.1101/gr.278424.123)
Supplement: Supplement 2 [file Supplemental_Fig_S2.docx]

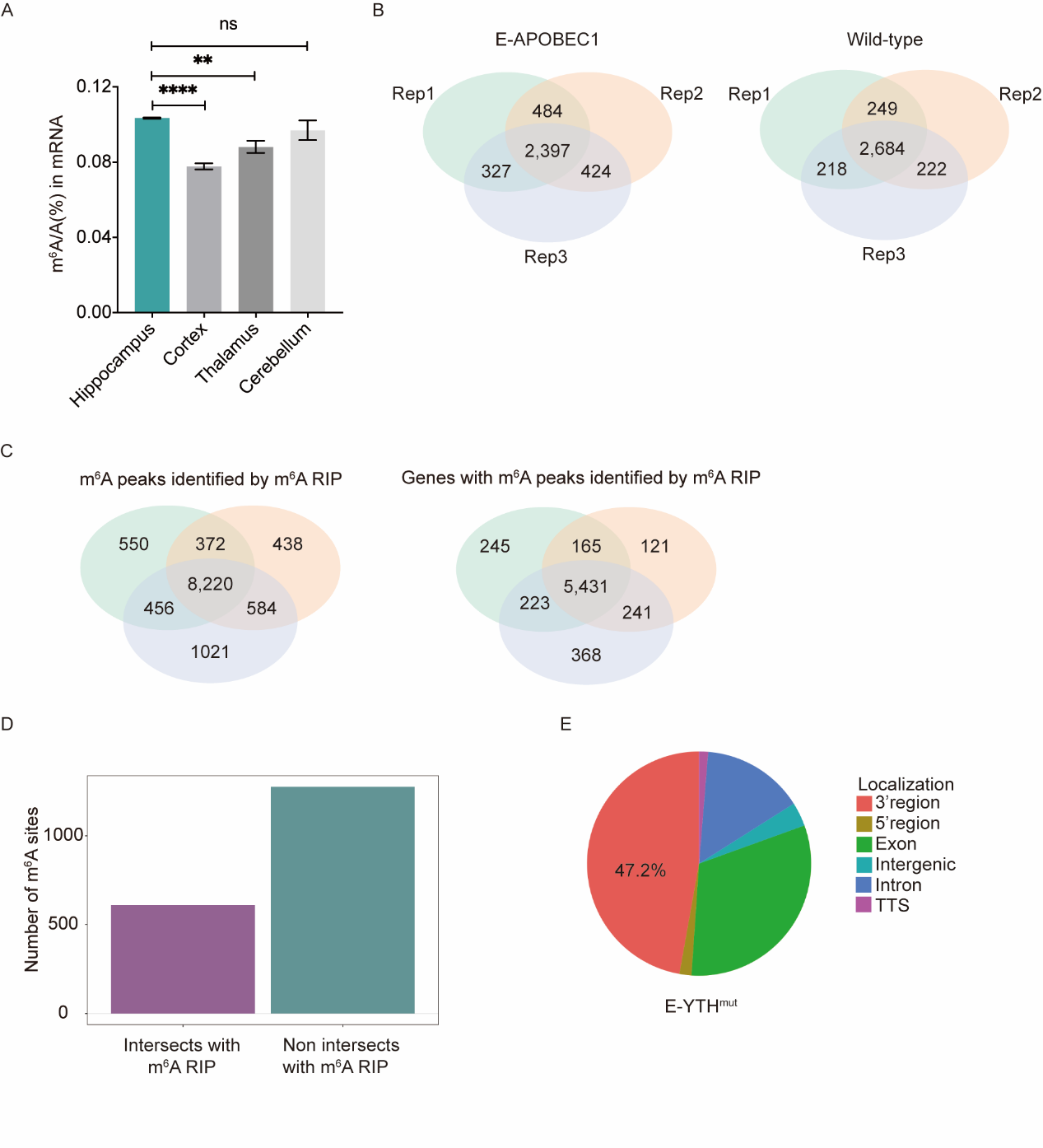


**Supplemental Fig S2. Detection of m^6^A with bulk RNA-seq in mouse hippocampus.**

(A) LC-MS/MS quantification of m^6^A within mRNA relative to unmodified adenosines in mRNA. mRNA was isolated from the hippocampus, cortex, thalamus and cerebellum of 3-month-old mice. n=3. The difference between individual groups compared with the hippocampus was tested using Tukey's multiple comparisons test. *****P*≤0.0001, ****P*≤0.001, ***P*≤0.01. **P*≤0.05.

(B) Number of C-to-U editing events identified in each bulk RNA-seq replicate for controls including E-APOBEC1 and wild type mice. The data was obtained following AAV infection and EGFP FACS sorting. n=3, Rep: biological replicate, hippocampus from one mouse.

(C) Number of m^6^A peaks (left) and number of genes with m^6^A peaks (right) identified in each m^6^A RIP replicate from wild type mouse hippocampi. n=3, Rep: biological replicate, hippocampus from one mouse.

(D) Bar plot showing number of m^6^A sites detected by bulk E-YTH that do and do not intersect with m^6^A peaks identified with m^6^A RIP.

(E) Pie chart showing C-to-U edit localization identified with E-YTH^mut^ in mouse hippocampus. TTS: transcription termination site.
