# Supplementary material for: Single-cell discovery of m6A RNA modifications in the hippocampus
Source: Genome Res. 2024 Jun;34(6):822–36. doi: 10.1101/gr.278424.123 (PMC11293556; doi:10.1101/gr.278424.123)
Supplement: Supplement 3 [file Supplemental_Fig_S3.docx]

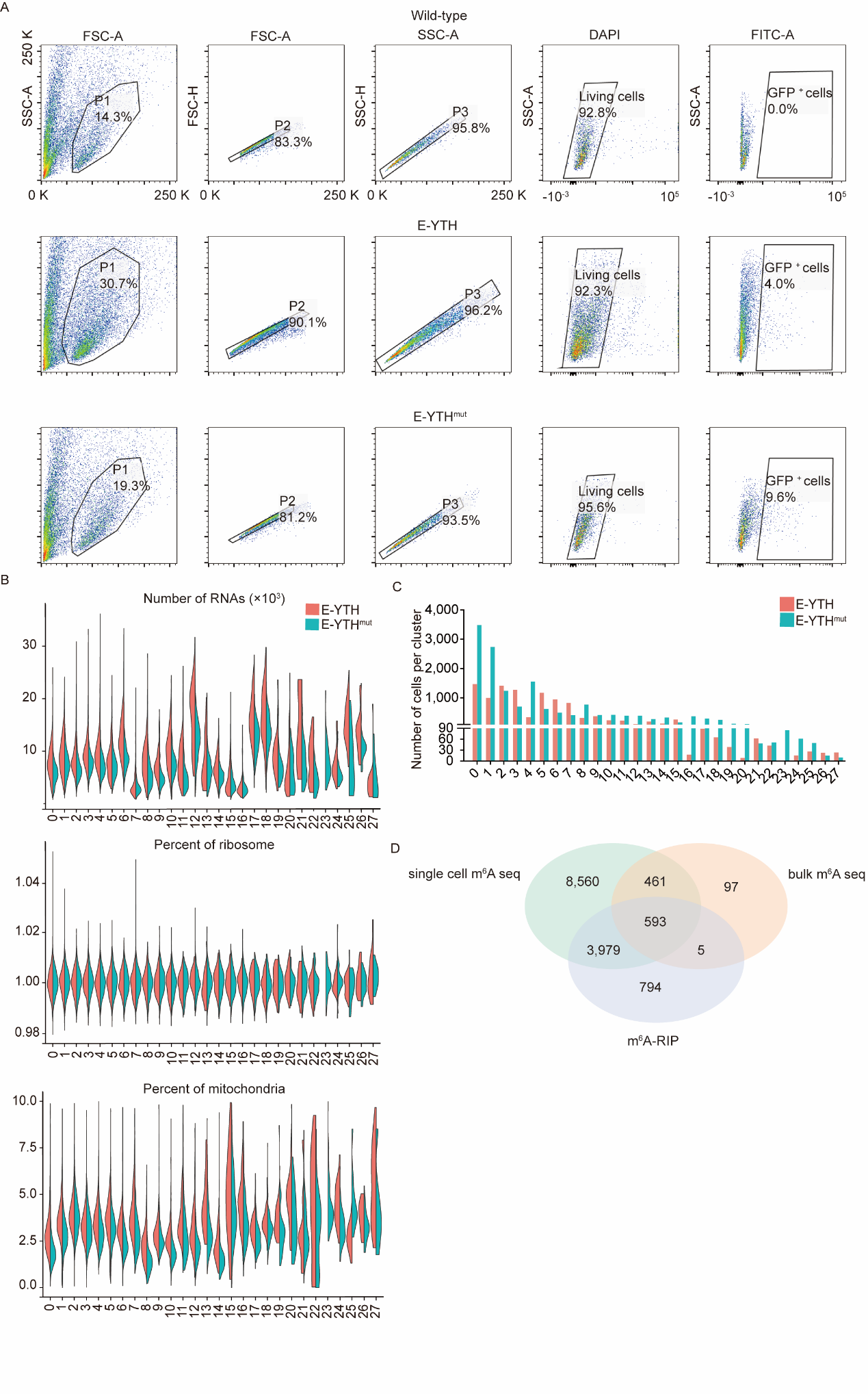


**Supplemental Fig S3. Hippocampal single-cell identification following AAV transduction.**

(A) Isolation of EGFP positive E-YTH, E-YTH^mut^ and wild-type cells from mouse hippocampi. FACS gating is shown. The value indicates percentage of cells in the selected gate.

(B) Single cell quality controls. Top: Violin plot showing the number of RNAs per cell cluster. Middle: Violin plot illustrating the percentage of ribosomal RNA per cell cluster. Bottom: Violin plot visualizing the percentage of mitochondrial RNA per cell cluster.

(C) Bar plot with number of cells identified per cluster.

(D) Venn diagram showing the number of genes with m^6^A regions detected by single cell, bulk and m^6^A RIP RNA-seq.
