# Supplementary material for: Single-cell discovery of m6A RNA modifications in the hippocampus
Source: Genome Res. 2024 Jun;34(6):822–36. doi: 10.1101/gr.278424.123 (PMC11293556; doi:10.1101/gr.278424.123)
Supplement: Supplement 4 [file Supplemental_Fig_S4.docx]

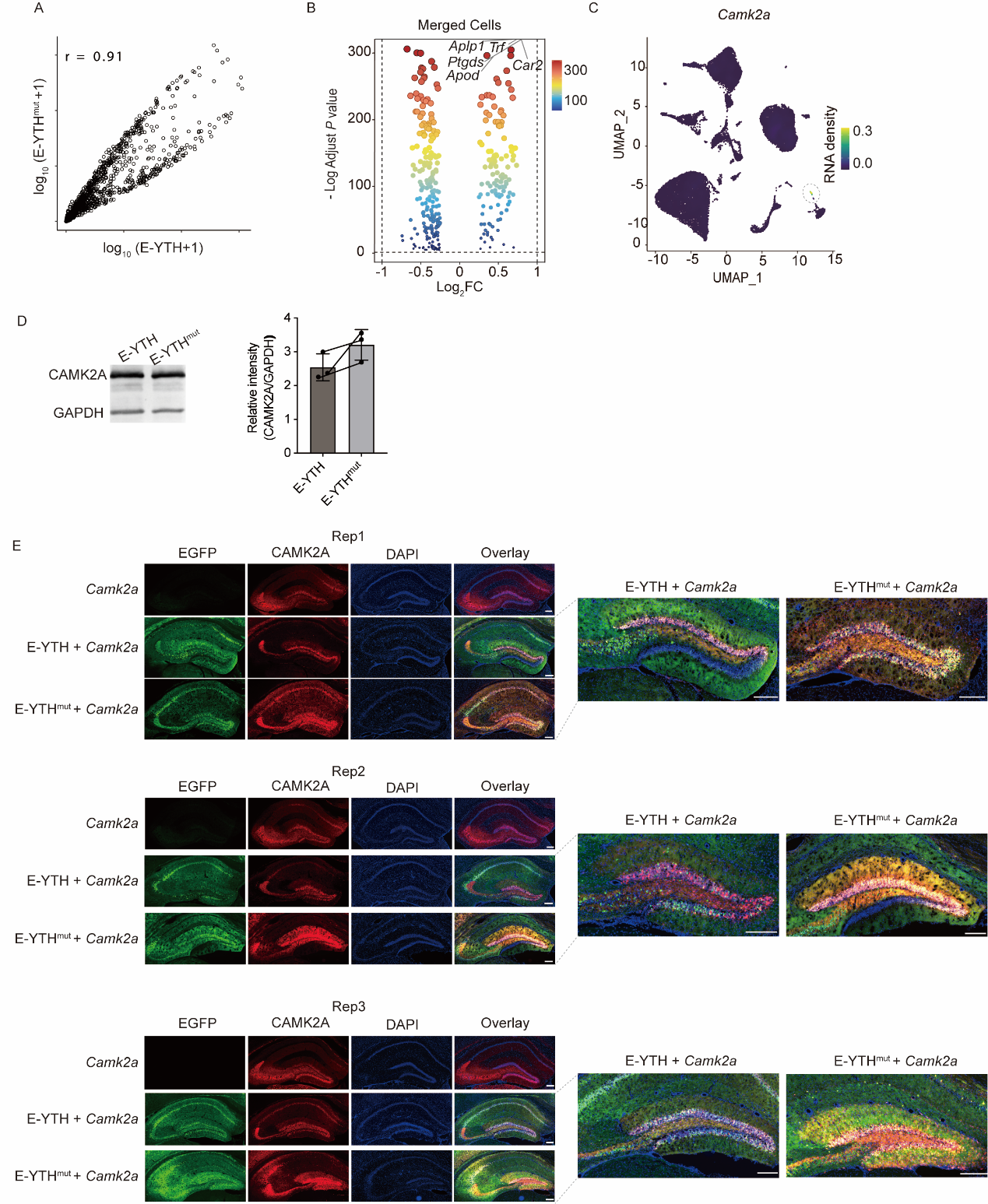


**Supplemental Fig S4. Transcriptional changes detected in E-YTH versus E-YTH^mut^ single-cell data.**

(A) Scatter plot between E-YTH and E-YTH^mut^ single-cell expression data (log_10_TPM+1). Pearson correlation coefficient (*r*) is indicated. TPM: transcripts per million.

(B) Volcano plot comparing RNA levels of E-YTH and E-YTH^mut^ single-cell data. Single-cell data was pooled and plotted to show all merged cells. Adjusted *P* value<0.1 was applied before plotting. Adjusted *P* value<0.05 and a Log_2_FC>1 and Log_2_FC<-1 are considered significant. Colour coded legend value represents -Log_10_ *P* value. FC: Fold change.

(C) Integrated UMAP (E-YTH and E-YTH^mut^) with expression level for *Camk2a*. Legend colour represents RNA density. Circle was added to highlight region with increased RNA density.

(D) Western blot for CAMK2A and GAPDH. Hippocampi were processed following *E-Yth* or *E-Yth^mut^* AAV injection. Normalized quantifications are shown. n=3 biological replicates.

(E) *Camk2a-mCherry* AAV was mixed with equal amounts of *Egfp* expressing E-YTH and E-YTH^mut^ AAVs along with the *Camk2a-mCherry* AAV alone and the mix was subsequently injected into mouse hippocampi. Hippocampi were imaged to evaluate colocalization of CAMK2A and EGFP signals. CAMK2A expressing cells appear red, E-YTH and E-YTH^mut^ expressing cells are green. Bottom images show increased zoom. In comparison to other AAV injection experiments, due to mixing with *Camk2a-mCherry* AAV, the amount of *E-Yth* and *E-Yth^mut^* AAV viruses injected here is reduced by half. Scale bar: 200µm; Rep1-3: biological replicates 1-3.
