# Supplementary material for: Single-cell discovery of m6A RNA modifications in the hippocampus
Source: Genome Res. 2024 Jun;34(6):822–36. doi: 10.1101/gr.278424.123 (PMC11293556; doi:10.1101/gr.278424.123)
Supplement: Supplement 5 [file Supplemental_Fig_S5.docx]

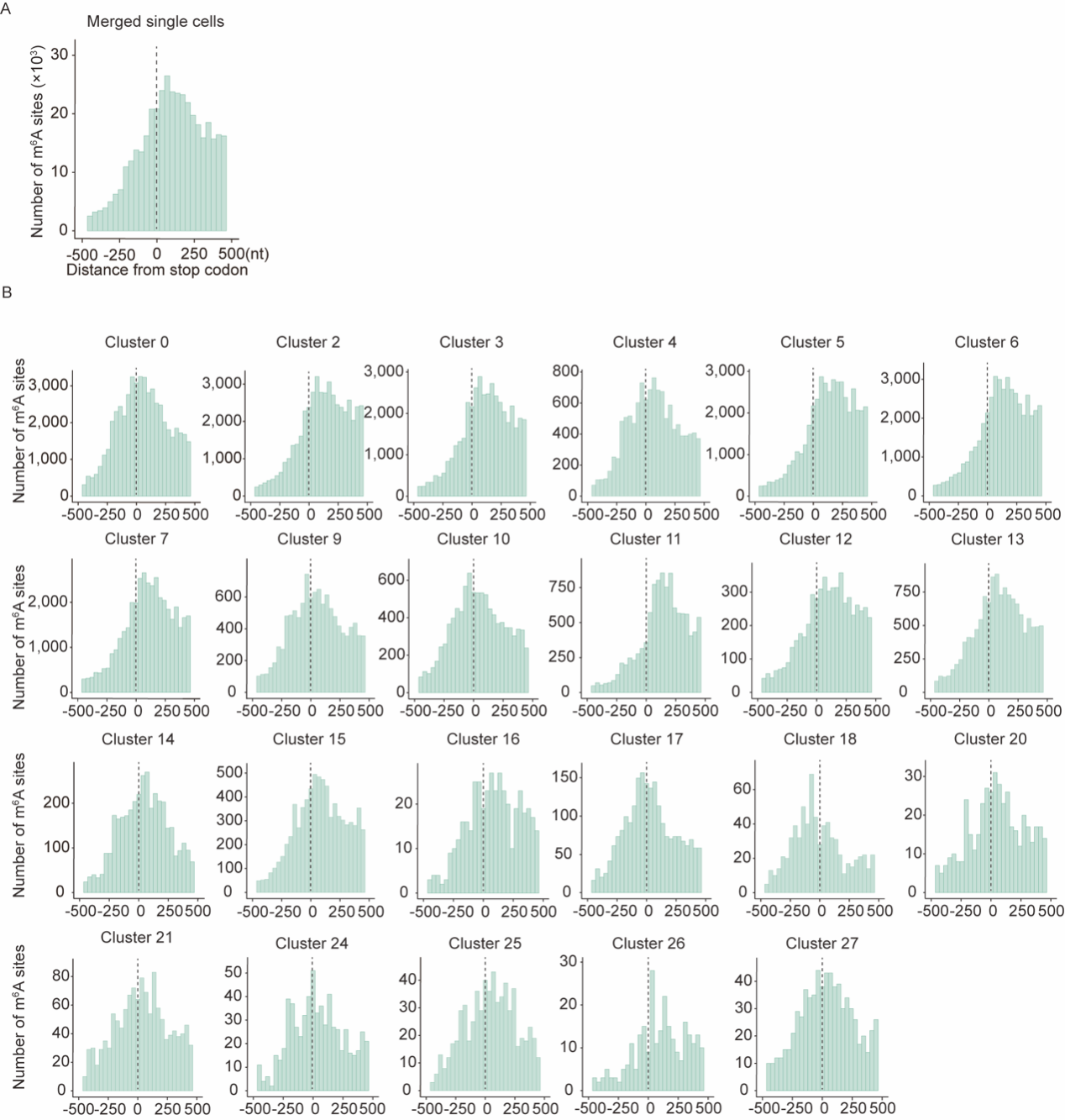


**Supplemental Fig S5. Hippocampal single-cell metagene analysis surrounding the stop codon.**

(A) Metagene analysis of all cells merged identified by single-cell sequencing. m^6^A number surrounding the stop codon (position 0). m^6^A sites were obtained after eliminating background editing sites.

(B) Metagene analysis of individual cell clusters identified by single-cell sequencing. Clusters 0 through 7, 9 through 21 and 24 through 27 are shown. m^6^A number surrounding the stop codon (position 0). m^6^A sites were obtained after eliminating background editing sites.
