# Supplementary material for: Single-cell discovery of m6A RNA modifications in the hippocampus
Source: Genome Res. 2024 Jun;34(6):822–36. doi: 10.1101/gr.278424.123 (PMC11293556; doi:10.1101/gr.278424.123)
Supplement: Supplement 6 [file Supplemental_Fig_S6.docx]

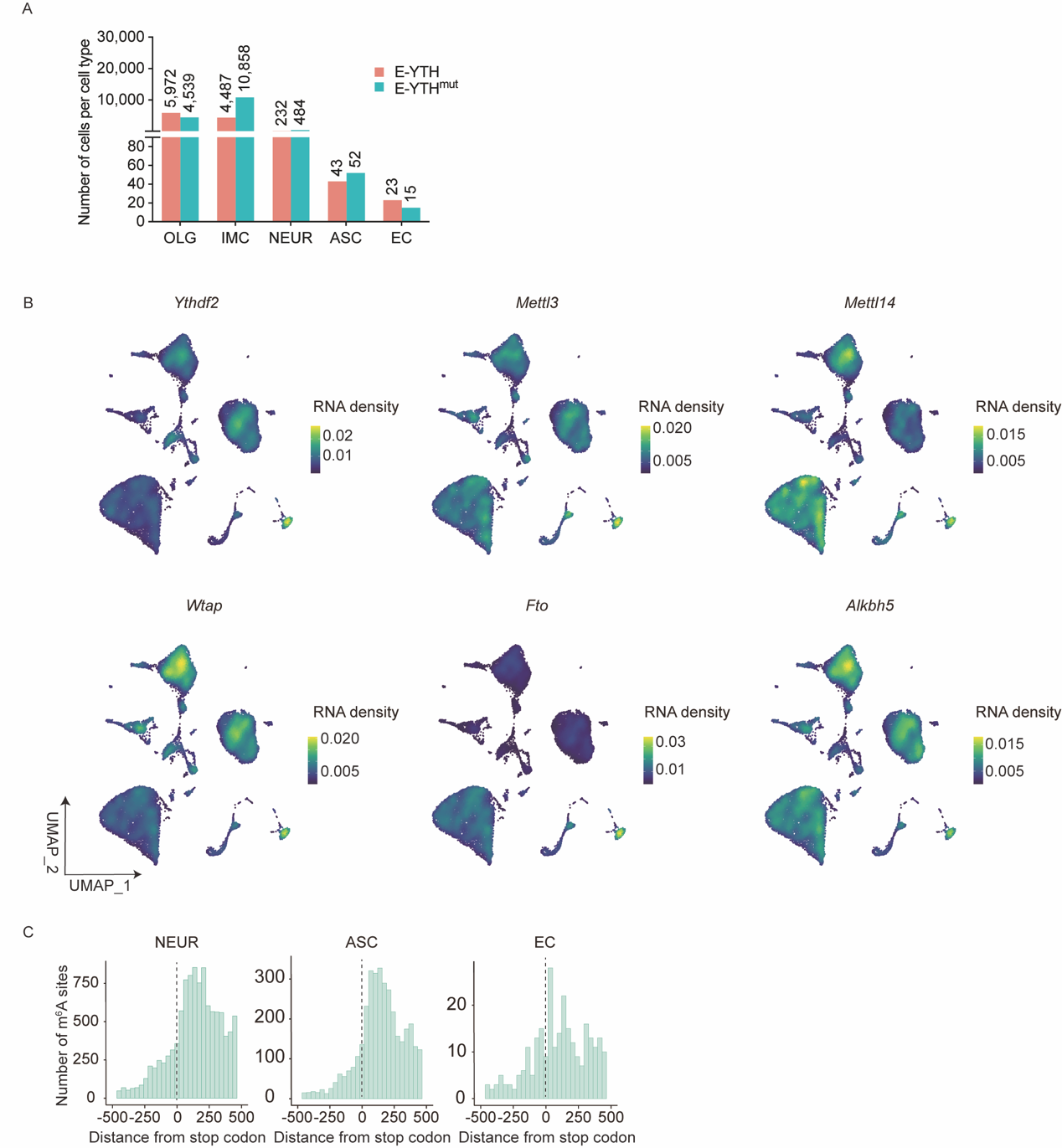


**Supplemental Fig S6. Hippocampal m^6^A characteristics.**

(A) Bar chart showing number of cells per cell type identified. OLG: oligodendrocyte cell lineage; IMC: immune cell lineage; NEUR: neuronal cell lineage; ASC: astrocyte cell lineage; EC: endothelial cell lineage.

(B) UMAP plot of single cell gene expression of *Ythdf2*, *Mettl3*, *Mettl14* and *Wtap* encoding m^6^A methylases and of *Fto* and *Alkbh5* encoding m^6^A demethylases. Legend colour represents RNA density.

(C) Distribution of m^6^A surrounding the stop codon (0nt) identified by single cell sequencing for 3 main cell types. NEUR: neuronal cell lineage; ASC: astrocyte cell lineage; EC: endothelial cell lineage;
