# Supplementary material for: Single-cell discovery of m6A RNA modifications in the hippocampus
Source: Genome Res. 2024 Jun;34(6):822–36. doi: 10.1101/gr.278424.123 (PMC11293556; doi:10.1101/gr.278424.123)
Supplement: Supplement 7 [file Supplemental_Fig_S7.docx]

m6A sites E-YTHmut


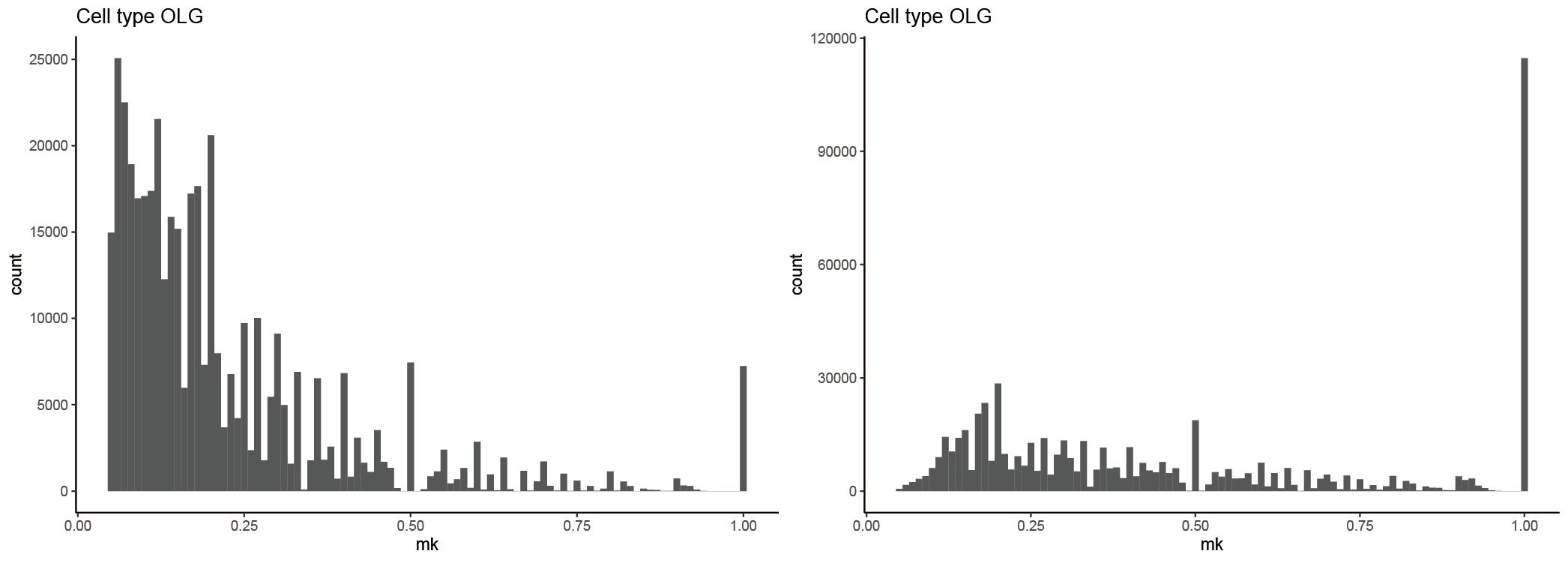


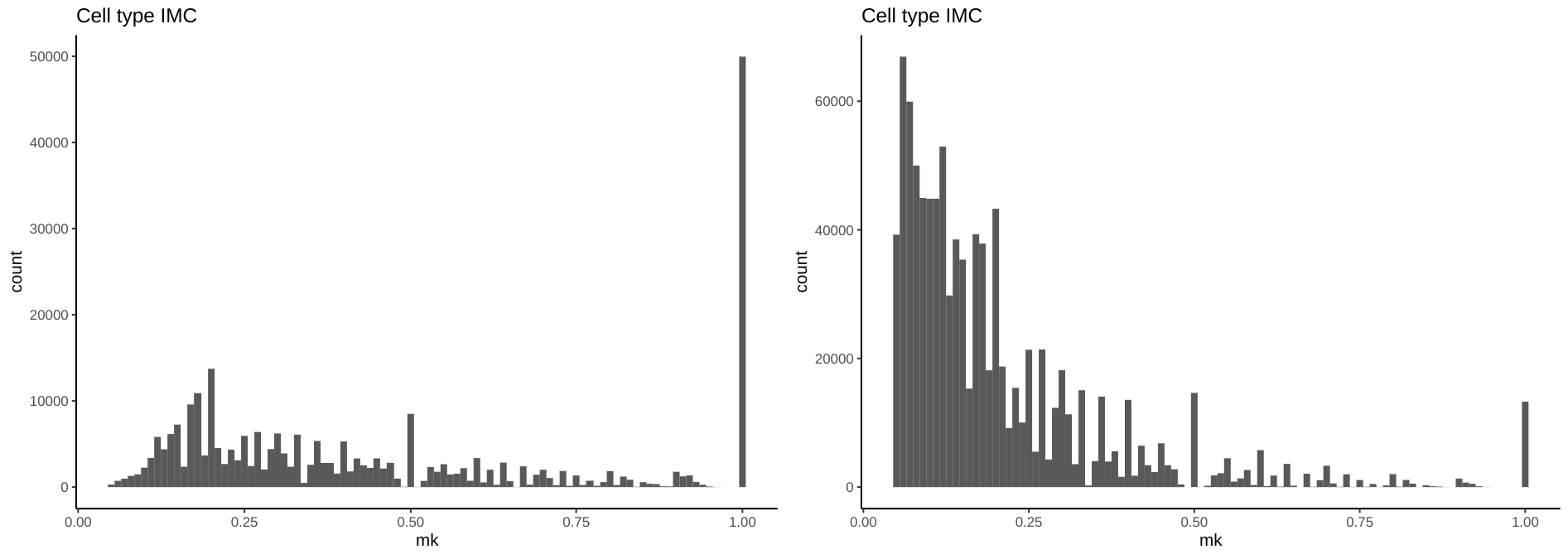
m6A sites E-YTHmut


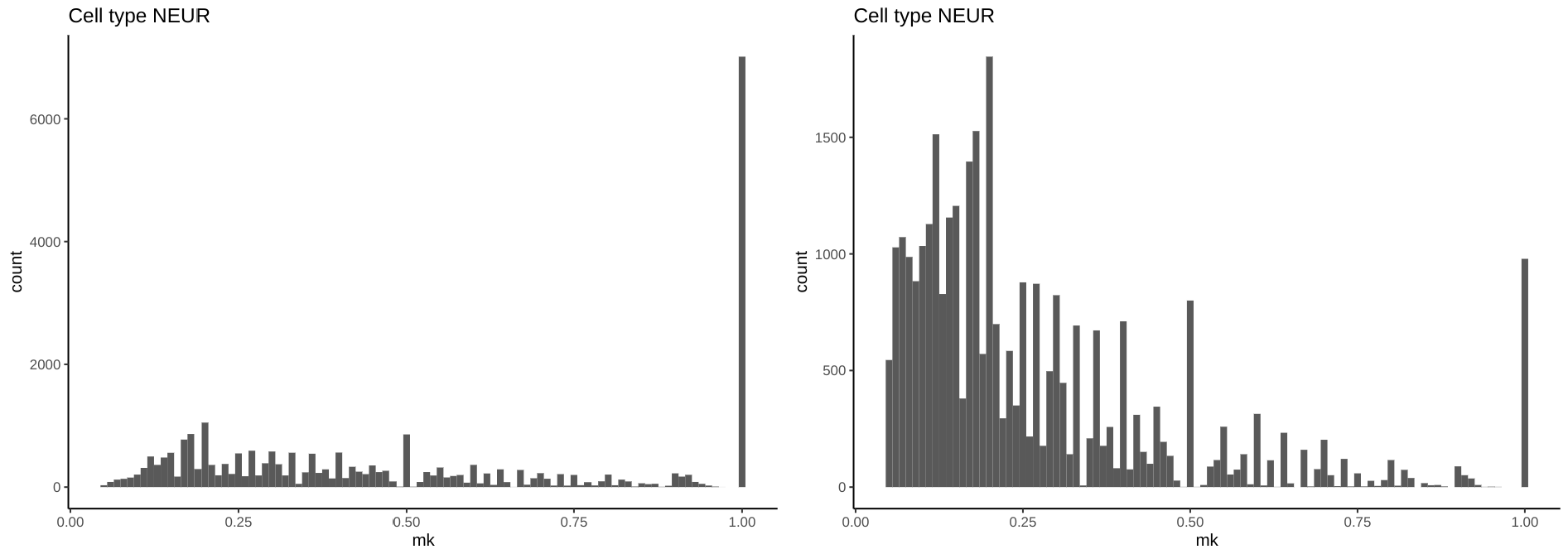
m6A sites E-YTHmut


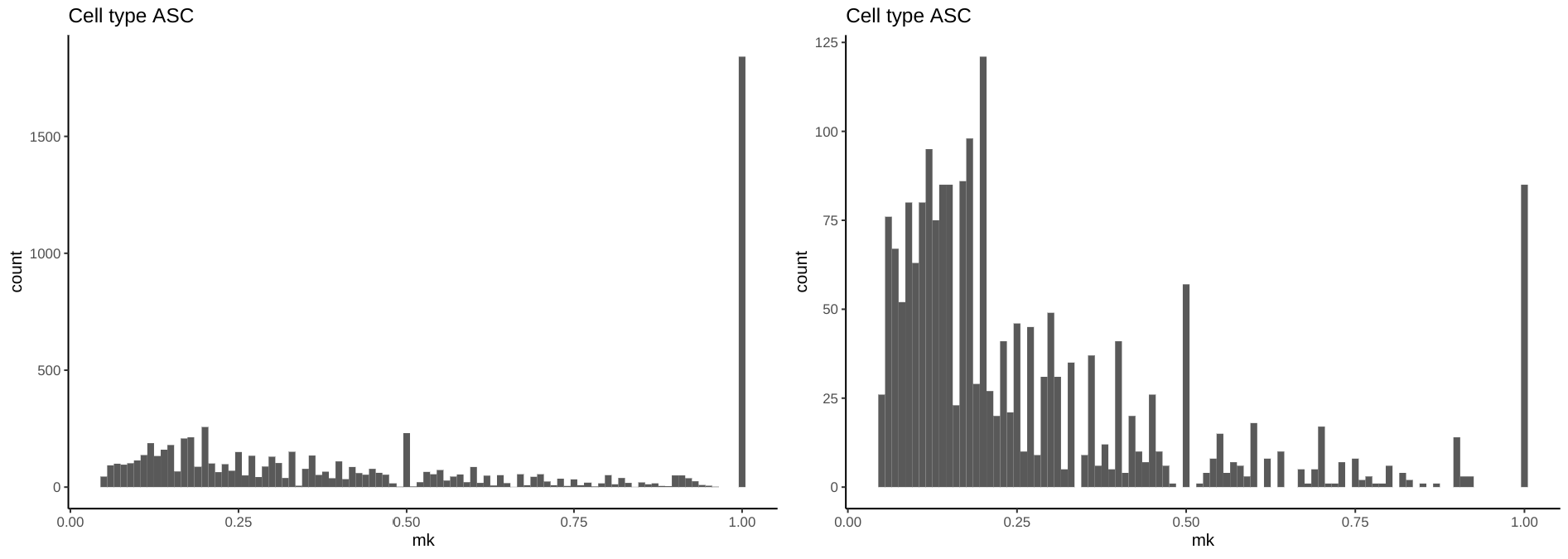
m6A sites E-YTHmut


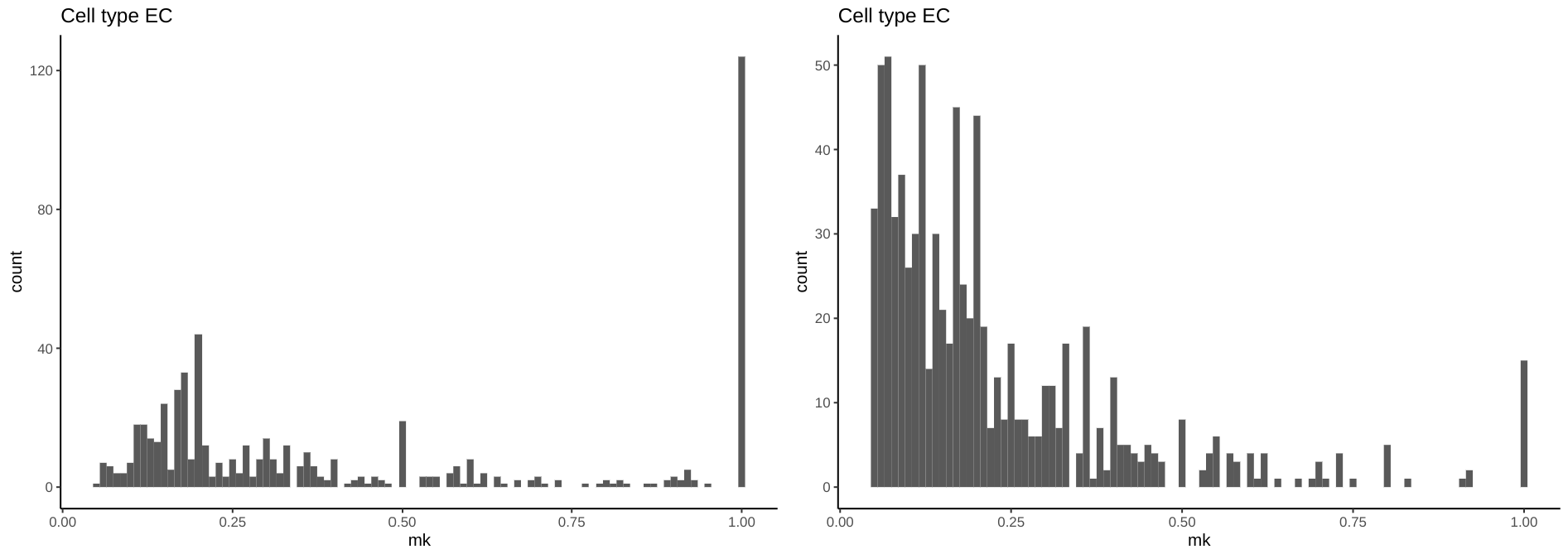
m6A sites E-YTHmut


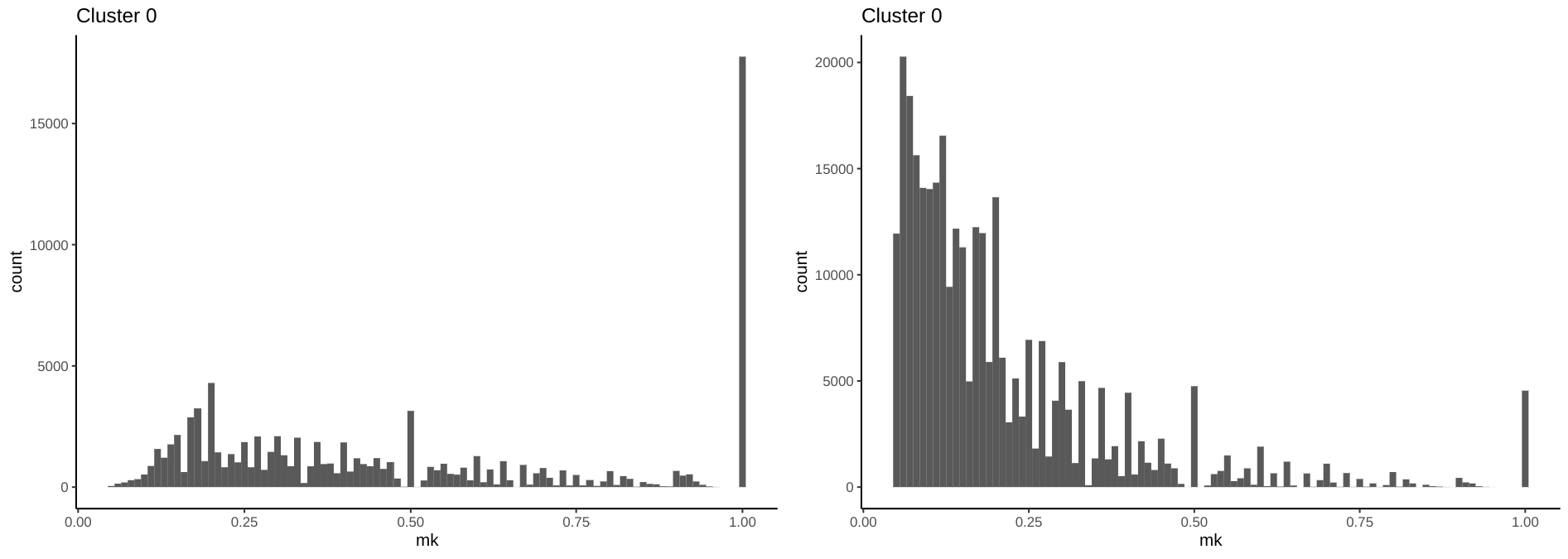
m6A sites E-YTHmut


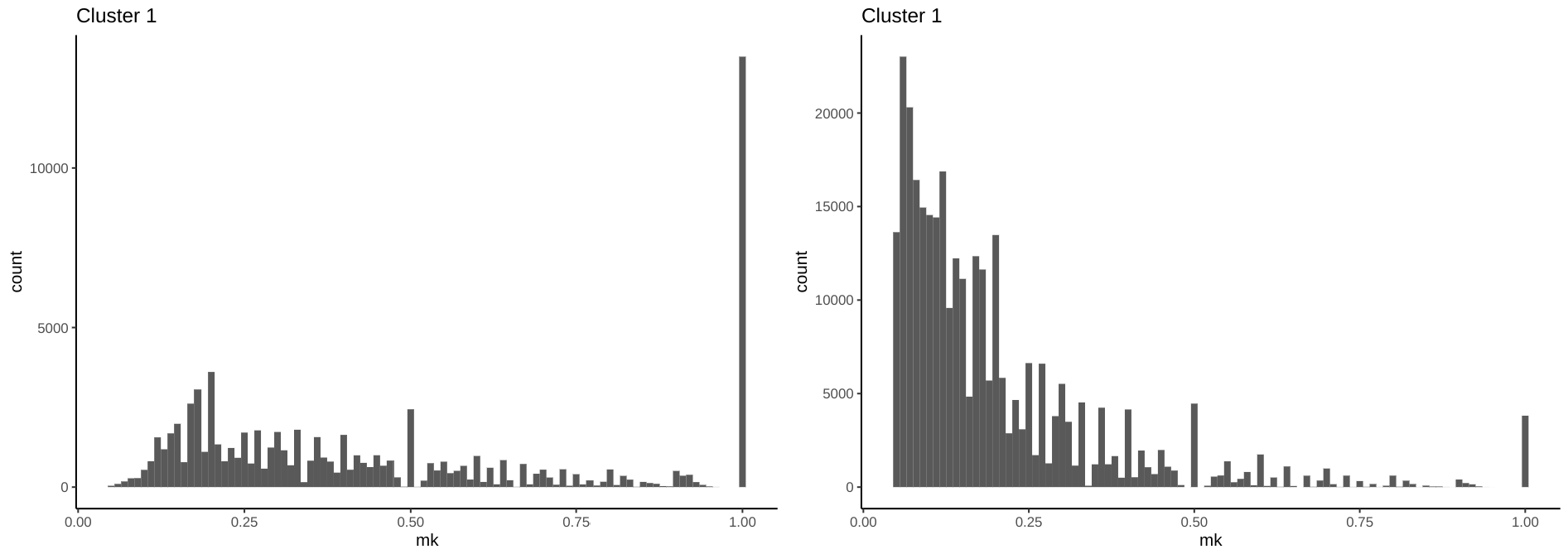
m6A sites E-YTHmut


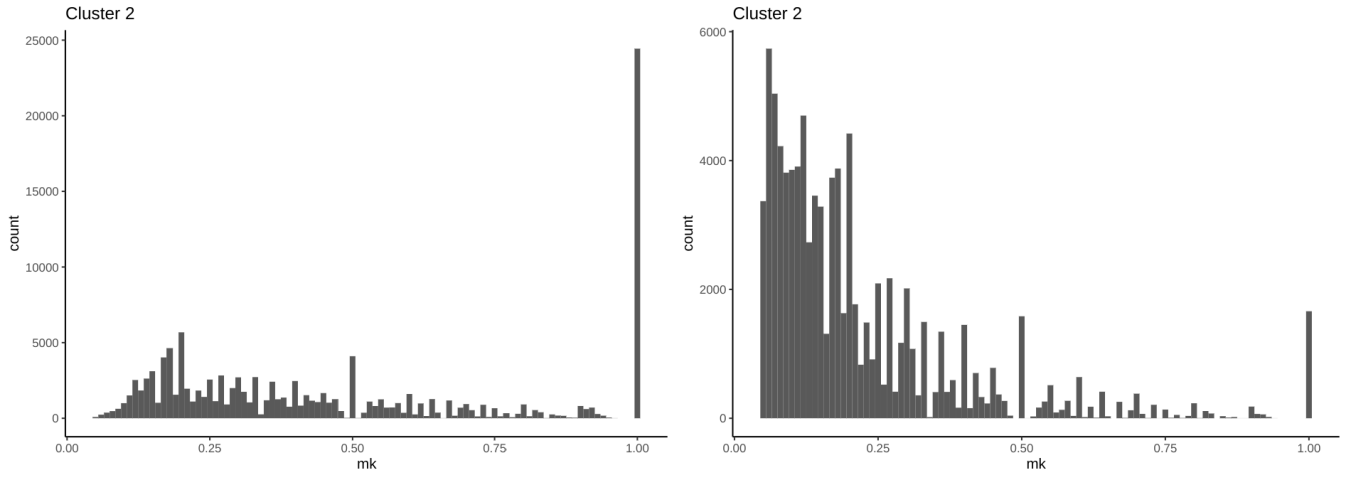
m6A sites E-YTHmut


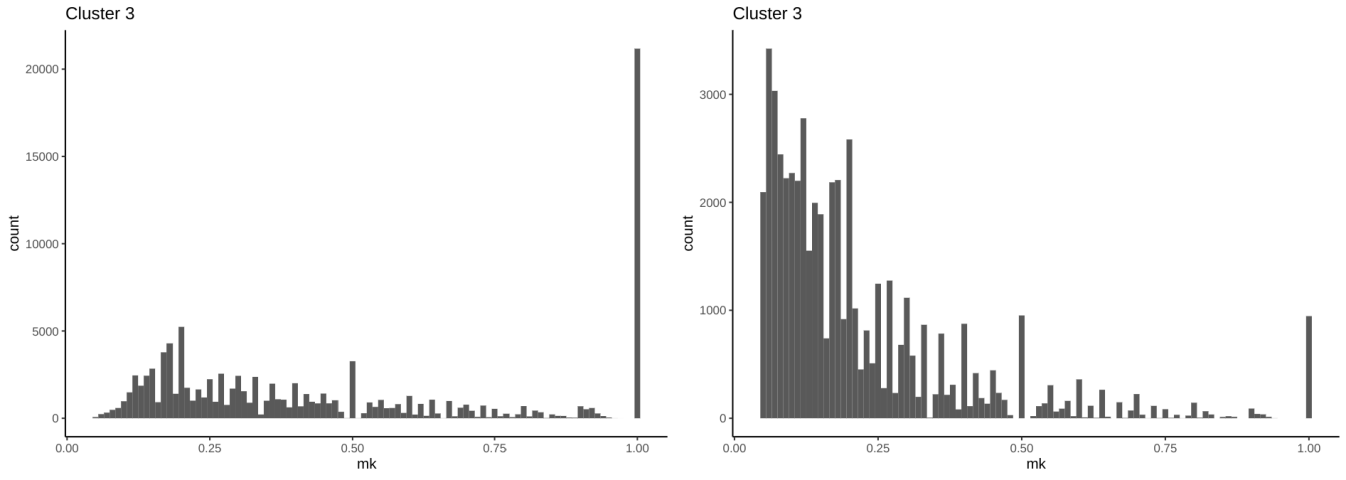
m6A sites E-YTHmut


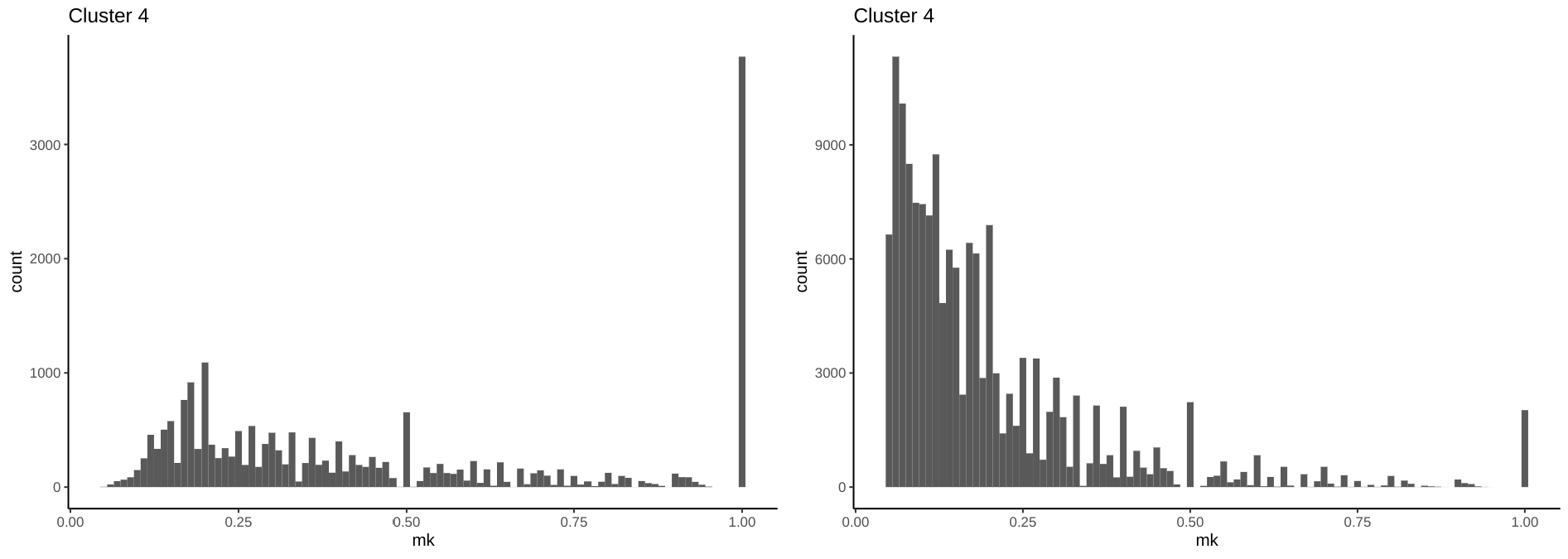
m6A sites E-YTHmut


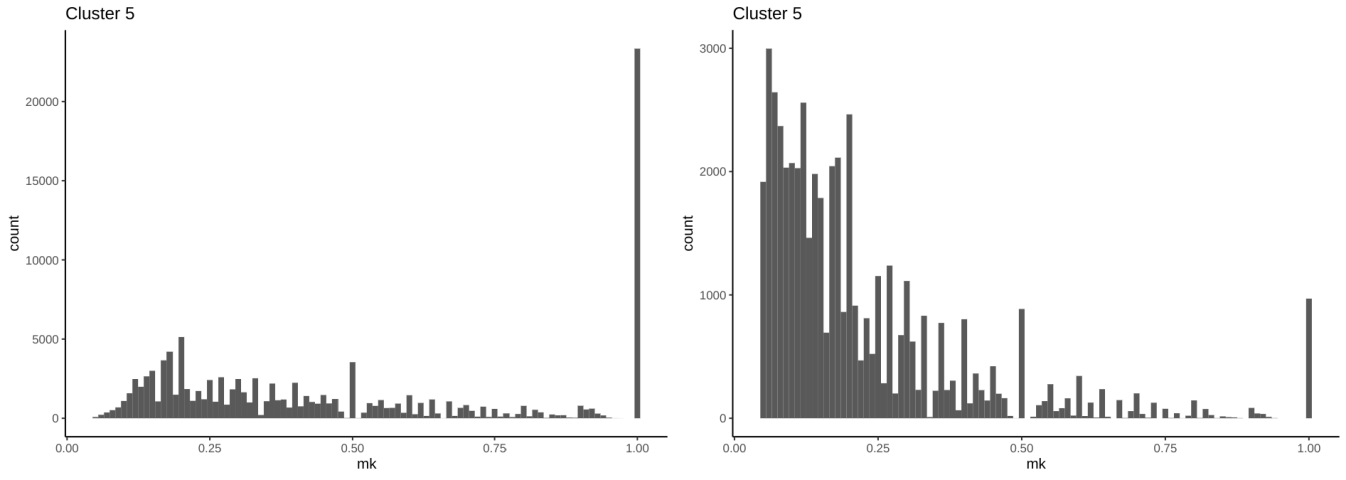
m6A sites E-YTHmut


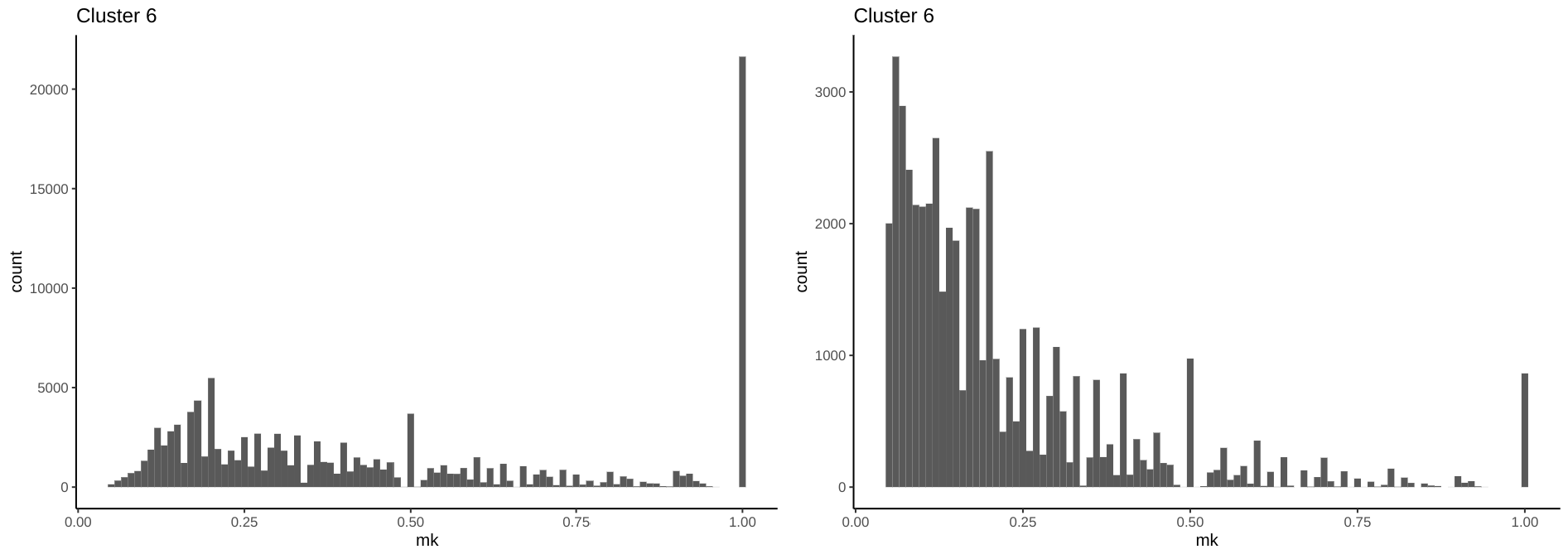
m6A sites E-YTHmut


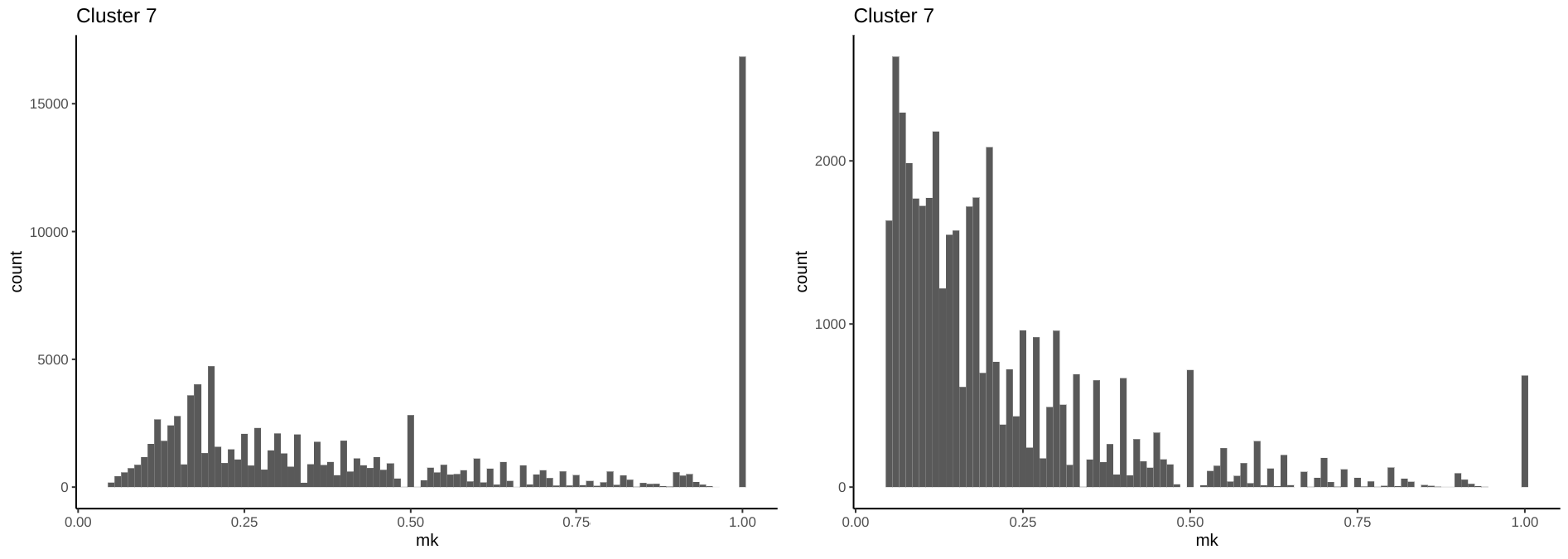
m6A sites E-YTHmut


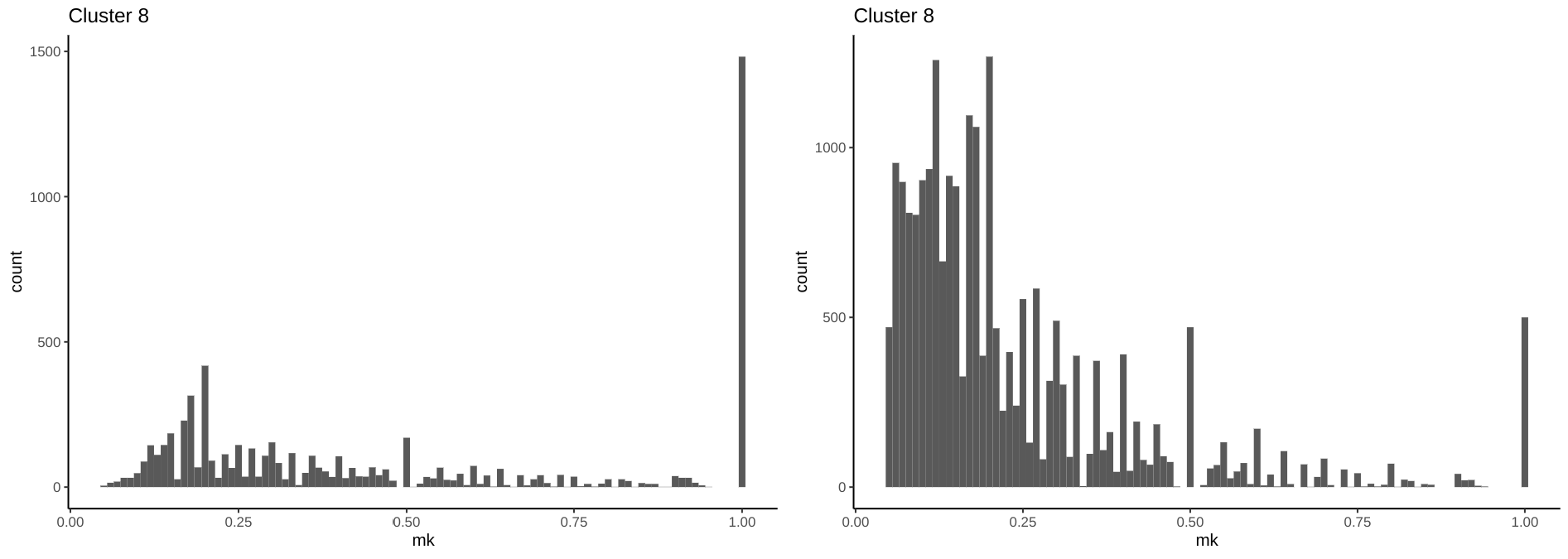
m6A sites E-YTHmut


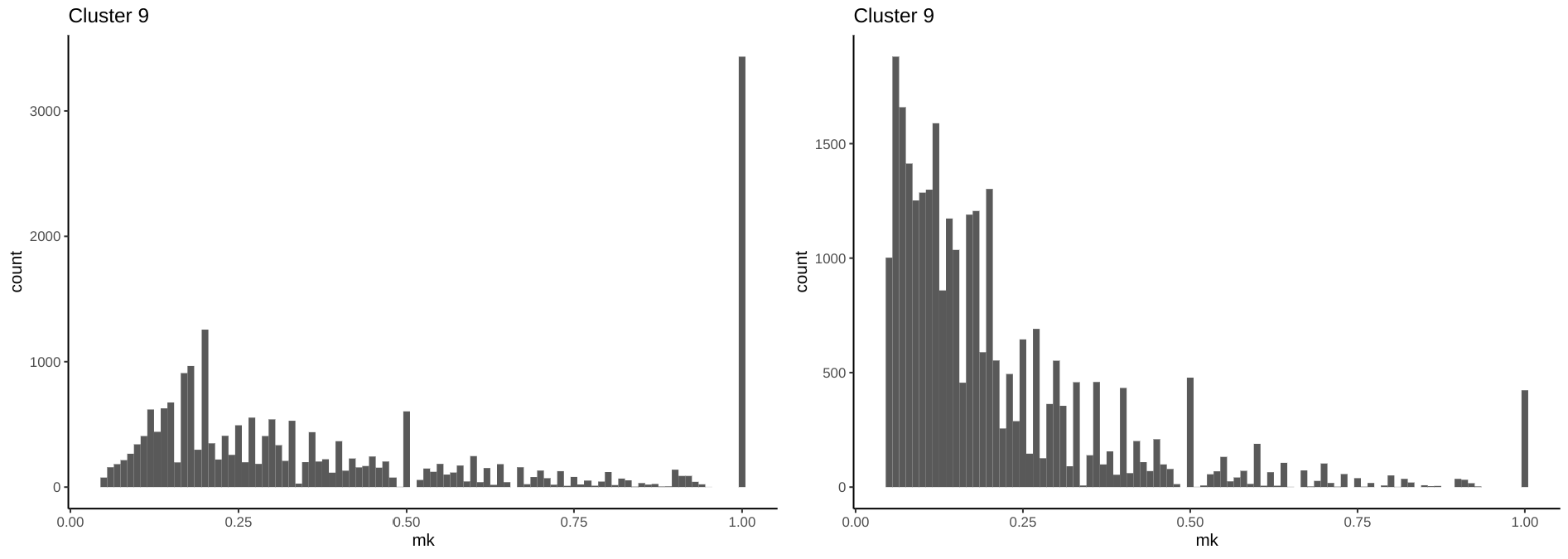
m6A sites E-YTHmut


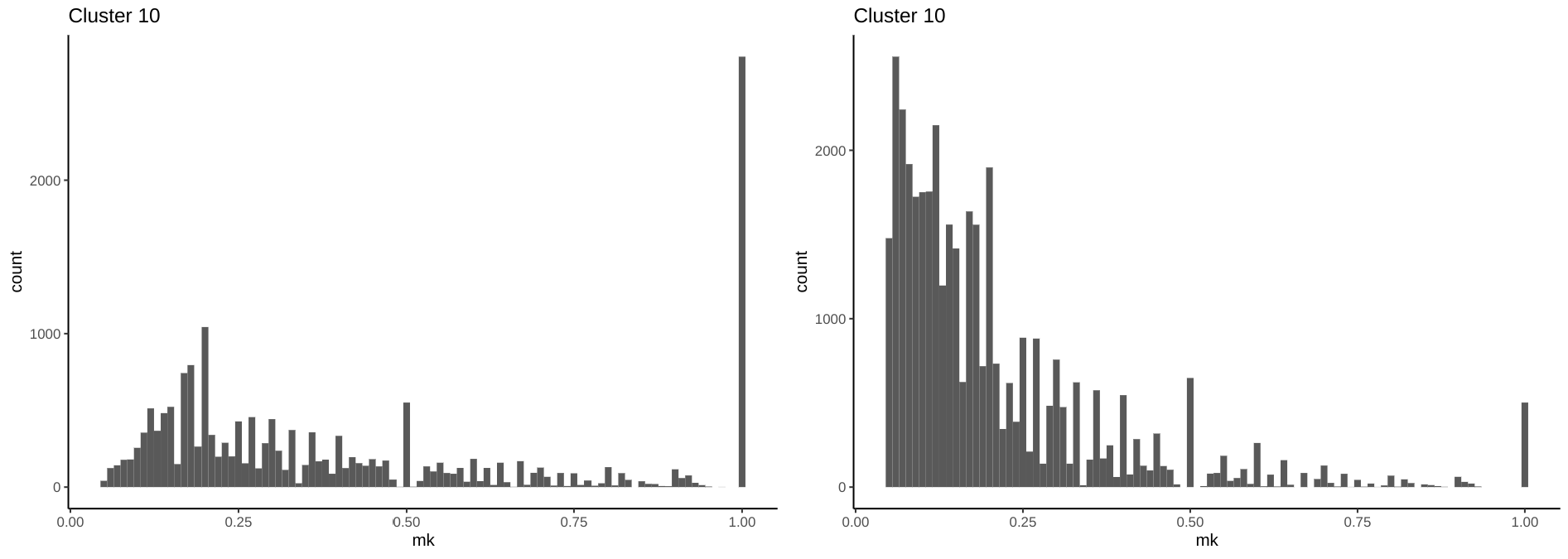
m6A sites E-YTHmut


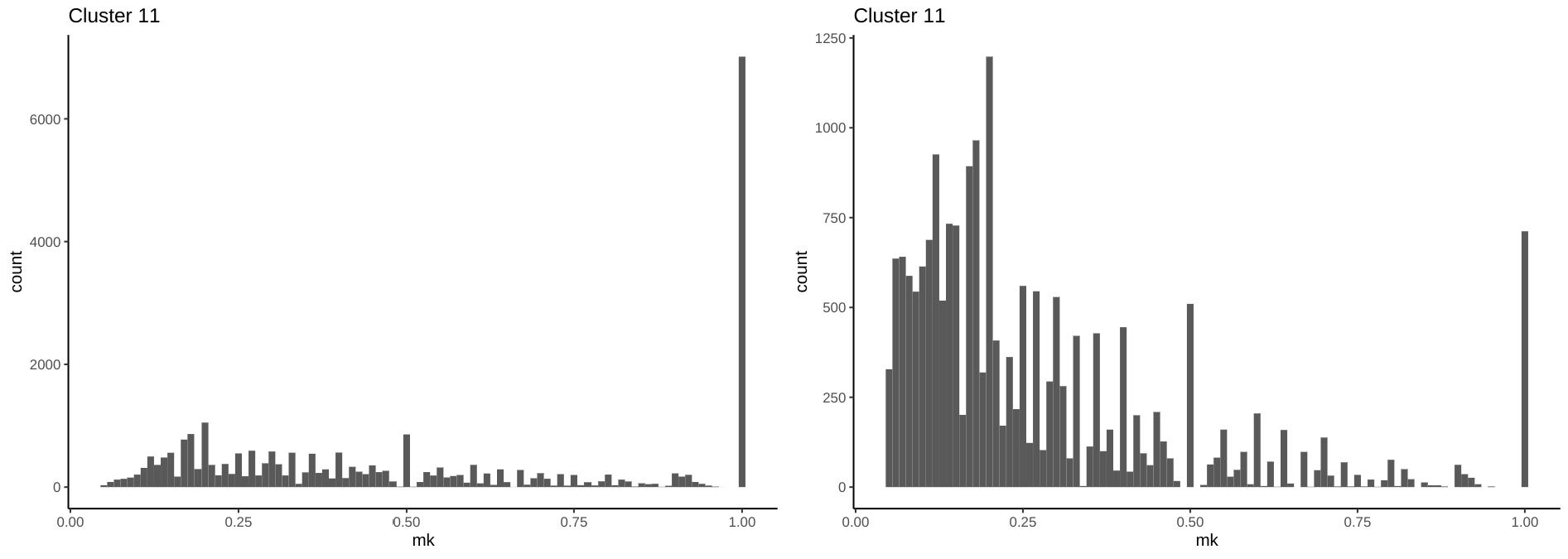
m6A sites E-YTHmut


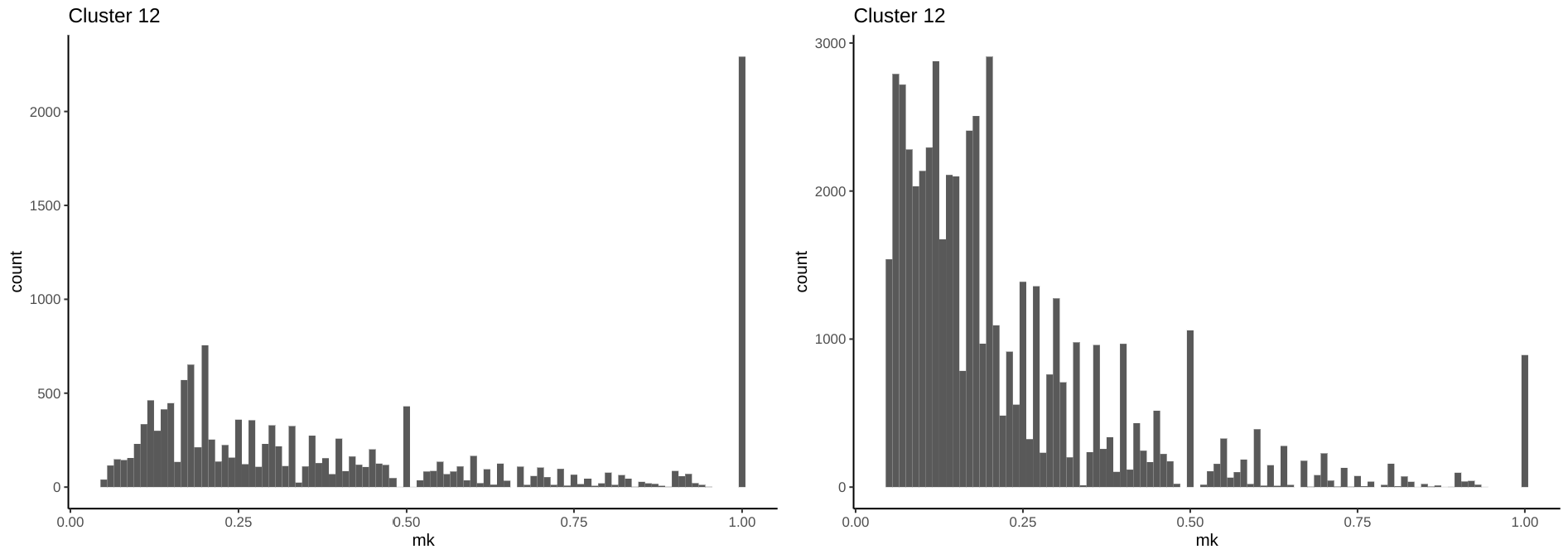
m6A sites E-YTHmut


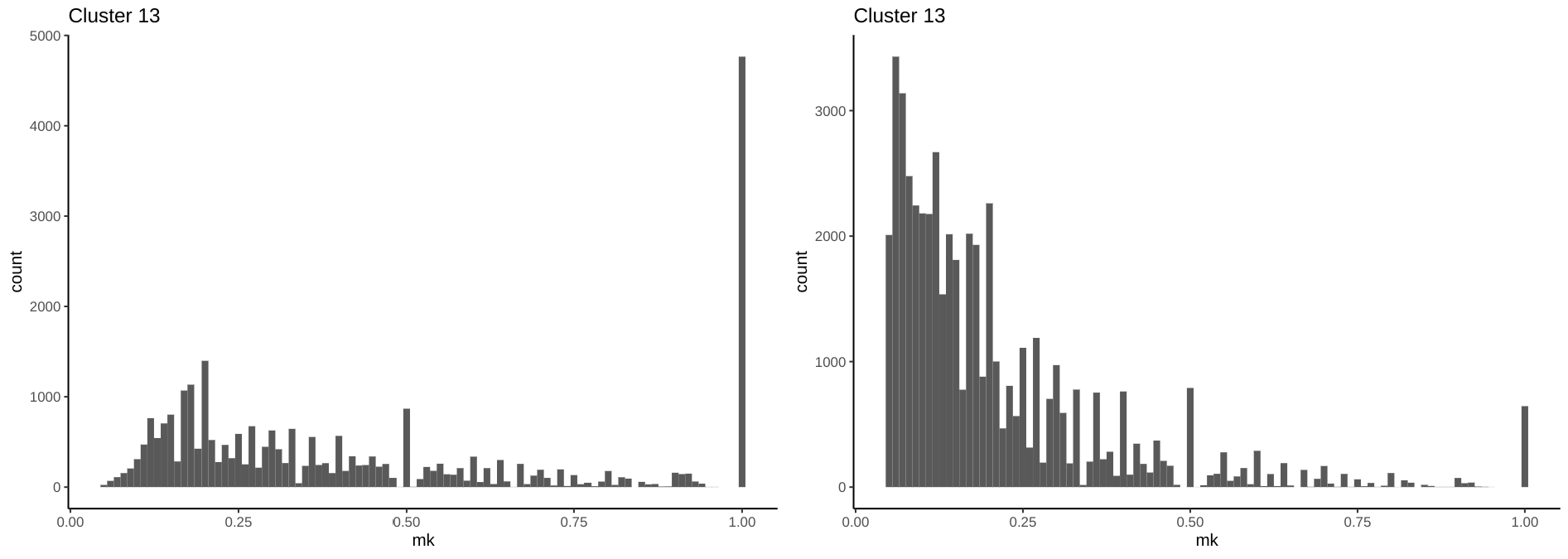
m6A sites E-YTHmut


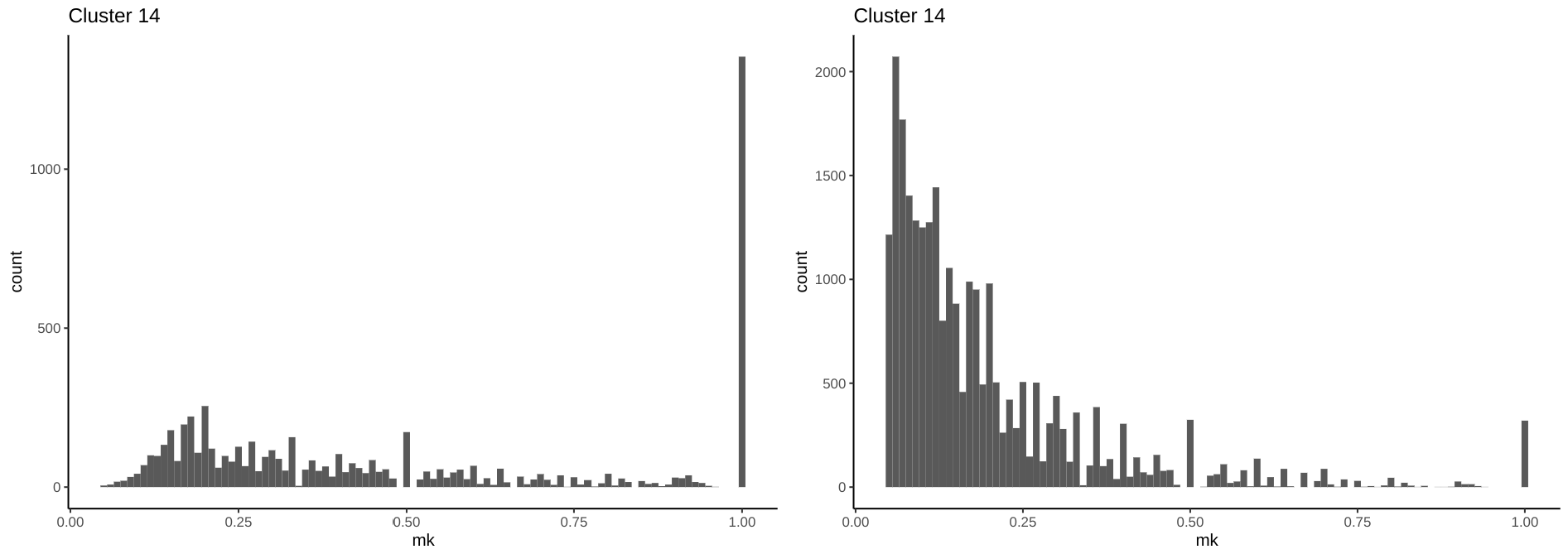
m6A sites E-YTHmut


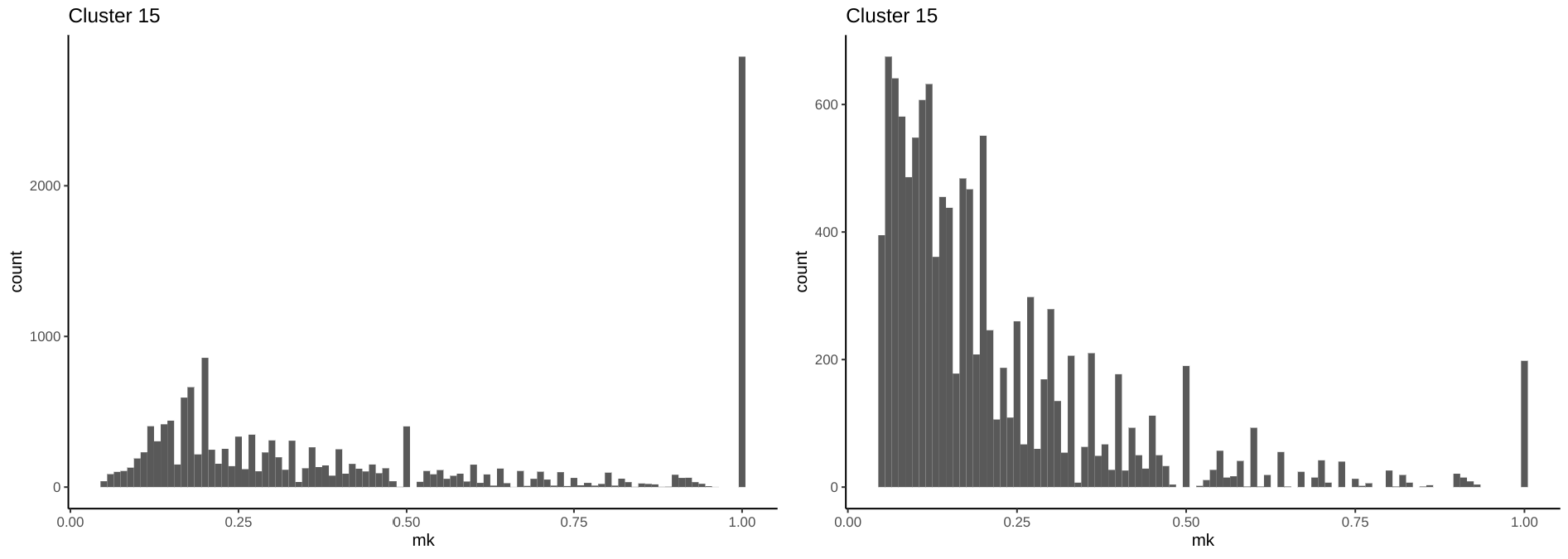
m6A sites E-YTHmut


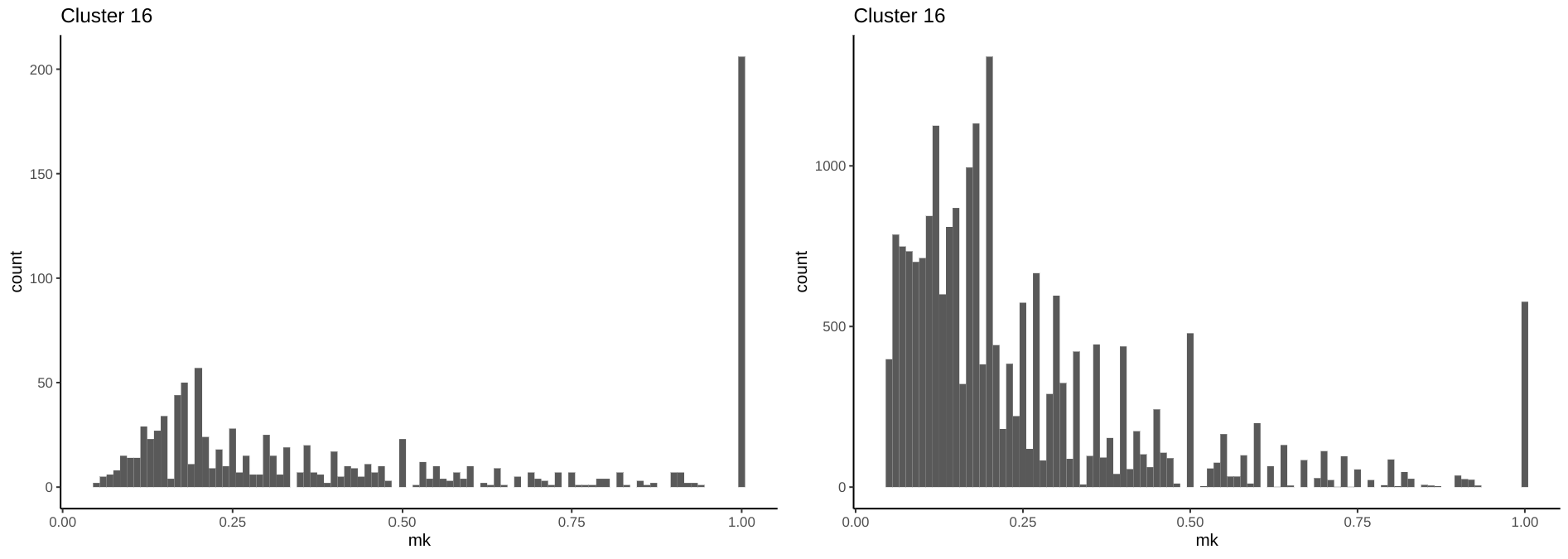
m6A sites E-YTHmut


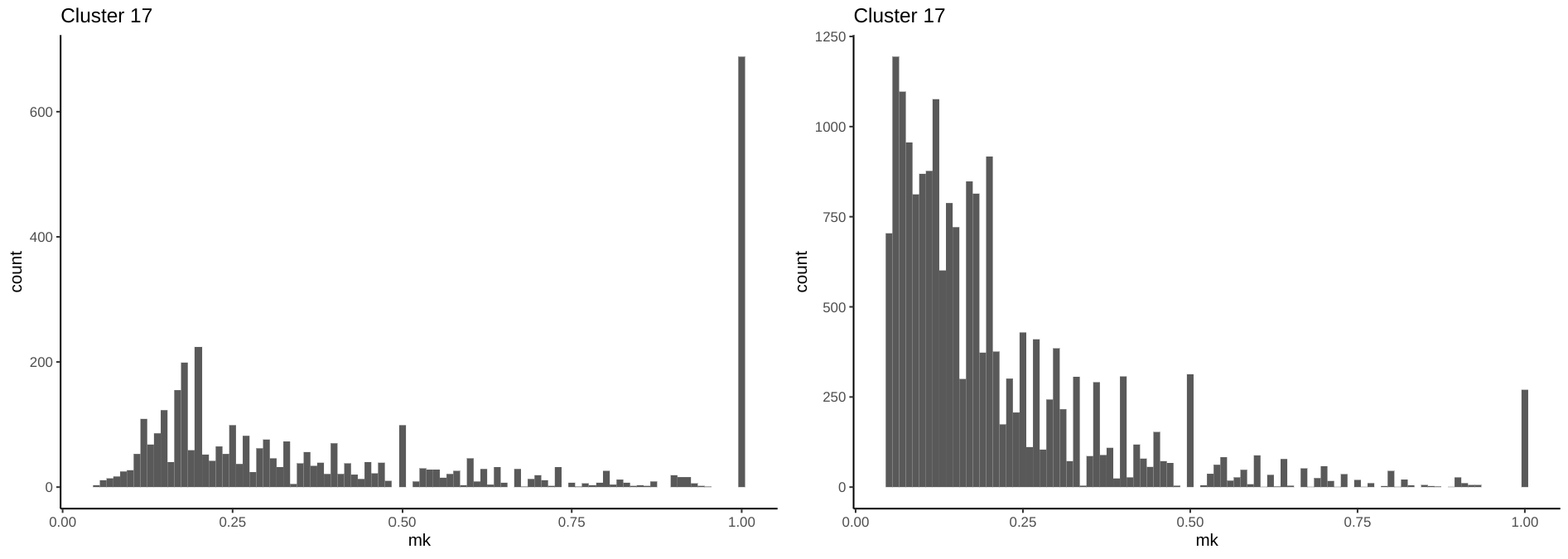
m6A sites E-YTHmut


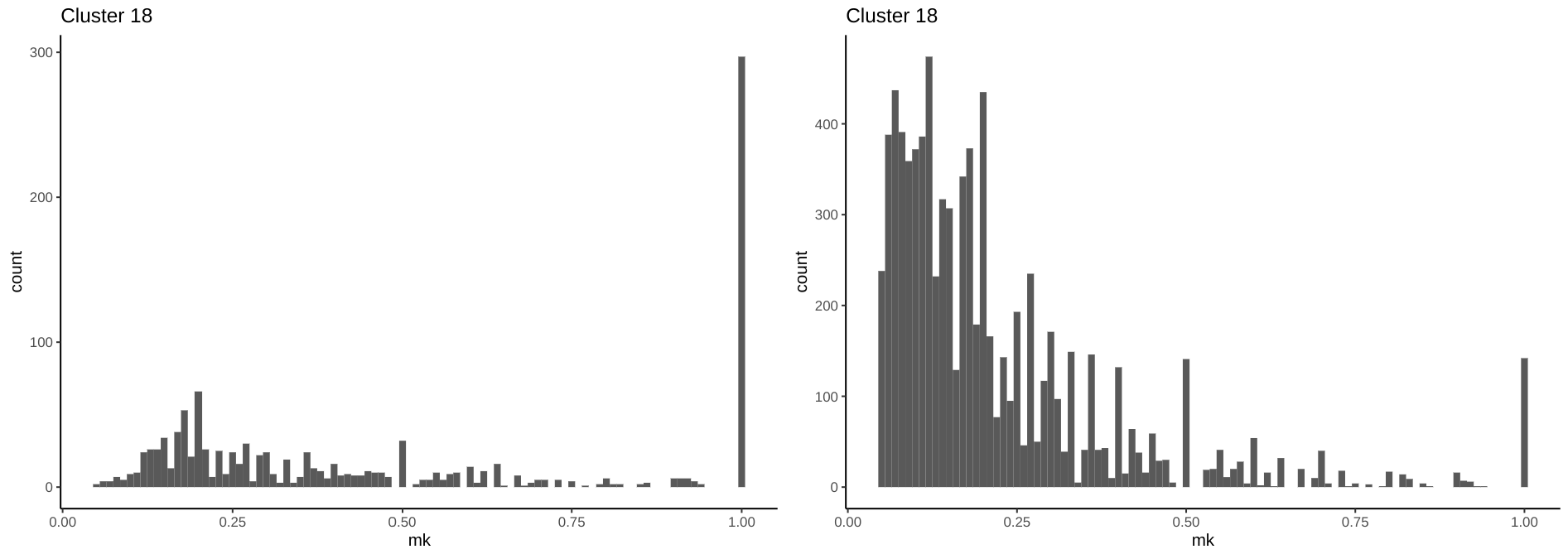
m6A sites E-YTHmut


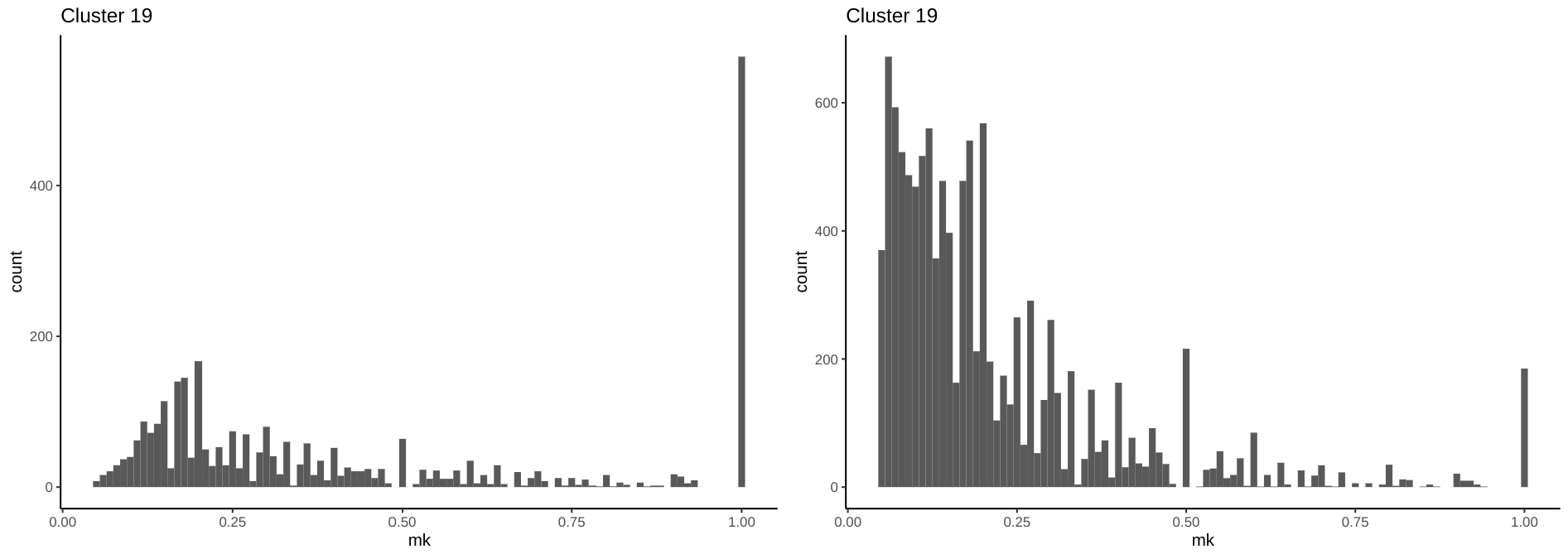
m6A sites E-YTHmut


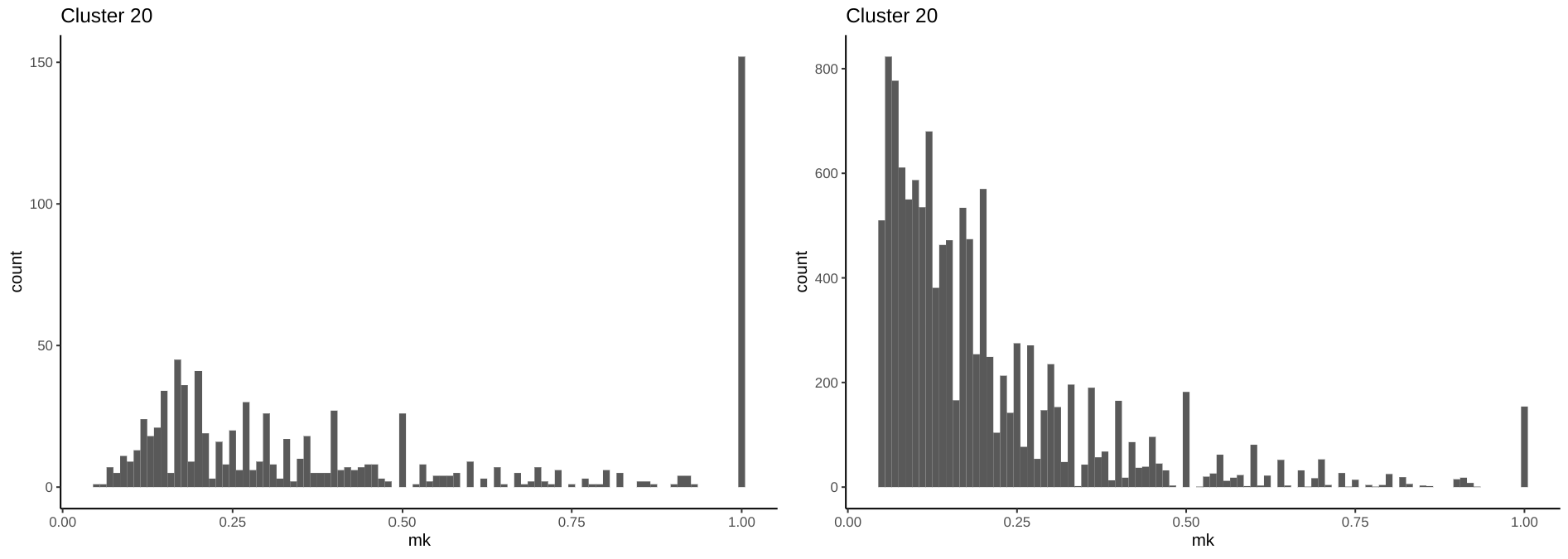
m6A sites E-YTHmut


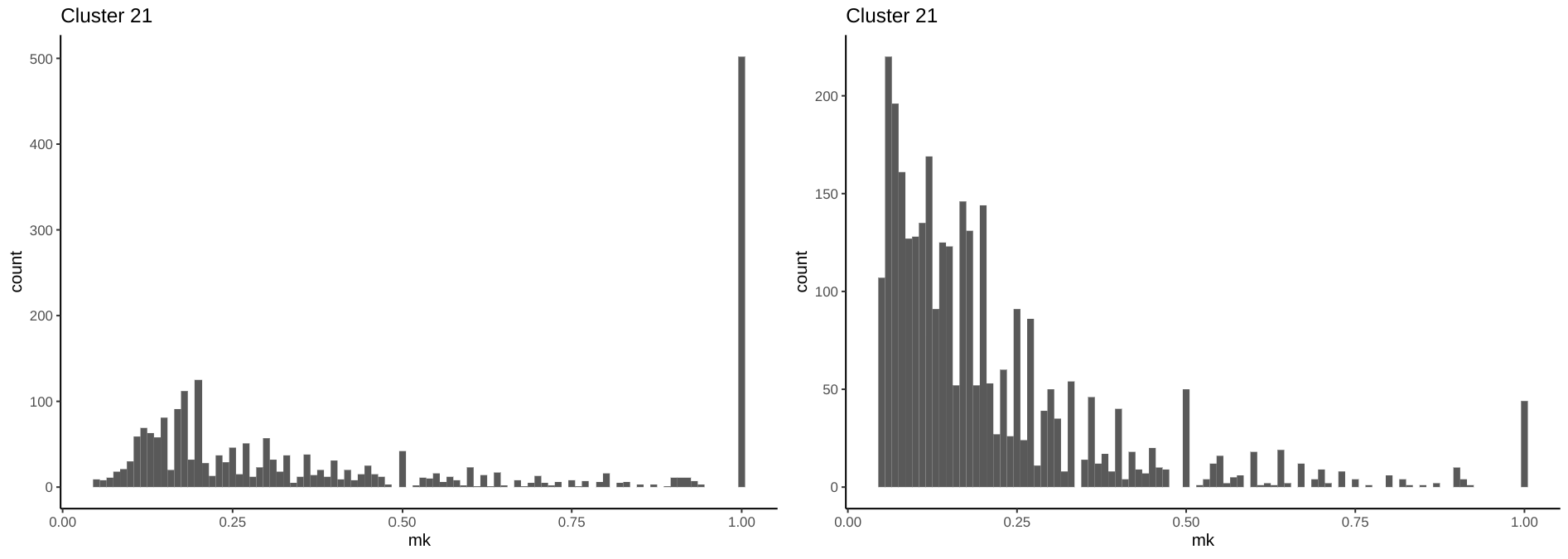
m6A sites E-YTHmut


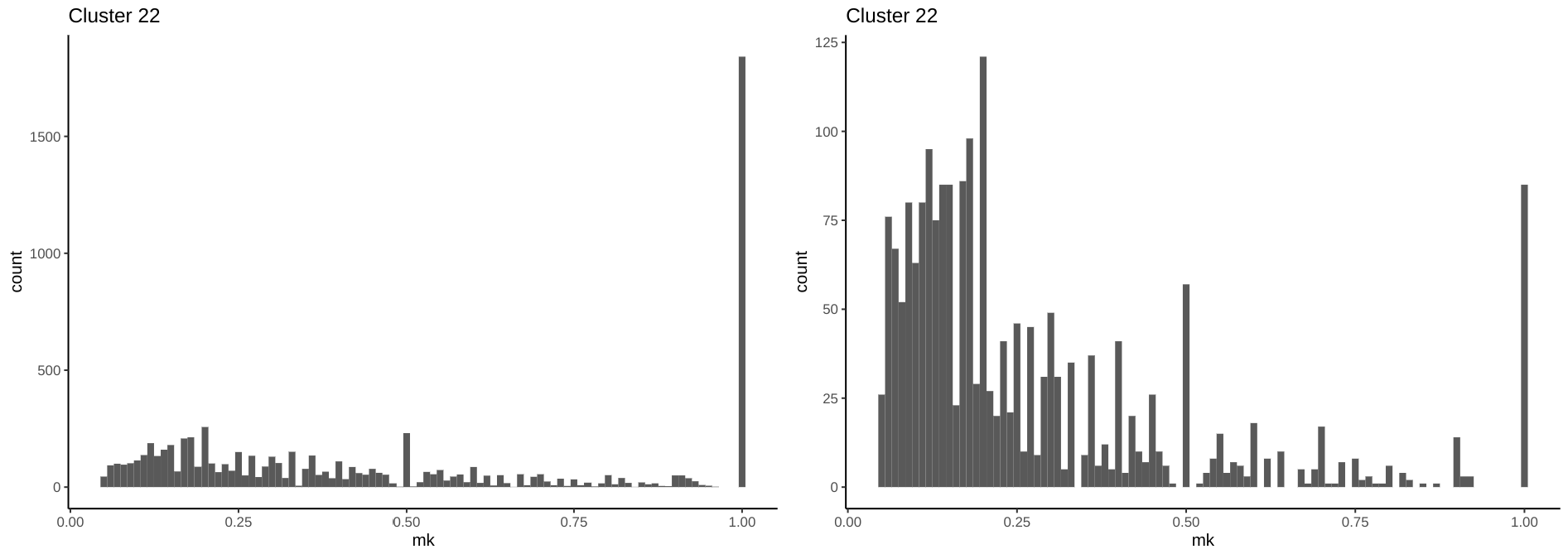
m6A sites E-YTHmut


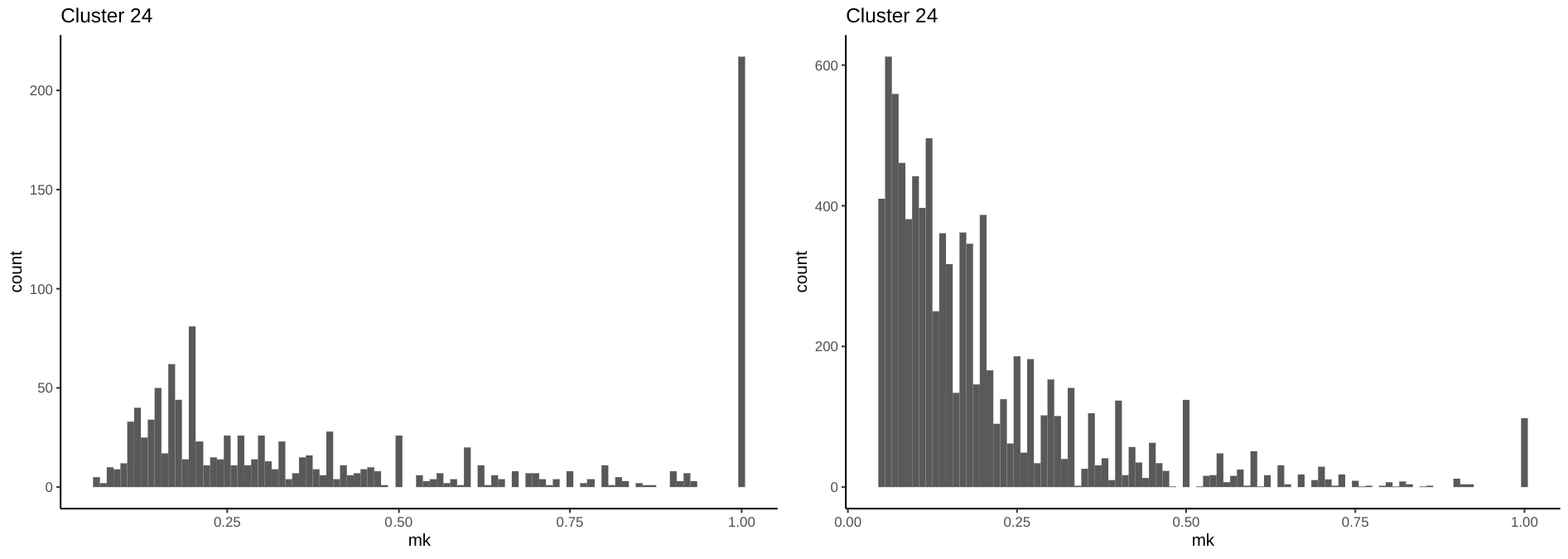
m6A sites E-YTHmut


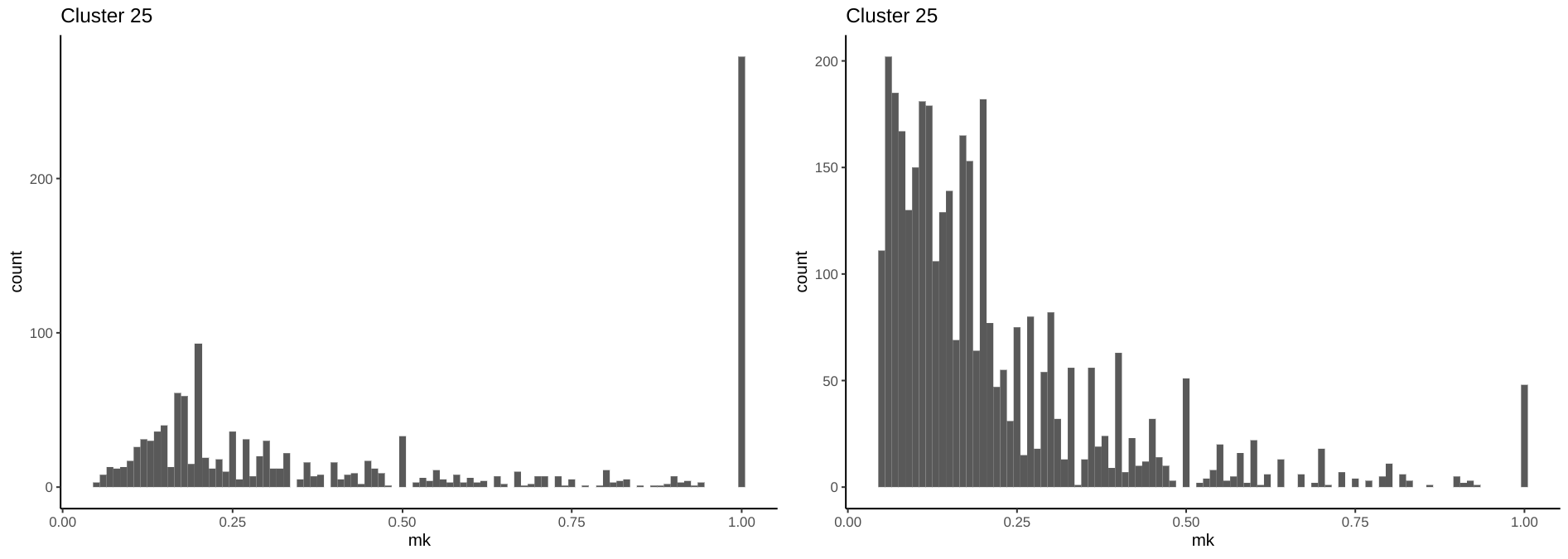
m6A sites E-YTHmut


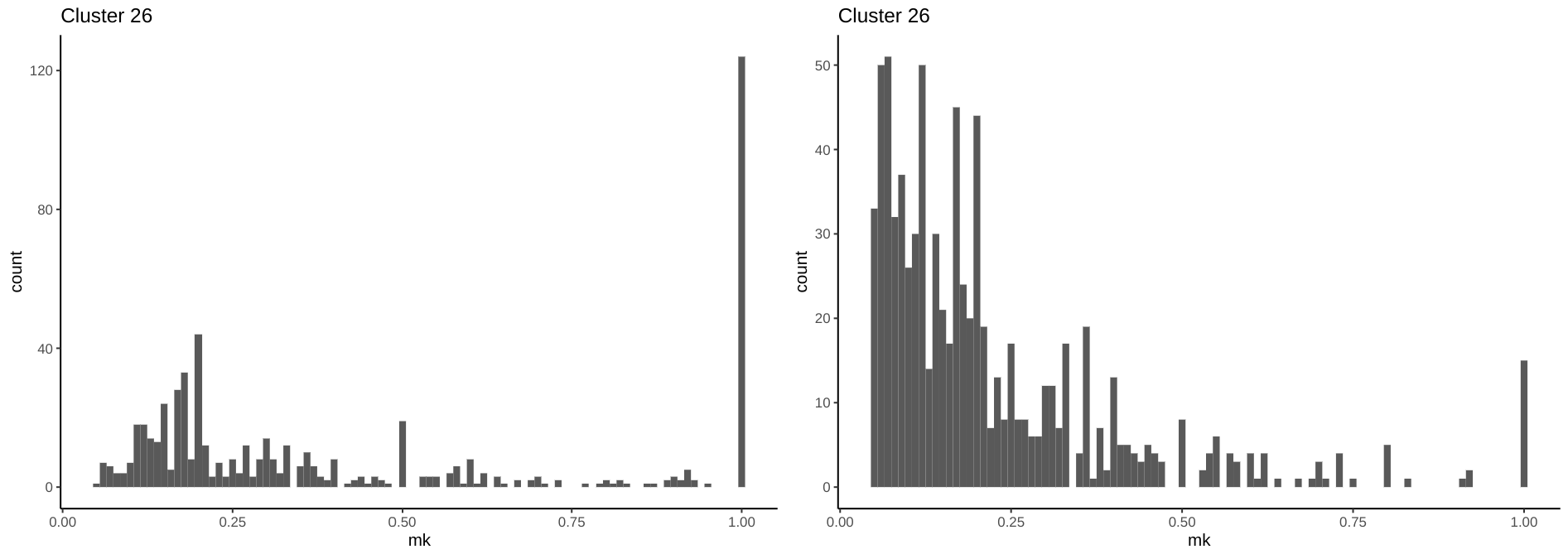
m6A sites E-YTHmut


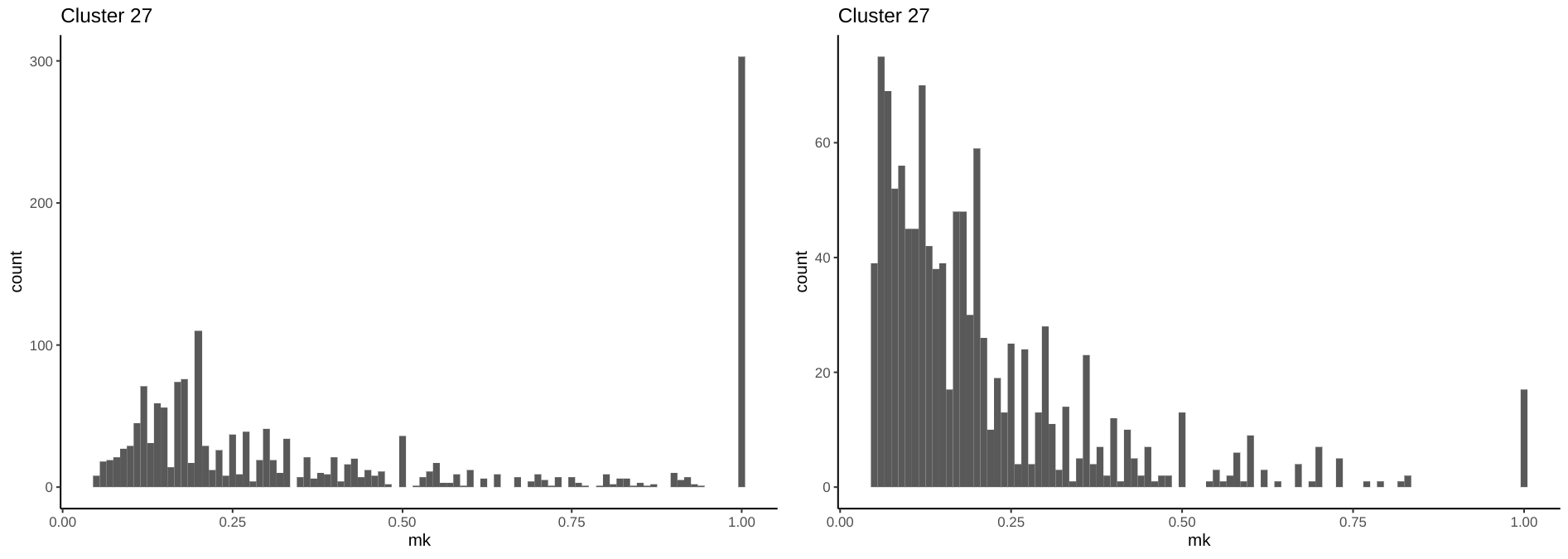
m6A sites E-YTHmut

**Supplemental Fig S7. m^6^A and C-to-U edit counts over mutation per read ratio.**

Histogram of m^6^A site counts over mutation per read (m/k) ratio (left) and histogram of C-to-U edit E-YTH^mut^ background over mutation per read (m/k) ratio (right). The data represents single cell data for OLG, IMC, NEUR, ASC, EC cell lineage cells and for each cluster. OLG: oligodendrocyte cell lineage; IMC: immune cell lineage; NEUR: neuronal cell lineage; ASC: astrocyte cell lineage, EC: endothelial cell lineage. A minimum threshold of 5% was applied.
