# Supplementary material for: Single-cell discovery of m6A RNA modifications in the hippocampus
Source: Genome Res. 2024 Jun;34(6):822–36. doi: 10.1101/gr.278424.123 (PMC11293556; doi:10.1101/gr.278424.123)
Supplement: Supplement 8 [file Supplemental_Fig_S8.docx]

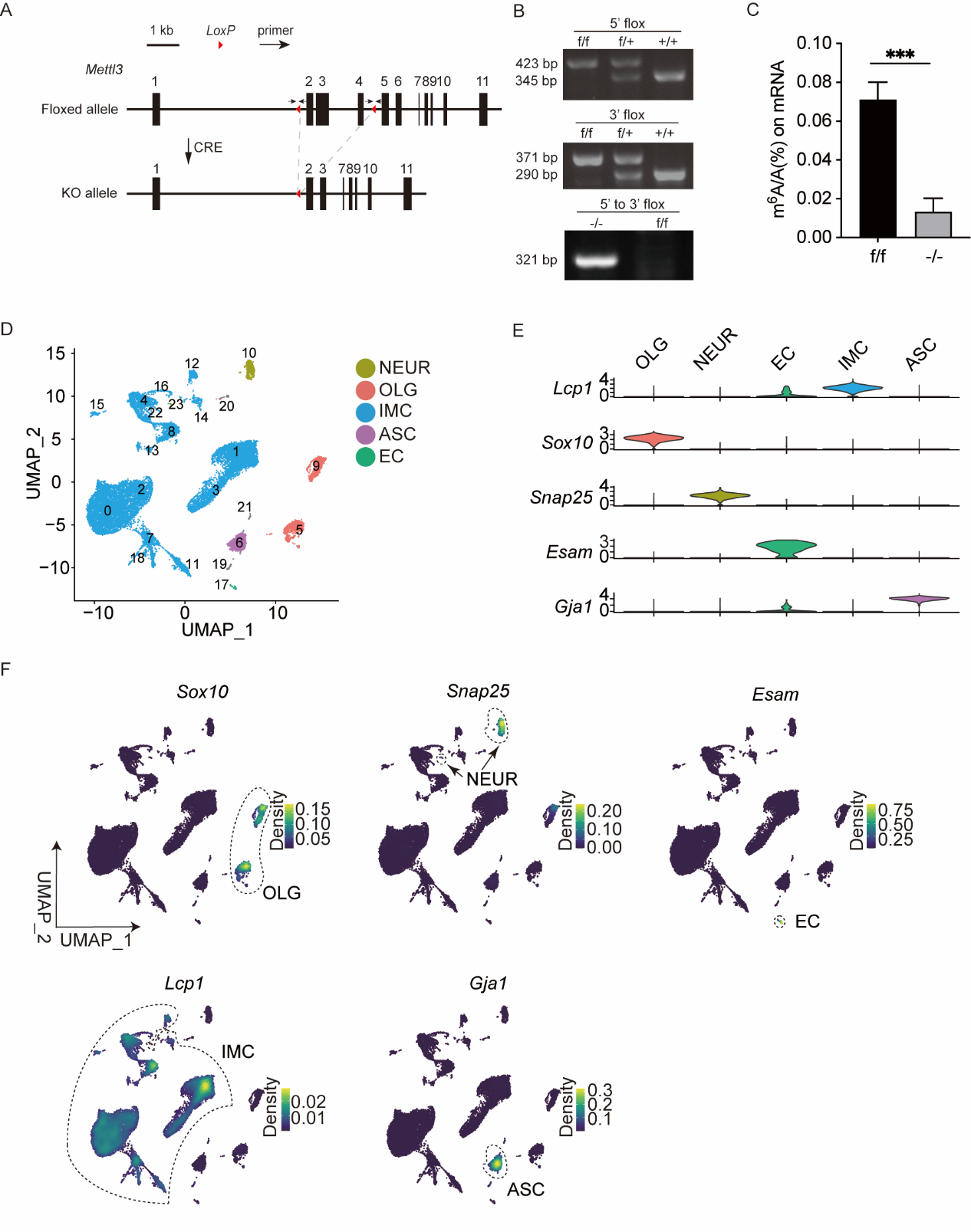


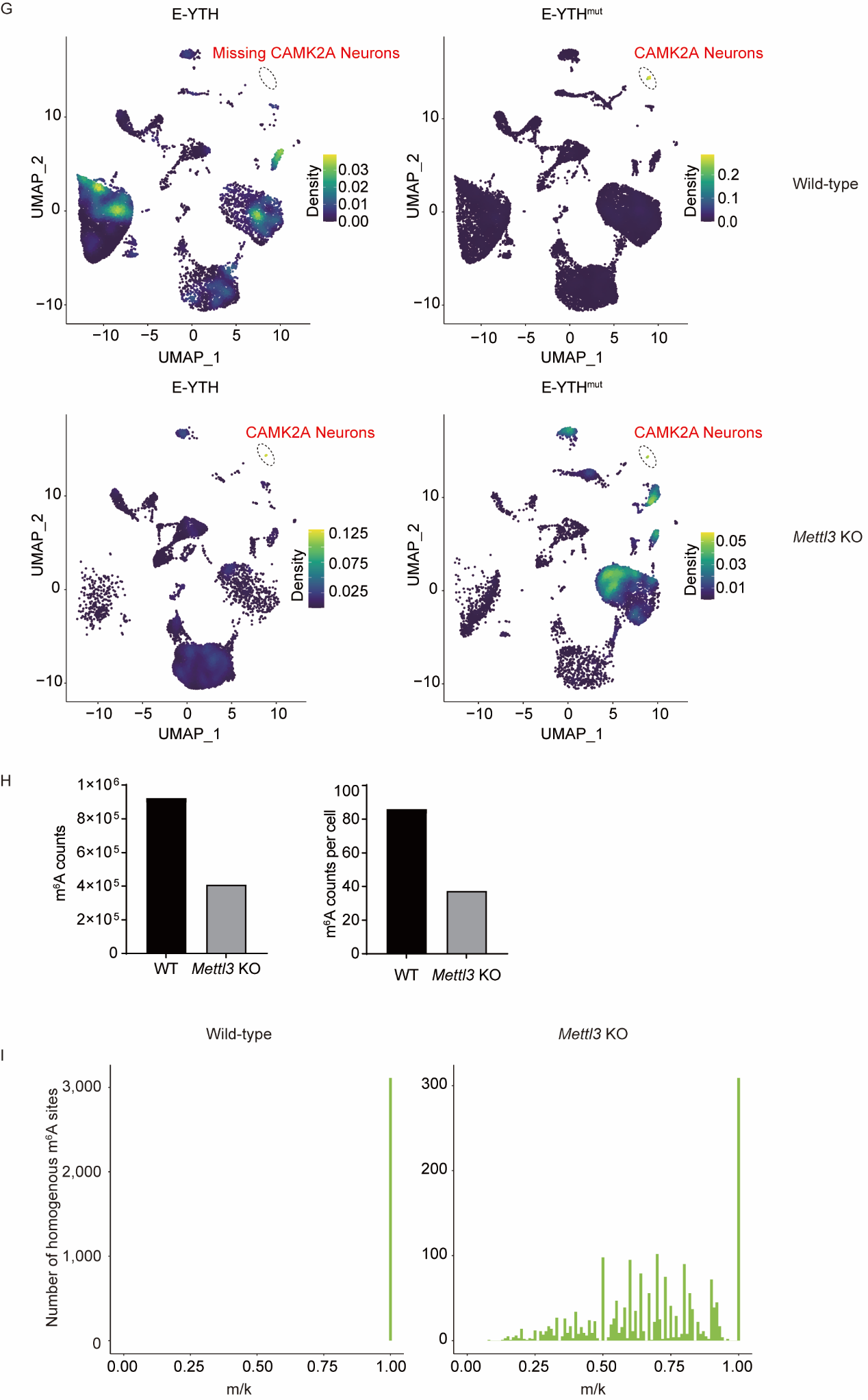


**Supplemental Fig S8. Hippocampal m^6^A characteristics in *Mettl*3 knockout (KO) (*Mettl3^−/−^:Emx1-Cre* ) mice.**

(A) Schematic diagram of *Mettl3^f/f^* mice used to generate *Mettl3* KO mice. The *Mettl3* gene comprises 11 exons, with *LoxP* sites surrounding exons 2 and 4. CRE: CRE recombinase.

(B) Agarose gel electrophoresis image depicts the amplified fragments of the 5' flox region (top), the 3' flox region (middle), and the region from 5' flox to 3' flox (bottom).

(C) Mass spectrometry quantification of m^6^A to unmodified adenosines in mRNA. n=3; two-tailed test. ****P* ≤0.001.

(D) Uniform manifold approximation and projection (UMAP) with 5 main cell type populations identified in *Mettl3* KO mice. NEUR: neuronal cell lineage; OLG: oligodendrocyte cell lineage; IMC: immune cell lineage; ASC: astrocyte cell lineage; EC: endothelial cell lineage; . Numbers indicate cluster number.

(E) Violin plot showing the distribution of expression levels of well-known representative cell-type-enriched marker genes across 5 cell types, 20,471 cells in total.

(F) UMAP with expression levels of cell-type-specific marker genes identifying all 5 major cell populations. Legend colour represents RNA density. Circles were added to visualize grouped cell populations. OLG have high expression of *Sox10*; NEUR have high expression of *Snap25*; EC have high expression of *Esam*; IMC have high expression of *Lcp1*; ASC have high expression of *Gja1*.

(G) UMAP with expression levels of *Camk2a* in E-YTH and E-YTH^mut^ samples for both wild-type and *Mettl3* KO mice. UMAP were derived from the integration of all 4 samples. Circles visualize CAMK2A neurons.

(H) Number of total m^6^A sites and m^6^A sites per cell type detected in wild-type (WT) and *Mettl3* KO mice.

(I) Histograms of m^6^A site counts over mutation per read (m/k) ratio. For wild-type, only m/k=1 sites (homogenous m^6^A) are shown (left). The distribution of these originally m/k=1 (homogenous) m^6^A sites changed in *Mettl3* KO (right).
