# Supplementary material for: Single-cell discovery of m6A RNA modifications in the hippocampus
Source: Genome Res. 2024 Jun;34(6):822–36. doi: 10.1101/gr.278424.123 (PMC11293556; doi:10.1101/gr.278424.123)
Supplement: Supplement 9 [file Supplemental_Fig_S9.docx]

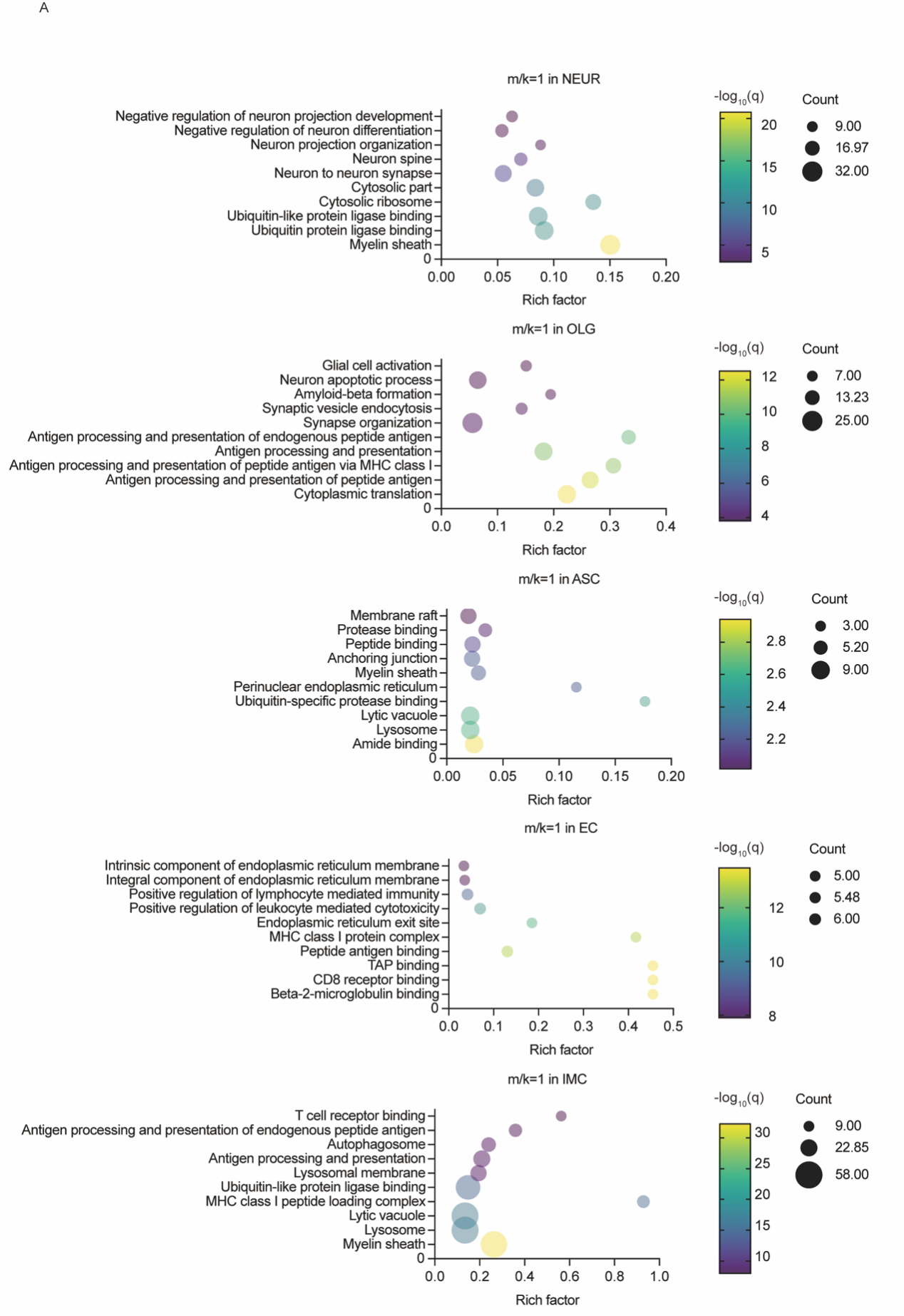


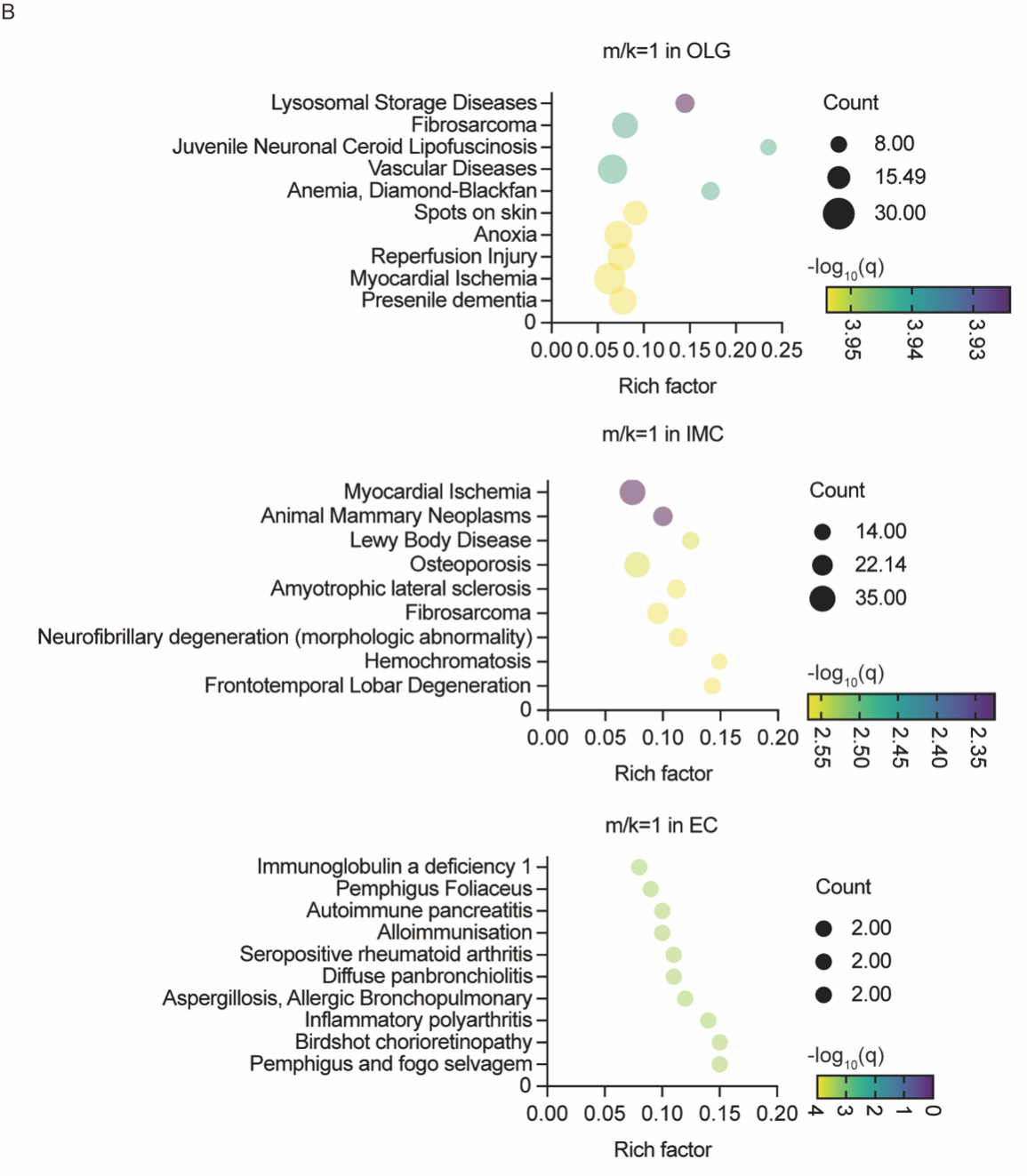


**Supplemental Fig S9. Gene Ontology (GO) analyses of conserved homogenous m^6^A sites.**

(A) Biological process, molecular function, and cellular component GO analyses. Terms with an adjusted *Q* value<0.05 and *P* value<0.05 were considered statistically significant. The top 5 GO terms were plotted for each cell lineage, in addition to 5 selected statistically significant terms. NEUR: neuronal cell lineage; OLG: oligodendrocyte cell lineage; ASC: astrocyte cell lineage; EC: endothelial cell lineage; IMC: immune cell lineage; The Rich factor represent the conserved homogenous m^6^A site count divided by total counts.

(B) Disease GO analyses. Terms with an adjusted *Q* value<0.05 and *P* value<0.05 are plotted for OLG, IMC, EC.
