# Supplementary material for: Single-cell discovery of m6A RNA modifications in the hippocampus
Source: Genome Res. 2024 Jun;34(6):822–36. doi: 10.1101/gr.278424.123 (PMC11293556; doi:10.1101/gr.278424.123)
Supplement: Supplement 10 [file Supplemental_Fig_S10.docx]

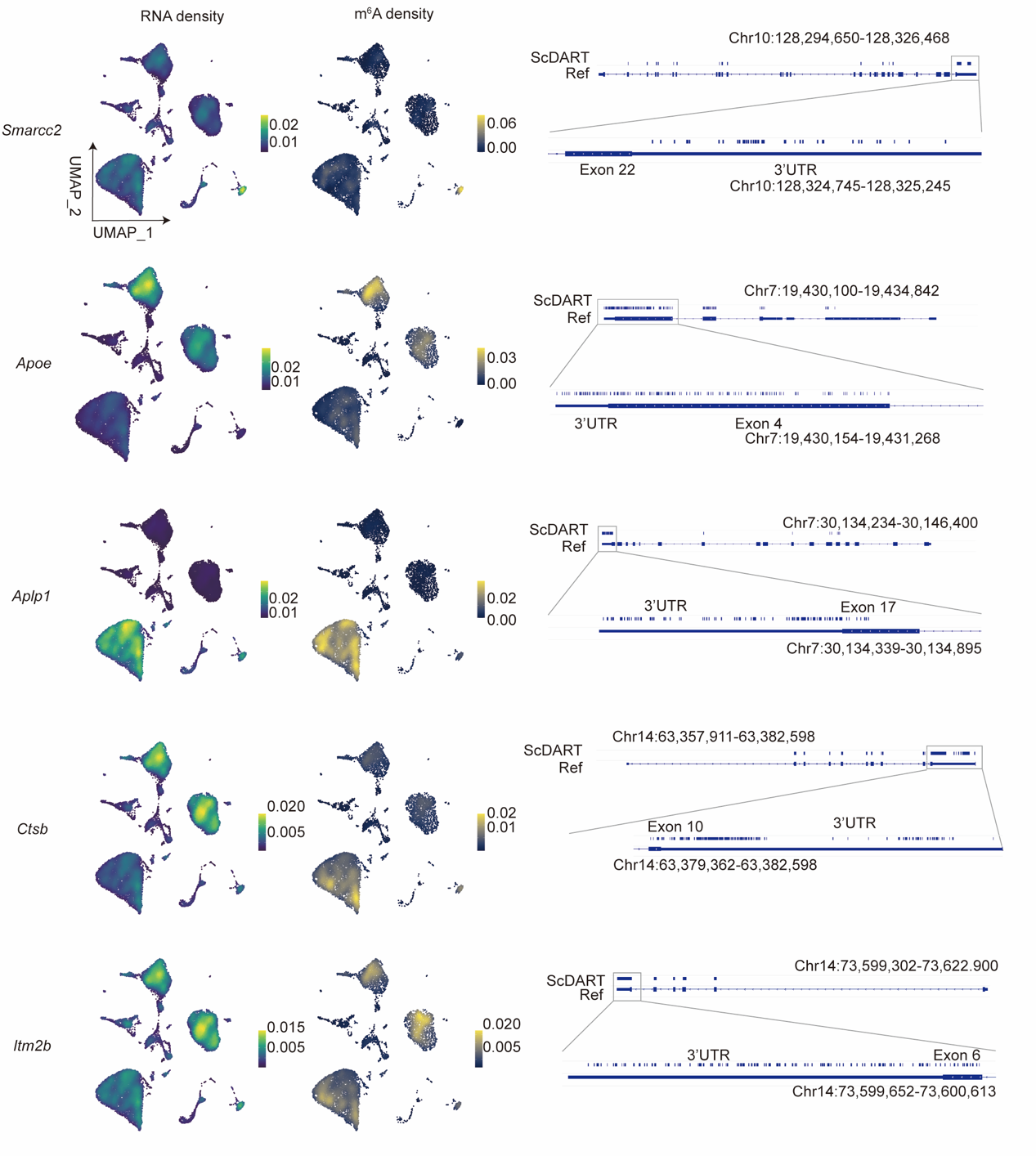

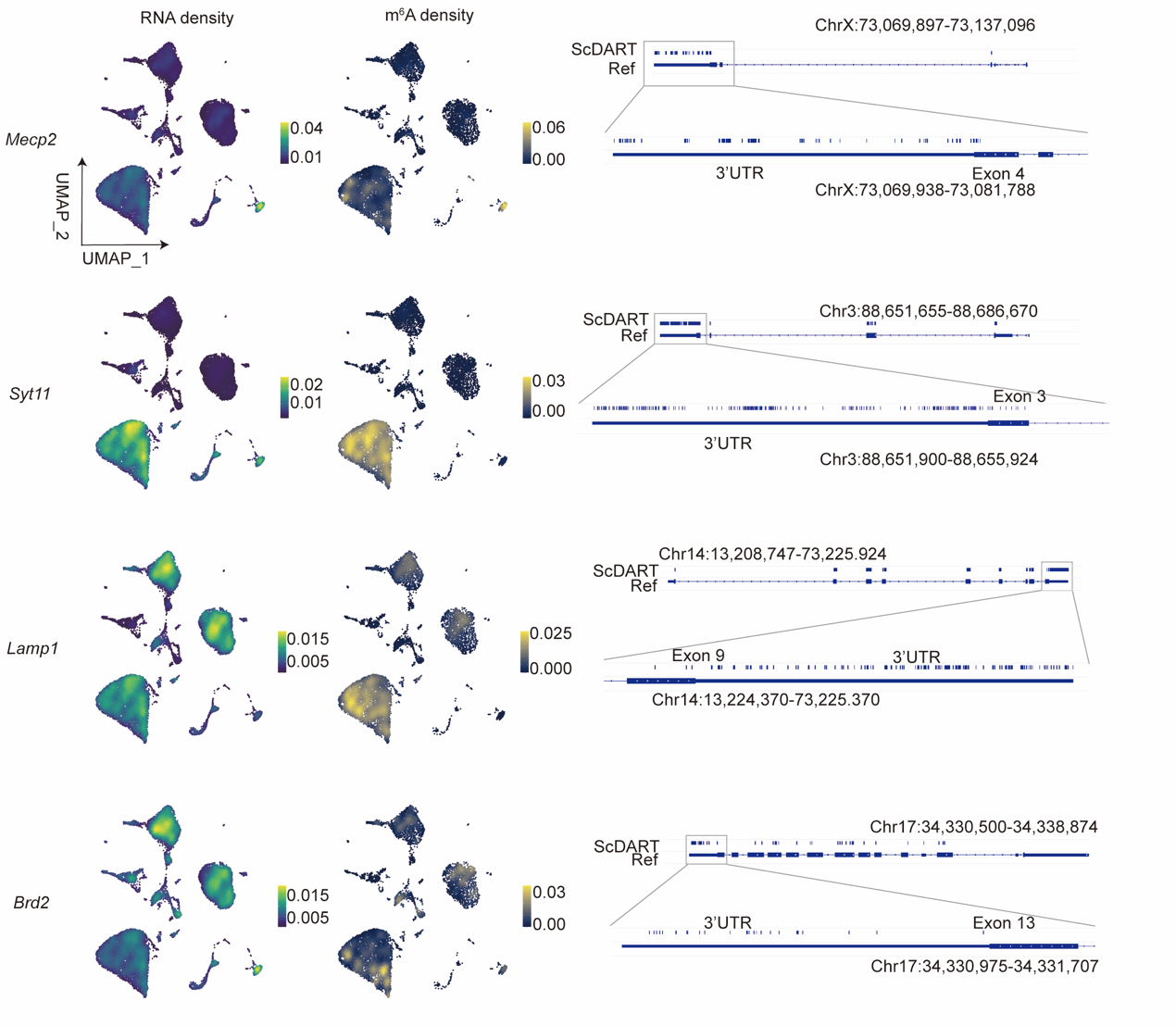


**Supplemental Fig S10. Hippocampal m^6^A single cell distribution in disease associated genes.**

Localizations of m^6^A for different genes. Left: UMAP plot of RNA density for one gene per cell. Legend colour represents RNA density. Middle: UMAP plot of m^6^A density for one gene per cell. Legend colour represents m^6^A density on RNAs transcribed from one gene. Right: IGV RefSeq gene annotations with editing sites representing adjacent m^6^A sites is shown. Last exon with 3’UTR region is illustrated with higher magnitude. Chr: Chromosome number; Units: base pairs.
